# Estimation of membrane bending modulus of stiffness tuned human red blood cells from micropore filtration studies

**DOI:** 10.1371/journal.pone.0226640

**Published:** 2019-12-31

**Authors:** Rekha Selvan, Praveen Parthasarathi, Shruthi S. Iyengar, Sharath Ananthamurthy, Sarbari Bhattacharya

**Affiliations:** 1 Department of Physics, Bangalore University, Bangalore, India; 2 Soft Condensed Matter Group, Raman Research Institute, Bangalore, India; 3 School of Physics, University of Hyderabad, Hyderabad, Telangana, India; University of New South Wales, AUSTRALIA

## Abstract

Human red blood cells (RBCs) need to deform in order to pass through capillaries in human vasculature with diameter smaller than that of the RBC. An altered RBC cell membrane stiffness (CMS), thereby, is likely to have consequences on their flow rate. RBC CMS is known to be affected by several commonly encountered disease conditions. This study was carried out to investigate whether an increase in RBC CMS, to the extent seen in such commonly encountered medical conditions, affects the RBC flow rate through channels with diameters comparable to that of the RBC. To do this, we use RBCs extracted from a healthy individual with no known medical conditions and treated with various concentrations of Bovine Serum Albumin (BSA). We study their flow through polycarbonate membranes with pores of diameter 5*μ*m and 8*μ*m which are smaller than and comparable to the RBC diameter respectively. The studies are carried out at constant hematocrit and volumetric flow rate. We find that when the diameter of the capillary is smaller than that of the RBC, the flow rate of the RBCs is lowered as the concentration of BSA is increased while the reverse is true when the diameter is comparable to that of the RBC. We confirm that this is a consequence of altered CMS of the RBCs from their reorientation dynamics in an Optical Tweezer. We find that a treatment with 0.50mg/ml BSA mimics the situation for RBCs extracted from a healthy individual while concentrations higher than 0.50mg/ml elevate the RBC CMS across a range expected for individuals with a condition of hyperglycemia. Using a simple theoretical model of the RBC deformation process at the entry of a narrow channel, we extract the RBC membrane bending modulus from their flow rate.

## Introduction

Human blood consists of plasma, red blood cells (RBCs), white blood cells (WBCs) and platelets. RBCs form the major cellular component of blood with a ratio of RBC volume to the total volume of blood (hematocrit value) of approximately 45% [1]. RBCs can be described as bi-concave disks with diameter that lies in the range 6.2—8.2*μ*m and thickness that varies along a diametrical line from about 1*μ*m at the center to a maximum of about 2*μ*m close to the edge. The most important function of RBCs is to carry oxygen to various parts of the body. The viscoelastic properties of RBCs are of extreme importance here as RBCs, in their lifespan of approximately 120 days [2], squeeze through capillaries with diameters much smaller than their own without compromising on their functionality. When passing through narrow channels they undergo extensive deformation into axisymmetric (parachute) shapes [3, 4].

The deformation process that an RBC undergoes at the entrance of a narrow capillary, finally enabling passage through it, is a gradual process [5]. Each RBC requires a finite amount of time to deform enough to enter a narrow channel. This deformation time, of course, depends on the RBC cell membrane stiffness (CMS), the channel size as well as the blood flow rate. Given a blood flow rate and channel size, elevated RBC CMS would mean increased deformation time. This implies a reduced rate of RBC flow in the capillaries. It is known that oxygen diffusion from RBCs to surrounding tissues happens only in the micro circulation [1]. Any elevation in the CMS of RBCs can, thereby, alter their passage time in micro circulation consequently also affecting the rate of oxygen supply to the tissues. It is known that medical conditions like hyperglycemia [6–8], jaundice [9], malaria and sickle cell disease [10] elevate RBC CMS. It is important to study the effect of altered RBC CMS on the RBC micro circulation and its possible connection to the symptoms and complications that are associated with these medical conditions. Establishing a cause and effect chronology here could help in early diagnostics and perhaps, more effective treatment.

Studies on viscoelastic properties of RBCs involve either observing the response of an RBC subjected to a well calibrated deforming stress or an analysis of the detailed observation of a process where RBC deformations occur. These have been widely reported in literature and involve a wide variety of techniques based on micro pipette aspiration [11–13], optical tweezers (reorientation [14], stretching [15–17]), observation of membrane fluctuations [18, 19], optical stretcher [20, 21] and micro fluidic channel flows [3, 22, 23]. Though all these techniques, in principle, provide a measure of the RBC CMS, the stress regimes in which the experiments are done are extremely different and can lead to a varied conclusion on the viscoelastic response of the RBC. Techniques like micro pipette aspiration and optical stretcher involve high stress and often use osmotically swollen spherical RBCs. Extensional flows in microfluidic channels [23] operate at high stress regimes to be able to produce a large throughput and visible deformations of the cells. Such stress regimes are, however, much higher than what is encountered by RBCs in vivo. In Atomic Force Microscopy based techniques [24, 25], RBCs are usually treated with chemicals like glutaraldehyde and polylysine that are required for fixation of RBCs. The effect of these chemicals [22, 26] on the RBC CMS are generally neglected leading to values of CMS that may not be the same as that of RBCs in their native state. So, if the main objective is to understand the flow properties of RBCs in vivo and the factors that can affect it, measurements that are carried out on structurally unaltered RBCs in the stress regime that RBCs are subjected to while flowing through human vasculature are of most value and relevance. The stress regimes involved in techniques like membrane fluctuation detection, passage of RBCs through micro porous channels [27–31], and optical tweezer based reorientation techniques can be considered to be low stress as the forces involved here are of the order of a few piconewtons (pN).

Apart from the difficulties in settling on an appropriate experimental method that satisfies the criterion outlined above, there are even greater difficulties if RBCs with altered CMS are sourced from individuals with medical conditions known to affect RBC CMS. Donors affected with a particular medical condition need to be free of all other medical disorders which could have an impact on the RBC CMS in order to be able to establish a connection between the extent of the disorder and its consequences due to altered micro circulation in the human vasculature. Using such RBCs for the testing or calibration of any alternate measurement process of RBC CMS is thereby also not advisable. A necessary first step in this direction, then, would be to arrive at some artificial means of elevating the CMS of RBCs extracted from healthy individuals with no known medical conditions, in a controlled manner. The method of altering the CMS should preferably be such that it would be possible to mimic any RBC CMS associated with a medical disorder by varying a single parameter in a continuous manner.

Our aim here is to study the effect of RBC CMS elevation to the extent seen in commonly encountered human disease conditions on the flow in channels of sizes commonly encountered in human micro vasculature and we use treatment with Bovine Serum Albumin (BSA) as the means of bringing about the required change. BSA is a serum albumin protein that is extracted from bovine blood. The use of BSA for preparation of RBC samples is widely quoted in literature. BSA is generally used in in-vitro studies of RBCs to enable a retention of the normal bi-concave shape and to prevent adhesion to glass slides [17, 32]. There are studies [33, 34] that report geometrical shape changes in RBCs and enhancement of the RBC CMS under BSA action ascribing these effects to the replacement of human serum albumin (HSA) by BSA from various sites on the membrane. Reference [34] also reports that RBCs treated with BSA show much higher mechanical resistance to lysis when compared to those treated with HSA at an identical concentration. RBCs extracted from individuals with no known medical conditions (nRBCs) are treated with BSA at various concentrations to obtain bRBCs. Our idea behind using BSA to tune the CMS of nRBCs is that our results can be interpreted in terms of CMS change alone. We find that the flow rate of RBCs is clearly affected by BSA treatment, the exact manner of change depending on whether the RBC diameter is larger or smaller than the channel diameter. From these results, we obtain an estimate of membrane bending modulus of bRBCs using a simple theoretical model that assumes an axisymmetric shape on deformation and imposes a constraint of constant surface area throughout the deformation process.

## Materials and methods

### Preparation of PBS + BSA suspension

Phosphate Buffer Saline or PBS (200ml of deionized water with NaCl (1.602g), KCl (0.040g), *Na*_2_ *HPO*_4_ (0.356g) and *KH*_2_ *PO*_4_ (0.054g)) of pH 7.4 was prepared using a standard protocol and used for the experiments. The pH of PBS prepared was checked at regular intervals. If there was any change, care was taken to restore the pH to 7.4. Note that for a comparative study to be valid, it is important that the same solution of PBS is used to carry out all experiments. This is because minor variations in the osmolarity of the PBS could have an effect on the measurements [14]. The suspensions with BSA were prepared not more than an hour prior to the commencement of the experiment. These suspensions were prepared by weighing the required quantity of BSA (A2058-1G, Sigma-Aldrich) on a micro balance and adding it to a measured volume of PBS. The resultant mixture was then stirred till the BSA dissolved completely. Usually, 50 ml of PBS + (x)BSA, where x stands for the concentration of BSA in mg/ml, was prepared at a time and used for the experiments.

### Regulations and protocol followed for RBC extraction and treatment

All methods were carried out in accordance with the relevant guidelines and regulations of the World Medical Association Declaration of Helsinki. The proposal for this study, as well as subsequent progress reports, have been examined and approved by the Board of Studies and the Ph.D. Review Committee of Department of Physics, Bangalore University, Bangalore. The experiments were conducted with the informed consent of all participants.

Four healthy individuals in the age group of 25–50 years with no known medical conditions participated in blood donation for experiments. However, the data used for analysis and shown here are with the blood samples obtained from the same individual (collected on several occasions in a time period spanning over a year). There were no significant changes observed in the trends of primary results for all individuals in both micropore filtration as well as reorientation experiments.

All the experiments were carried out within four to five hours of the time of blood extraction. This was done to avoid any ambiguity in the interpretation of results that could arise due to CMS changes linked with storage time of the RBCs.

### Micropore filtration: Preparation of b(x)RBC samples

5ml of venous blood was drawn from a healthy volunteer with no history of any chronic medical conditions. This was transferred from the collection syringe to commercially available blood collection tubes coated with Ethylenediaminetetraacetic acid (EDTA) (1.8mg/ml, *BD Vacutainer*) and centrifuged at 900 rpm for 10 minutes. The plasma and the top white layer containing WBCs and platelets were discarded. RBCs thus obtained were washed with PBS and about 200*μ*l of RBCs were added to 3800*μ*l of PBS+(x)BSA to obtain b(x)RBCs. This particular ratio ensures a 5% hematocrit level. We thus obtain b(x)RBCs for x = 0.50, 0.70, 1.00, 1.18 and 1.35. Note that BSA concentrations less than 0.50mg/ml were not used for flow experiments. It was observed that for a hematocrit level of 5% the biconcave shape could not be retained for all RBCs at BSA concentrations less than 0.50mg/ml.

### Micropore filtration: Set up and description

**Fig 1:**
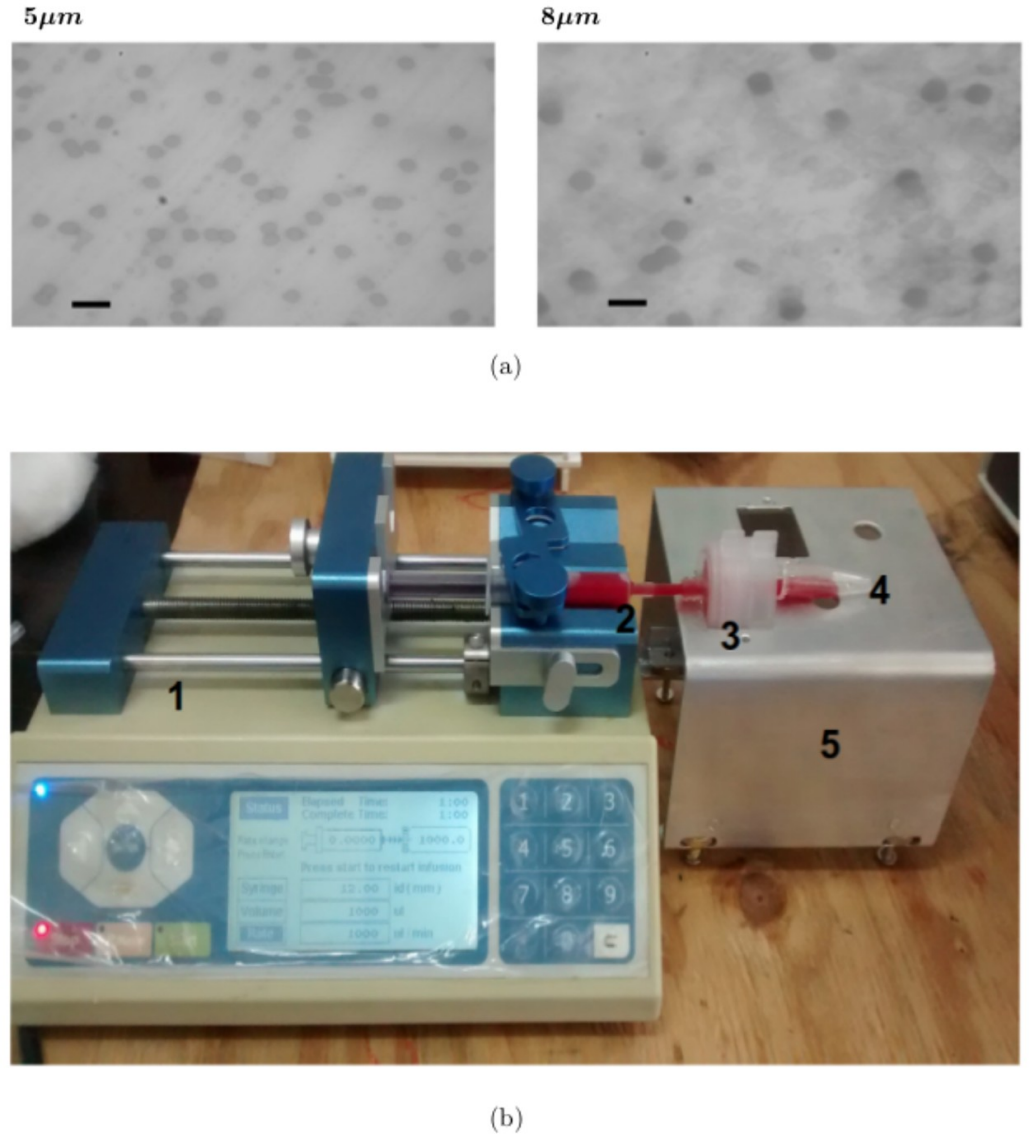
(a) Photographs of cyclopore filter papers taken using an optical microscope with 40X optical zoom. The legends on the photographs are the pore diameters. A scale of 10*μ*m is indicated by a solid black line. (b) Experimental set up for monitoring the flow of CMS altered RBCs through narrow channels.1: Syringe pump, 2: Diluted blood sample in syringe, 3: Filter holder with filter paper placed inside, 4: Micro centrifuge tube, 5: Custom made aluminium stand with height adjustment screws.

Whatman Cyclopore filter papers used for this experiment are essentially poly carbonate membranes (*WHA70622513* and *WHA70604714*, Sigma—Aldrich) with pores that are ion-track etched. The pores are of circular cross section with a well-defined uniform diameter throughout their length and run straight and perpendicular to the plane of the filter paper [35]. We have used cyclopore filter papers of diameters (i) 5*μ*m (less than RBC diameter) and (ii) 8*μm* (≈ RBC diameter) for the experiment (Fig 1a). Number of pores per unit cross sectional area (pore density) of the two different filter papers were estimated from 200 different scanned images of the filter paper, each image corresponding to an area that was 10,000 times larger than the cross sectional area of a single pore. The pore density of the 5*μ*m and 8*μ*m filter papers were found to be 4523 ± 473 pores/*mm*^2^ and 1244 ± 78 pores/*mm*^2^ respectively. Using density of polycarbonate, dry mass of the filter paper and porosity values, the thickness of the 5*μ*m and 8*μ*m filter paper were estimated to be 12*μ*m and 10*μ*m respectively. It is evident from Fig 1a that there is some probability of finding overlapping pores. For estimating the pore size distribution and probability of presence of isolated pores, 100 different scanned images, each corresponding to an area 1000 times the cross sectional area of a single pore (recorded using a higher optical zoom) were used. The diameter of the pores were found to be 5.21 ± 0.4*μ*m in the 5*μ*m filter paper and 7.92 ± 0.7*μ*m in the 8*μ*m filter paper. The probability of finding isolated pores were about 85% and 90% in the 5*μ*m and 8*μ*m filter papers respectively.

The filter paper was mounted on a filter paper holder (Whatman plastic filter holder *WHA420200*) and placed on a custom made aluminium stand fitted with height adjustment screws. About 4ml of the desired blood sample was loaded into a 5ml syringe and mounted on the syringe pump (Chemyx Fusion 200). The syringe was connected to the inlet of the filter holder and the outlet was connected to a micro centrifuge tube with a transparent tube procured from commercially available blood transfusion kits (see Fig 1b).

The syringe pump was set at a flow rate of 400*μ*l/min to ensure that the flow velocities encountered were in the range found in blood capillaries [36, 37]. At the inlet, maximum velocity is estimated to be 1 mm/s. The blood sample from the syringe flows through the cyclopore filter paper and is collected in the collection tube for an interval of 60s. For each BSA concentration, four such output samples were collected sequentially. The output samples so collected were labelled accordingly and stored in the refrigerator (at 4°C) until the analysis. A fresh syringe, fresh filter papers and fresh holders were used for every trial. This minimizes the possible problem of blocking of pores. Apart from this, all other conditions were maintained constant. The experiments were carried out for each b(x)RBC with both filter papers. The output samples were imaged with an optical microscope using a 100X zoom. Care was taken to see that the RBC shapes were normal and that no lysis had taken place in the filtration process.

### Micropore filtration: RBC concentration determination

The cell concentration at the input and output stage of the experiment were obtained using manual counting on a hemocytometer (Neubauer chamber). About 10*μ*l of each output sample diluted to a carefully controlled amount was loaded onto the hemocytometer kept under a upright optical microscope with a 40X zoom. Nearly 200, out of a total of 400 smaller grids, spanning the entire central region of the Neubauer chamber were photographed, saved and used for the analysis. This was done for each of the output samples. We use the cell counts to obtain the number of RBCs per unit volume at the input (*N*_*in*_) and the output (*N*_*out*_) stages, *N*_*out*_ being the average count of all the output samples collected for a particular BSA concentration. While *N*_*in*_ values are obtained to ascertain that the flow experiments for all b(x)RBCs have been done under identical input conditions, the effect of altered RBC CMS on the flow rate is in the value of *N*_*out*_.

### Assessment of RBC CMS using RBC reorientation in optical tweezers

#### Preparation of b(x)RBC samples

50*μ*l of blood was drawn from a person with no known medical conditions using finger prick method. EDTA at a concentration of 1.8mg/ml was added to this to prevent agglomeration. The end product was further diluted with about 1ml of normal PBS of pH 7.4. The set of experiments carried out with these were taken as the control (nRBC).

For the preparation of b(x)RBCs (x = 0.10, 0.35, 0.50, 0.70, 1.00, 1.35 and 1.75mg/ml), 100*μ*l of blood was drawn from the same individual and transferred into a centrifuge tube with EDTA (at the same concentration as used for nRBC sample preparation). It was diluted with normal PBS and centrifuged for 10 minutes at 900 rpm. The supernatant was then removed and suspended in a tube containing PBS+(x) BSA. The sample was labelled as b(x)RBC. In each of the samples, the RBCs were maintained at a hematocrit level of 5%. In each case, about 300*μ*l of PBS+(x)BSA and 20*μ*l of b(x)RBCs were loaded into a sample well that was made by gluing an O ring of diameter 0.8cm and thickness 1mm on a glass slide. The well was covered with a smaller cover slip to make it air tight.

#### Measurement of time of reorientation

A detailed description of the optical tweezer set up used for this experiment can be found in Parthasarathi *et al* [14], however a brief description can be found in S1 File. When an RBC is trapped in a Gaussian profile single beam optical trap, it reorients such that its volume is maximized in the region of maximum light intensity, here along the direction of propagation of light. The time of reorientation, *t*_*re*_, defined as the time taken by an RBC which is initially with the plane of its disc perpendicular to the beam propagation direction (flat orientation) to rotate by 90° and acquire an edge-on orientation to the same, depends on the laser power as well as the RBC CMS [14]. Note that it is essential to have the laser beam on to keep the RBC in the edge-on orientation. When the supply of energy is cut off, either by blocking the laser beam or turning it off, the RBC reorients and settles, under the effect of gravity, to the flat-orientation seen prior to trapping.

Video recordings of the reorientation process in the tweezer were carried out at the rate of 16 fps using a CCD camera (Point Grey Research, Blackfly). The power at the sample plane was varied from 0.002W to 0.040W in steps of 0.002W. Powers higher than 0.040W were avoided as permanent damage to the RBCs at high laser power is inevitable. At each power, reorientation process of the control and 7 sets of b(x)RBCs were recorded and stored for further analysis. For each type of RBC, the reorientation process was recorded for at least ten different RBCs at each laser power. The time of reorientation, *t*_*re*_, was extracted from an analysis of the video recordings and an average *t*_*re*_ was found for each laser power used, with the standard deviation being taken as the error in *t*_*re*_.

## Results, analysis and discussion

### Variation of RBC flow rate with BSA concentration

**Fig 2:**
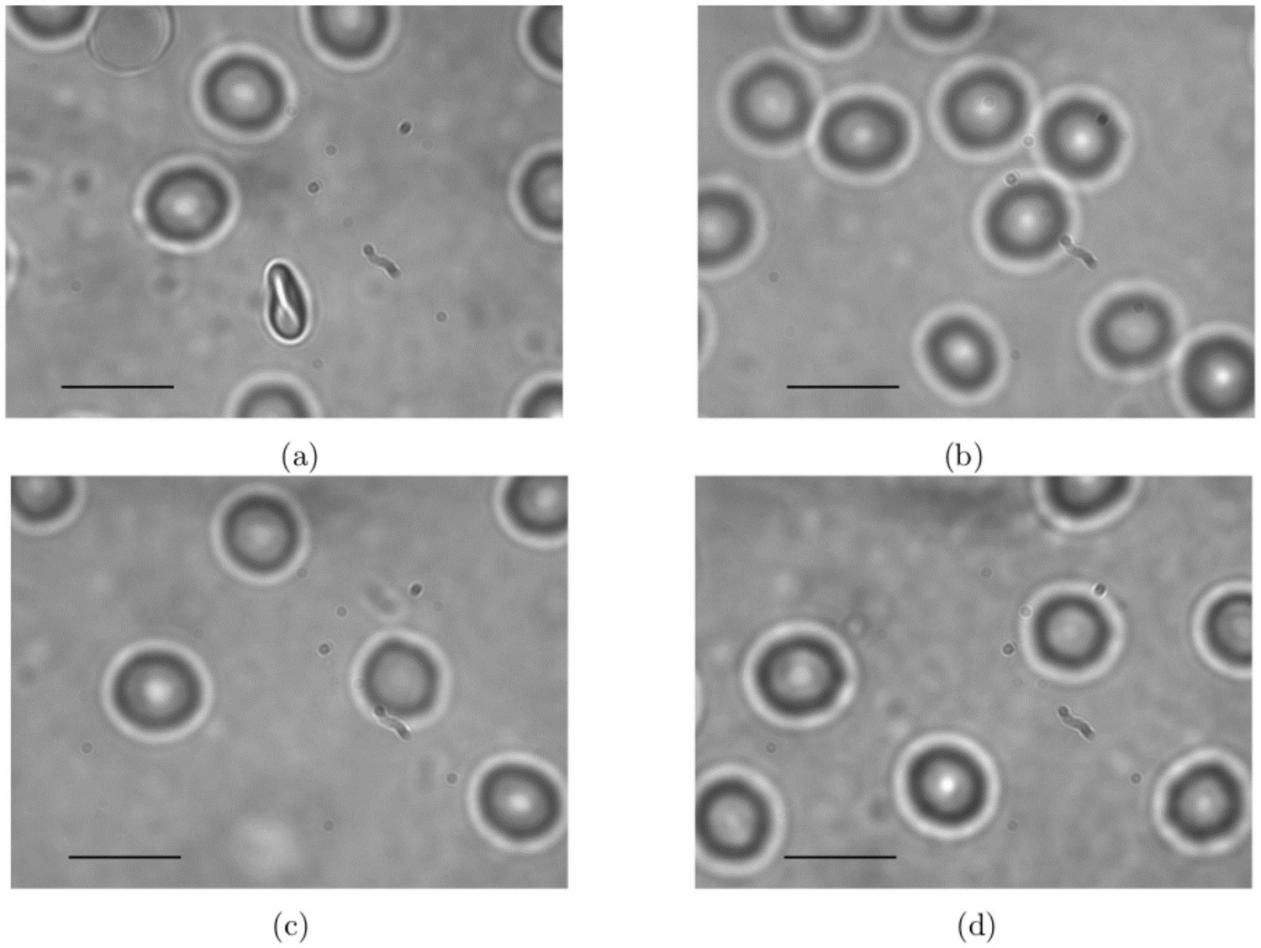
Snapshots of (a) nRBC (an RBC that has reoriented itself along the laser propagation direction is also seen here), (b) b(0.5)RBC, (c) b(1.35)RBC and (d) b(1.75)RBC taken from the reorientation videos. There are no visible changes in the size or appearance of RBCs treated with different concentrations of BSA. A scale of 10*μm* is indicated by a solid black line on all the photographs.

RBCs do not show any detectable change in their size distribution with the BSA concentration they are treated with (see Fig 2). At all concentrations, the mean diameter of RBCs was found to be centered around 7.6*μm* with a standard deviation of 0.5-0.9*μm* (see Table 1, the size distribution was obtained by analysing an average of 120 RBCs at each concentration). Channels with diameters as small as 3*μm* are found in human vasculature and RBCs pass through these without undergoing hemolysis. We thereby do not expect RBCs to undergo lysis while passing through the cyclopore filters used in our experiment as both pore diameters as well as flow velocities are comparable to that found in vivo. Further, the capillary number (*Ca*) of nRBCs is estimated to be ≈10^−4^ in the active region (which we define as the region of the filter paper aligned with the inlet of filter holder through which practically all the filtration happens), 0.87 in 5*μm* pore and 0.62 in 8*μm* pore where *Ca* is given by

$$\begin{array}{c} Ca=\frac{\eta \dot{\gamma }l}{E_{s}}, \end{array}$$ (1)

*η* being the viscosity of the fluid, $\dot{\gamma }$, the shear rate, *l* = 2.78*μm*, the radius of a sphere of volume equal to that of an RBC (90fL) [2] and *E*_*s*_, the RBC membrane shear modulus (≈ 4*μN*/*m*) [38]. It is known that hemolysis does not occur at such low capillary numbers [38].

**Table 1:** Diameters of various b(RBCs).

b(x)RBC	Diameter (*μm*)
*Control*	7.64 ± 0.57
*b*(0.50)*RBC*	7.51 ± 0.84
*b*(1.35)*RBC*	7.66 ± 0.52
*b*(1.75)*RBC*	7.61 ± 0.62

For reasons detailed above, the fact that the RBC suspension is pumped continuously at a constant flow rate with a constant hematocrit of 5% at the input stage and that care is taken to both not re use the filter papers as well as not run the flow for more than four minutes at a stretch, the chances of RBCs blocking the pores during the course of the experiment is very minimal. Thereby a reduction in the number of channels during the process of filtration is unlikely. Any change in the number of RBCs collected per unit time at the output stage can be, thereby, attributed to a change in their rate of transfer from the input to the output stage and not because of blocked pores or RBC loss due to lysis.

To enable a comparison in the flow rate of various bRBCs, we first determine the number of RBCs that have passed through the filter paper per unit time per pore, N*, from *N*_*out*_ using Eq 2

$$\begin{array}{c} N^{*}=\frac{N_{out}Q}{A_{active}N_{pore}} \end{array}$$ (2)

where *A*_*active*_ is the area of the active region. The filter holder used for the experiment has an inlet of inner radius 0.2cm giving us an active area *A*_*active*_ = 12.56*mm*^2^. *N*_*pore*_ is the number of pores per unit area of the filter paper and *Q* is the volumetric flow rate. As we use a *Q* of 400*μ*l/minute here, N*, corresponds to the number of RBCs that have passed through a single pore in one minute.

**Fig 3:**
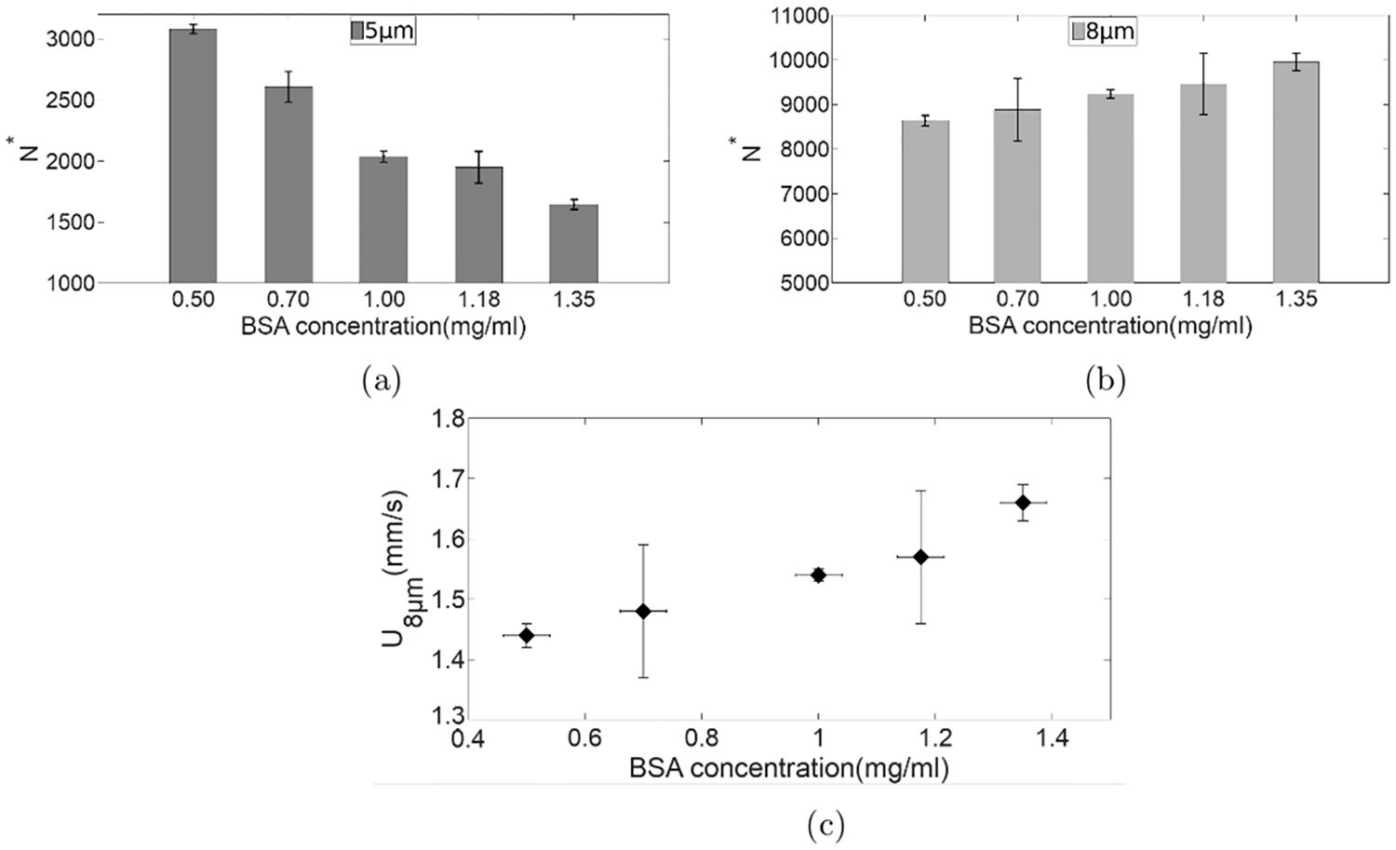
(a) and (b) Number of RBCs obtained in the output per unit pore per minute (N*) of various bRBCs studied. (c) Passage velocities of flow of various bRBCs through 8*μ*m diameter channels.

We find that N* decreases with increasing BSA concentration for the 5*μ*m filter paper (Fig 3a) while it increases for the 8*μ*m filter paper (Fig 3b). As RBCs treated with BSA show an increased resistance to lysis due to an increase in the mechanical strength of RBC [34], it can be conjectured that bRBCs become stiffer with increase in BSA concentration. Stiffer RBCs would require more time to deform to pass through 5*μ*m pores leading to a decreased number of RBCs flowing out through each pore per minute. This is indeed what is observed for micropore filtration through the 5*μ*m filter paper. The filtration results with the 8*μ*m filter paper, however, appear counter intuitive to this line of reasoning as the number of bRBCs flowing out through each pore per minute increases with increase in the BSA concentration (Fig 3b). We need to recall that for the 8*μ*m filter paper, the RBC diameter is approximately same as the channel diameter, thereby requiring no additional deformation time to gain entry into these pores. The result suggests that the passage velocity of bRBCs increases with the increase in BSA concentration leading to larger RBC transfer rate per pore. This is probably because the RBCs stiffened with the use of BSA feel a lower translational drag when compared to their less stiff counterparts.

While the lowering of translational drag with increase of BSA concentration must be applicable to passage times in the 5*μ*m pores as well, it appears that the filtration time in the 5*μ*m pores is dominated by the time required for the RBCs to deform enough to gain entry into the channel.

### Estimation of average filtration time

The basic idea is to determine the average time required by the RBCs to deform enough to be able to pass through a channel that has a diameter smaller than that of the RBC and to then correlate this with the RBC CMS. To do this, one has to be able to determine an average filtration time per RBC (*T*) from the N* values determined from the flow experiment. We obtain the average filtration time in seconds using the expression *T* = 60/N*. Note that this is indeed an average time required for deformation and passage of an RBC through a channel measured from a large throughput process (the filtration time is averaged over at least 500 million RBCs). All variations arising from changes in velocity of the suspension fluid at the pore entry because of radial location of a pore or approach time of individual RBCs which might entail a simultaneous process of passage / deformation of two different RBCs with respect to the same channel are averaged over. The values thus obtained are tabulated in Table 2. This time would be the sum of the average time taken by the RBC to deform into a paraboloid of suitable radii (the deformation time, (*T*_*d*_)) and the average time taken to travel through the channel length to exit the filter paper at the outlet stage (the passage time, (*T*_*p*_)).

**Table 2:** Filtration time (*T*), passage velocity (*U*) and deformation time (*T*_*d*_) for RBC flow through 8*μm* and 5*μm* diameter channels for various concentrations of BSA used. Also tabulated is *β*, the translational drag modification factor, for various bRBCs.

b(x)RBC	8*μ*m		*β*	5*μ*m		
	T (ms)	U (mm/s)		T (ms)	U (mm/s)	*T*_*d*_ (ms)
0.50	6.94	1.44 ± 0.03	2.02 ± 0.03	19.60	1.00 ± 0.02	7.60 ± 0.40
0.70	6.75	1.48 ± 0.11	1.96 ± 0.15	22.99	1.02 ± 0.17	11.32 ± 2.00
1.00	6.47	1.54 ± 0.01	1.88 ± 0.02	29.41	1.07 ± 0.02	18.20 ± 0.30
1.18	6.34	1.57 ± 0.11	1.84 ± 0.13	32.51	1.09 ± 0.14	21.56 ± 1.50
1.35	6.02	1.65 ± 0.03	1.75 ± 0.03	36.44	1.15 ± 0.03	26.03 ± 0.40

### Estimation of RBC deformation time (*T*_*d*5*μm*_) in 5*μ*m pore diameter channels

For the case of the 8*μ*m diameter channel, one can assume that the RBCs travel through without undergoing any major deformation as the average human RBC diameter is ≈ 8*μ*m. Therefore, the average filtration time (*T*_8*μm*_) will be the passage time itself (*T*_*p*8*μm*_), as the deformation time *T*_*d*8*μm*_ will be zero and we can easily obtain the passage velocity of RBCs in the 8*μ*m diameter channel by simply dividing the filter paper thickness with *T*_8*μm*_. The variation of passage velocity with BSA concentration for the 8*μ*m diameter channel is shown in Fig 3c. It can be clearly seen that the passage velocities increase with increase in BSA concentration. Although, the velocities increase only marginally in the concentration range we have studied, if the channel is long enough the change in passage times could be significant. It could then be possible to study the effect of altered CMS, reflected in an altered translational drag, by merely studying the rate of passage of RBCs through channels with diameter larger than that of the RBC completely circumventing the need to analyse the more difficult case of RBC deformation and passage in narrower channels.

We obtain a theoretical expression for passage velocity of RBCs in narrow channels (*U*) using an equation of motion of RBCs in fluid flow where the forces acting on the RBC are the drag force and force due to kinematic pressure of the fluid (Eq 3). We use an expression for drag force (Eq S1) as given in [39] further incorporating a translational drag modification factor *β* that appears due to the altered CMS of the RBCs (see S1 File).

$$\begin{array}{c} U=\frac{1}{K_{U}\beta }\left(K_{V}V-\frac{\rho V^{2}a^{2}}{12\eta b}\right) \end{array}$$ (3)

where *V* is the velocity of the fluid, a and *b* are the radii of the channel and the RBC in its current shape respectively while *ρ* and *η* are the density and viscosity of the fluid respectively. *K*_*U*_ and *K*_*V*_ are drag dependent factors, given by Eq S2 and Eq S3 (see S1 File). Using this equation and the experimental estimates of the passage velocity of bRBCs in the 8*μ*m diameter channel, we find how *β* varies with the concentration of BSA used for treating the RBCs. The variation of *β* with BSA concentration is shown in Table 2.

Now, as *β* arises from the CMS of RBCs, it will remain unchanged for flow through the 5*μ*m diameter channel as well. Once more, using Eq 3 and the corresponding *β* values, we find a theoretical estimate for the passage velocities of bRBCs in 5*μ*m channel. Note that the drag force will be different for the case of the two channels as the shape of the RBCs and the flow conditions are different. Dividing the thickness of the 5*μ*m pore diameter filter paper with this theoretically determined passage velocity will yield *T*_*p*5*μm*_. The difference between *T*_5*μm*_ and *T*_*p*5*μm*_ gives us *T*_*d*5*μm*_. The *T*_*d*5*μm*_ values determined from the measured *T*_5*μm*_ values are shown in Table 2. From the values tabulated it can be seen that the deformation times of bRBCs in the 5*μ*m diameter channel increase with increase in BSA concentration in agreement with our argument that bRBCs are indeed rendered stiffer with high concentrations of BSA.

### Theoretical estimate of energies required for RBC deformation in narrow channels

We describe here a simple theoretical model of the deformation an RBC undergoes to enable passage through a channel with a diameter smaller than it’s own. Given the axial symmetry of the situation when an RBC is required to deform in order to gain entry into a narrow channel, we assume that the deformed RBC shape will also conform to this symmetry. We thereby assume the simplest possible symmetric shape of a paraboloid for it. Further, we impose a constraint that the surface area of the RBC remains constant throughout the entire process of deformation as the energy required for membrane surface area expansion is large when compared to the membrane bending energy of RBCs [2]. With this constraint in place, we estimate the height of the paraboloid, *h*, that will enable its passage into a channel of given radius (*a*) such that the deformed RBC radius is 0.25*μ*m less than *a*. Using *h*, we determine the final curvature the RBC surface has to acquire. The deformation energy (*ε*_*def*_) is then the energy required to effect the required curvature change of the RBC membrane.

**Fig 4:**
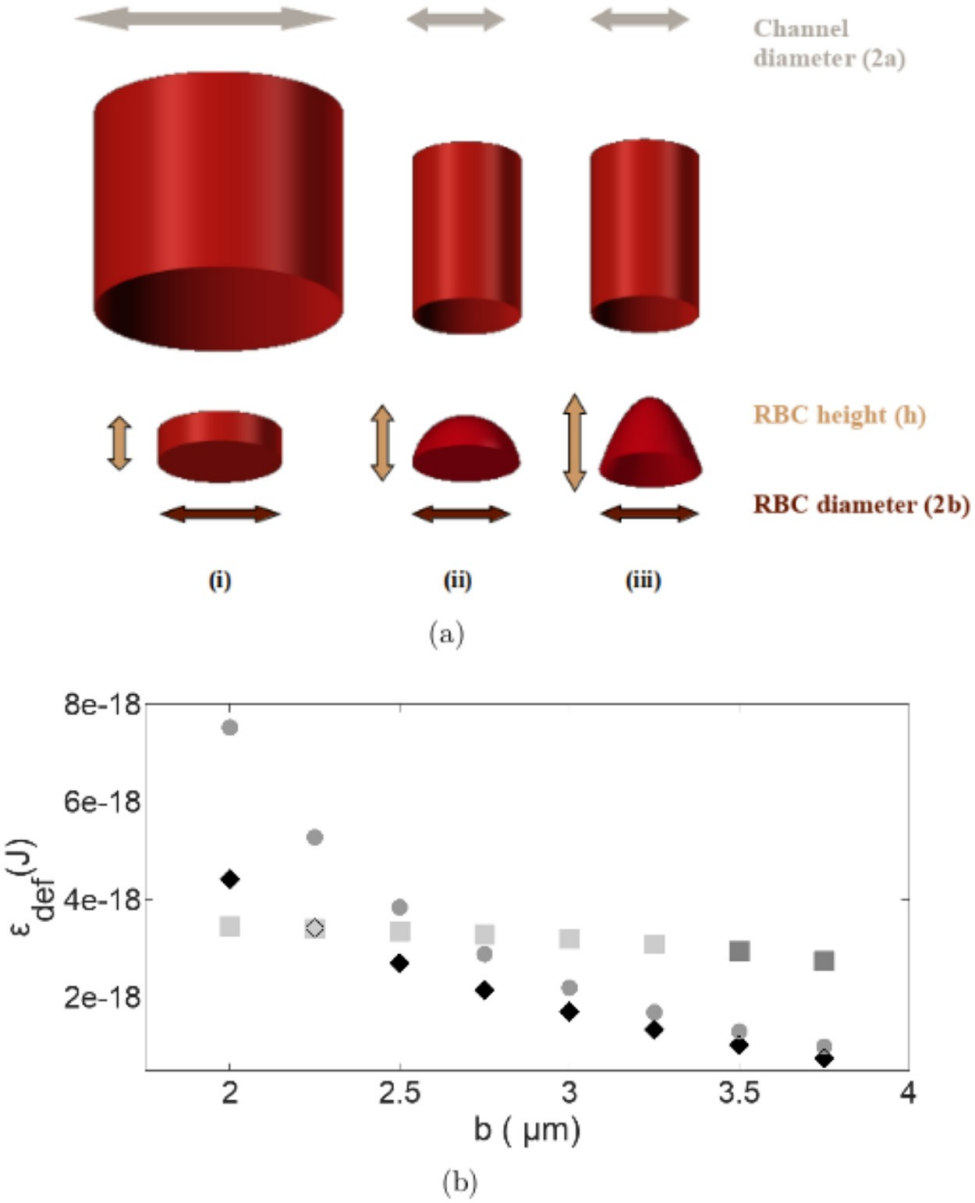
(a) Geometrical representation of RBC shape when in a channel with (i) diameter larger than that of the RBC (ii) diameter smaller than that of the RBC into a spherical cap (iii) deformation into a paraboloid. (b) Variation of deformation energies (theoretical) required for an RBC to deform into a (i) spherical cap (rectangles) assuming a constraint of constant surface area (light coloured rectangles represent the unphysical situation where the height of the spherical cap is greater than the radius of the hemisphere). (ii) paraboloid (black diamonds) assuming a constraint of constant surface area (used in our analysis) (iii) paraboloid (grey circles) assuming a constraint of constant volume.

It is reported in literature that RBCs deform maintaining both constant surface area and volume [40, 41]. We have calculated the *h* commensurate with a constraint of constant volume and find that it is consistently higher for all channel radii (*a*) when compared to the case of the constraint of constant surface area. This will imply a higher final curvature for the deformed RBC which will in turn imply higher deformation energy. In our analysis, we use the constraint of constant surface area as it is more energetically favourable within the limits of our model for the deformation process. We simplify further, by assuming the undeformed RBC to be a cylindrical disk with a diameter of 7.5*μ*m and height 2.022*μ*m (Fig 4a) yielding a surface area identical to that of the undeformed RBC. The energy required to change the curvature of an RBC is then given by the Helfrich elastic energy model [42]

$$\begin{array}{c} \varepsilon_{def}=\int \frac{1}{2}E_{b}{\left(2H-c_{0}\right)}^{2}dA \end{array}$$ (4)

where *E*_*b*_ is the bending modulus of the RBC membrane while *H* and *c*_*o*_ are the local mean curvature and spontaneous curvature of RBC respectively with *H* being defined as

$$\begin{array}{c} H=\frac{c_{1}+c_{2}}{2} \end{array}$$ (5)

with *c*_1_ and *c*_2_ being the principal curvatures at a given point on the surface. The integration is carried out over the RBC membrane surface area. This energy has to be computed for each major surface that is deforming. When the RBC deforms into a paraboloid we assume that the heights of the inner and outer parabolic caps are the same for the sake of simplicity (Fig 4a) and compute the height of the cap *h*. Knowing *h*, one can calculate the radius of curvature and thereby the curvature *H* of the deformed RBC. The energy involved in this deformation can be estimated using Eq 4. The theoretical deformation energy as a function of different radii of the paraboloid has been plotted in Fig 4b using the constraint of constant surface area. The deformation energies required when using a constraint of constant volume as well the energies required for RBC deformation into a spherical cap with a constraint of constant surface area have also been shown in Fig 4b for purposes of comparison.

### Theoretical estimate of *T*_*d*_

The energy required by an RBC to deform into paraboloid is provided by the blood flow. In our model, we consider the passage of a single RBC at a time through the narrow channel and not a bunch of stacked RBCs in the form of a rouleaux as is observed in blood flow in a normal human being. The condition required by our model can be achieved at low hematocrit levels where the effect of other RBCs on the passage of a particular RBC through the narrow channel can be ignored.

If we calculate the energy incident per unit time at the channel entrance per unit RBC surface area, we can find out how the energy builds up with time when an RBC blocks the entrance to a narrow channel. Energy incident at the RBC surface per unit time (*P*_*inc*_) is given by

$$\begin{array}{c} P_{inc}=KQ \end{array}$$ (6)

where *K* is the dynamic pressure *i.e*, kinetic energy carried by fluid per unit volume. The total energy per unit time available at the cross sectional area of an RBC that is stationary at the entrance of a narrow channel can be obtained as

$$\begin{array}{c} P_{inc}=\frac{1}{8}\pi \rho v_{max}^{3}a^{2} \end{array}$$ (7)

where *v*_*max*_ is the fluid velocity at a location on the axial line of the narrow channel where the deformation of the RBC takes place.

The deformation time *T*_*d*_ can be then written as

$$\begin{array}{c} T_{d}=\frac{\varepsilon_{def}}{P_{inc}} \end{array}$$ (8)

Thus it is evident that an estimate of *T*_*d*_ can yield the membrane bending modulus if the *P*_*inc*_ is known. The variable factor involved in determining *P*_*inc*_ is *v*_*max*_. Determining the value of *v*_*max*_ is non trivial as the fluid flow contracts at the entrance of the narrow channel. *v*_*max*_ would depend on the distance from the channel entry and its value would lie somewhere between the fluid velocity value set by the syringe pump and the fully developed velocity well within the narrow channels as determined from an application of Bernoulli‘s principle. A simulation of such a flow in the case of the 5*μ*m channel diameter filter paper is shown in S1 File. One way of determining *v*_*max*_ would be to use the deformation time of some bRBC whose bending modulus is already estimated by an alternate measurement technique and then use this value of *v*_*max*_ for all other types of bRBCs. To do this and to also assess the change in RBC CMS with BSA treatment using an alternate method, we study their reorientation dynamics in an OT.

### bRBC reorientation in optical tweezers to assess their CMS

RBC filtration results suggest that BSA treatment elevates the CMS of RBCs, the extent of elevation being dependent on the exact concentration of BSA used. We need a gauge of the extent to which the RBC CMS is affected in comparison to nRBCs. Therefore, we have carried out RBC reorientation experiments as described by Parthasarathi *et al* in [14]. We measure the variation of reorientation times of various bRBCs with laser power as outlined in methods section. Note that at the concentrations of BSA being used there is no change in either the viscosity [43] or the refractive index of the surrounding medium [44]. Additionally as discussed earlier, the size of the RBCs is unaffected by BSA treatment (Table 1). Therefore, the changes observed in the reorientation times of various bRBCs can be attributed to a CMS change rather than a change in RBC size or change in the properties of the suspension medium.

**Fig 5:**
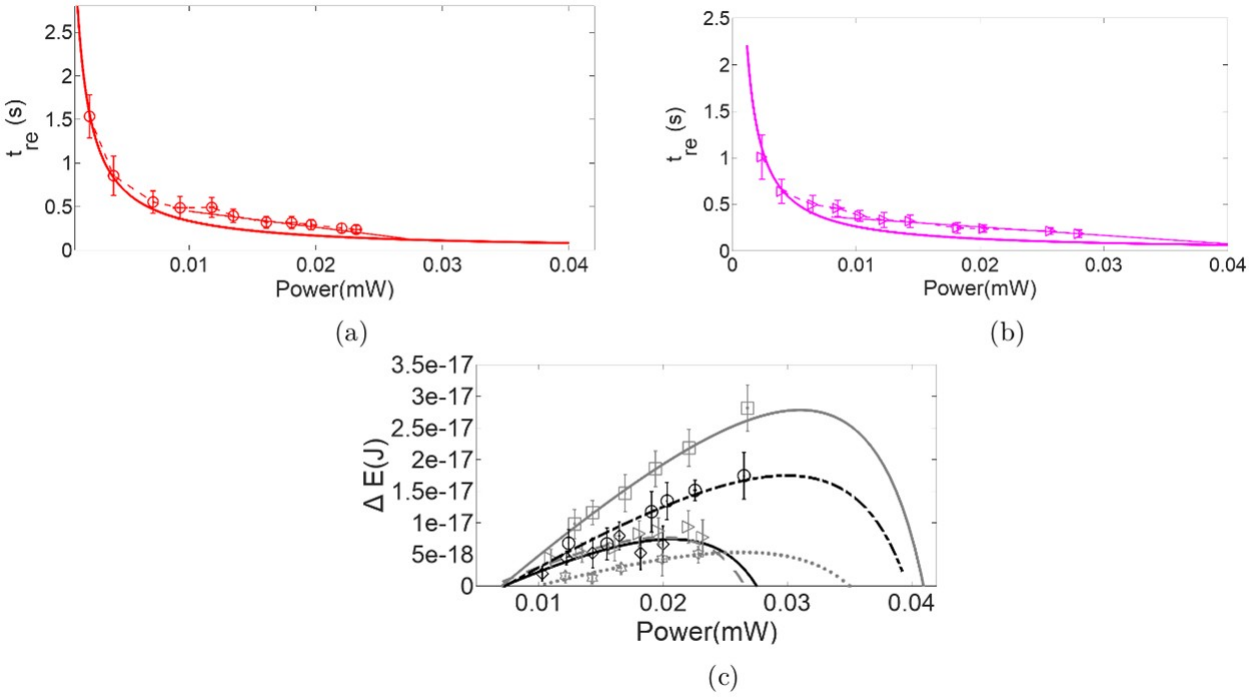
(a) and (b): Variation of time of reorientation (*t*_*re*_) as a function of laser power at the sample plane (*P*) for (a) control (circles) and (b) b(1.35)RBCs (triangles) respectively. The solid lines represent the behaviour of a perfectly rigid body with identical rotational drag (extracted from the behaviour of the RBCs at the lowest laser powers used). Note that *t*_*re*_ varies inversely as *P*. (c): Variation of Δ*E* with laser power at the sample plane for control (triangles and dashed grey line), b(0.50)RBC (diamonds and solid black line), b(1.00)RBC (circles and dash dot black line), and b(1.75)RBC (squares and solid grey line) showing a maximum value of ΔE that is equal to or greater than that for the control respectively. While the symbols represent the values extracted from the experimentally measured values, the lines represent a theoretical variation that is also extrapolated to power ranges not amenable to experiment. Note: b(0.10)RBC (hexagons and dotted grey line) is shown only for comparison.

Variation of the time of reorientation, *t*_*re*_, as a function of laser power at the sample plane (*P*) is shown in Fig 5a and 5b for the control and b(1.35)RBC respectively. While we follow the analysis technique described in detail in Parthasarthi *et al* [14], a brief outline of the method followed is provided here. As the laser power is increased, less time is required for the reorientation process, resulting in smaller values of *t*_*re*_. If we were to assume the RBC to be a perfect rigid body, the variation in *t*_*re*_ with laser power would follow a 1/*P* relation. Assuming that the behaviour at the lowest powers is most like that of a rigid body, we extract from the variation of *t*_*re*_ at the lowest laser powers used, a term (*α*) that modifies the rotational drag of a perfectly rigid cylindrical disc to that of a perfectly rigid RBC shaped object (see S1 File). We then use this to predict the behaviour of a perfect rigid body that has dimensions and drag coefficients identical to the RBC at all laser powers. The solid lines shown in the Fig 5a and 5b depict this. We find that the variation of *t*_*re*_ measured for the RBC with *P* deviates from that predicted for the perfect rigid body beyond a certain value of *P* (*P*_*d*_). Beyond *P*_*d*_, the RBC is found to require more time for the reorientation process than that predicted for the rigid body. We fit a straight line through the values of *t*_*re*_ beyond *P*_*d*_ and use this to predict the excess time required by the RBC compared to its perfectly rigid counterpart. We then use this excess time to estimate the excess energy Δ*E*, which is the amount of energy utilized in the RBC reorientation process that is in excess of that required for a rigid body with dimensions and rotational drag coefficient identical to the undeformed RBC. Variation of Δ*E* as a function of *P* is shown in Fig 5c for RBCs treated with different concentrations of BSA.

To enable a comparison between the CMS of the different RBCs, the rotational drag modification term, *α*, is factored out from Δ*E* (see S1 File). It can be seen in each of the cases that Δ*E* goes through a maximum value, which we define as Δ*E*_*max*_. This value corresponds to the maximum energy that is spent on membrane reconformation during a reorientation process and scales with the RBC CMS [14]. The variation of Δ*E* with *P* has been shown in Fig 5c for the control, b(0.50)RBC, which mimics the control and b(1.00)RBC and b(1.75)RBC, both of which have a Δ*E*_*max*_ larger than that of the control. The values of Δ*E*_*max*_ for the control as well as the b(x)RBCs are reported in Table 3.

**Table 3:** Comparison of Δ*E*_*max*_ obtained in the current study along with that reported in [14].

Data set (current)	ΔE_max_(10^−18^J)	Data set (reported in [14])	ΔE_max_(10^−18^J)
*b*(0.50)*RBC*	7.41±1.30	Control	8.0 ± 1.85
*Control*(*untreated*)	7.8±1.50		
*b*(0.70)*RBC*	12.97±2.05	hRBC	18.7 ± 2.16
*b*(1.00)*RBC*	17.50±1.80		
*b*(1.35)*RBC*	23.34±1.34		
*b*(1.75)*RBC*	25.88±2.10		

**Fig 6:**
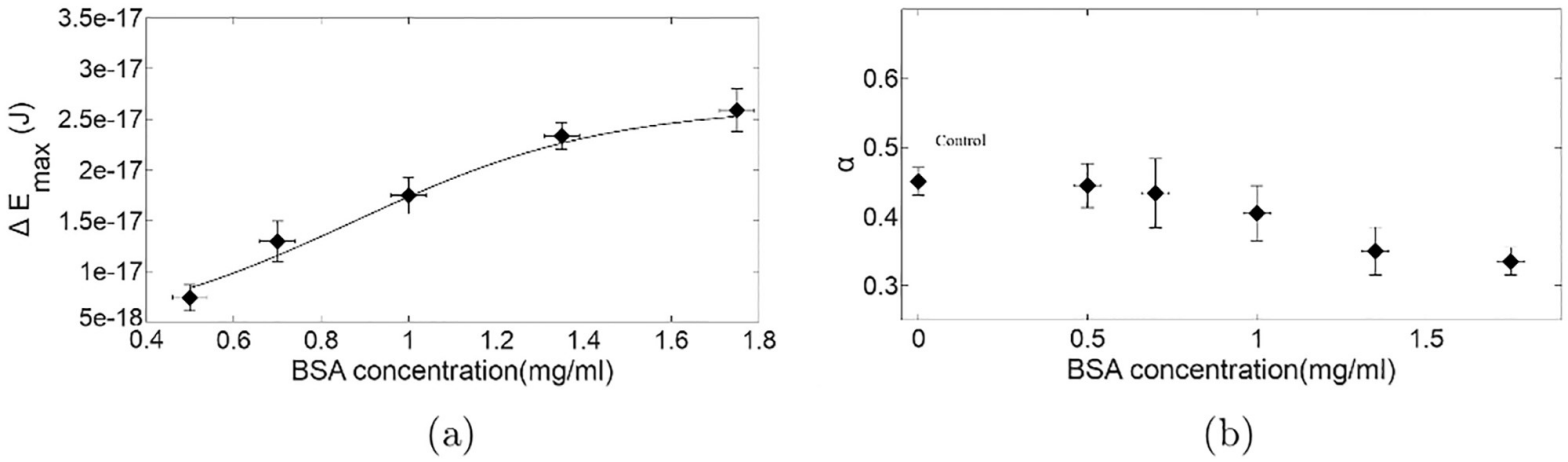
(a) Variation of Δ*E*_*max*_ with the concentration of BSA used for treating the RBCs. (b) Variation of the rotational drag modification term, *α*, with the concentration of BSA used for treating the RBCs. Note that the *α* value for the untreated RBCs (control) is also mimicked by treatment with a BSA concentration of 0.50mg/ml.

The Δ*E*_*max*_ for the control is found to be within reasonable limits of that determined for normal RBCs [14]. It is evident from Table 3, that RBCs in suspensions with concentrations of BSA greater than 0.50mg/ml have a Δ*E*_*max*_ that is greater than that obtained for the control and comparable to the values obtained for RBCs extracted from individuals with an established condition of hyperglycemia (hRBC) reported in [14]. Further, Δ*E*_*max*_ is found to increase with the increase in BSA concentration beyond 0.50mg/ml (see Table 3). The variation of Δ*E*_*max*_ with BSA concentration is shown in Fig 6a. Thus it appears that BSA added to a low hematocrit RBC suspension at concentrations higher than 0.50mg/ml alters the mechanical properties of the RBCs by elevating the CMS. This is in agreement with the micropore filtration experiments where *T*_*d*5*μm*_ is found to increase with increase in BSA concentration (see Table 2) thereby endorsing our assumption of the effect of BSA treatment on the RBC CMS. Note, it is possible that this enhanced CMS counters the crenation that invariably takes place at such low hematocrit when BSA is absent [17, 32].

The other interesting observation made while analysing the results is the variation of *α* with BSA concentration (Fig 6b). Note firstly that all *α* values are lower than 1. This goes to show that RBCs invariably have a rotational drag that is lower than that of a cylindrical disc with a diameter identical to that of an RBC. Further, we find that the value of *α* decreases with the increase in BSA concentration (Fig 6b). It is found that b(0.50)RBCs have an *α* value close to that of the control and bRBCs with concentrations greater than 0.50mg/ml have rotational drag consistently lower than the control. This correlates with the result seen in the micropore filtration experiment where the translational drag modification factor *β* was also found to decrease with increase in BSA concentration (see Table 2).

### Extraction of membrane bending modulus (*E*_*b*_) of bRBCs from the *T*_*d*5*μm*_ values

**Fig 7:**
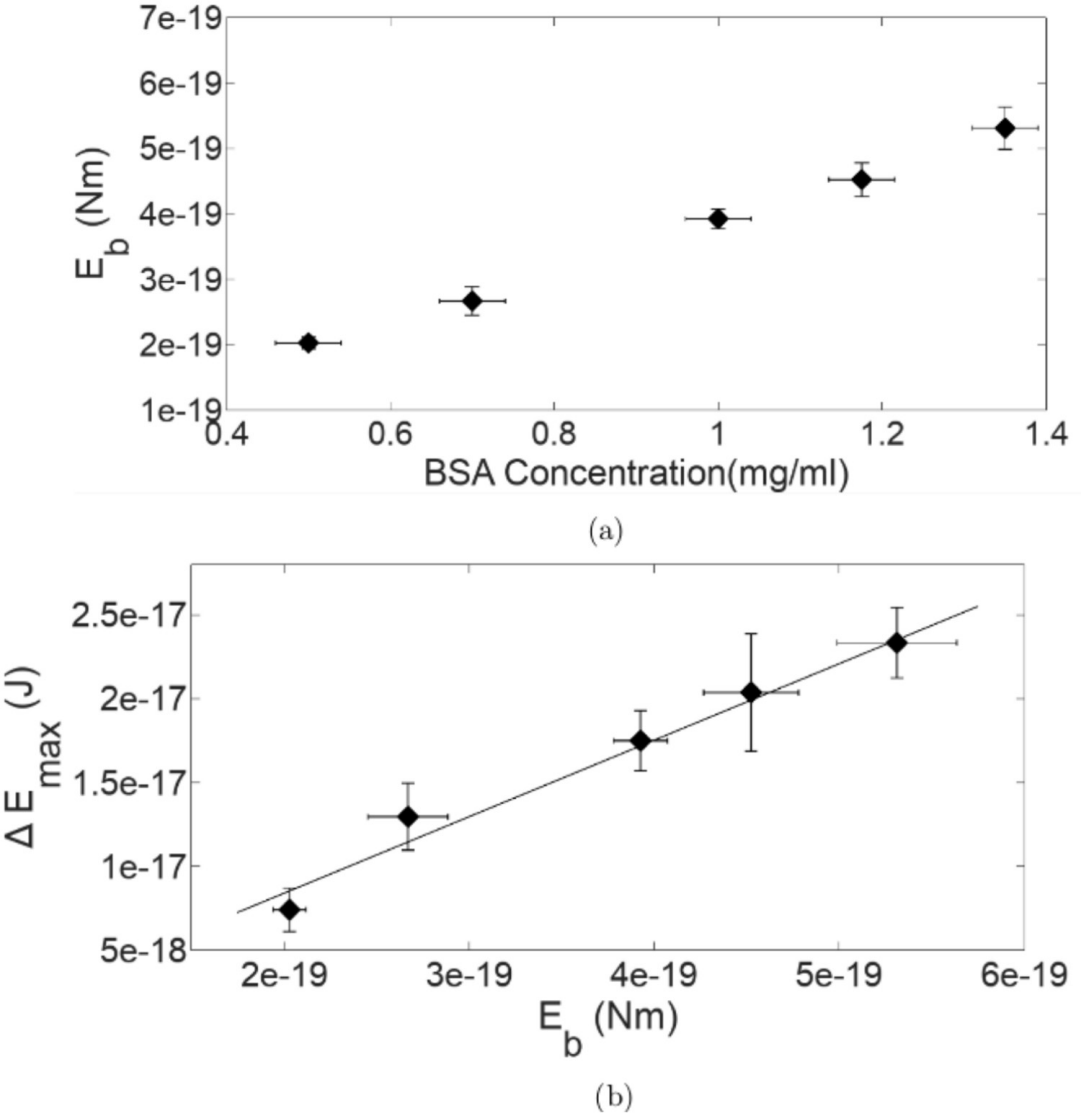
(a) RBC membrane bending modulus *E*_*b*_ of various b(RBCs) studied (b) Correlation of RBC membrane bending modulus *E*_*b*_ estimated from RBC flow through narrow channels (*E*_*b*_) with Δ*E*_*max*_. The solid line shown is the straight line fit to the data [Note: The Δ*E*_*max*_ value for b(1.18)RBC, whose reorientation behaviour was not actually studied, is obtained by fitting a sigmoid curve to the data in Fig 6a].

It can be noted that the reorientation times and Δ*E*_*max*_ obtained for b(0.5)RBCs are identical to that of normal RBCs. Thereby, we assume that the CMS of b(0.5)RBC is identical to that of nRBC and assign a value of 2*X*10^−19^ J reported for normal RBCs [45], to *E*_*b*_ for b(0.5)RBC. Then using Eq 8 we obtain a value of 4.27mm/s for *v*_*max*_. This velocity will remain the same for the case of all bRBCs as it depends on the fluid flow conditions which are maintained identical throughout. From the simulation of flow conditions in filtration through a 5*μ*m channel diameter filter paper (see S1 File), it can be seen that such a value for the fluid velocity is indeed acceptable [46]. An estimate of *E*_*b*_ of other bRBCs using *T*_*d*5*μm*_ can now be obtained and is plotted as a function of BSA concentration in Fig 7a. We see that the values of *E*_*b*_ increase with increase in BSA concentration. Note that the bending modulus extracted for the bRBCs here, is an “effective” bending modulus that is estimated by averaging over the filtration behaviour of more than 500 million RBCs and all the possible variations in minute details that can possibly entail.

Further, when the values of *E*_*b*_ obtained are plotted against the Δ*E*_*max*_ values obtained from the studies on RBC reorientation in an OT, a linear correlation (Fig 7b) is seen. This clearly indicates that the stress regime that the RBC is subjected to in a reorientation process in an OT is the same as what an RBC, at flow rates encountered invivo, is subjected to when passing through narrow channels that are of dimensions similar to capillaries in the human vasculature. This justifies our use of RBC reorientation dynamics in an OT, over other experimental techniques, for CMS assessment when RBC flow in narrow channels is concerned. The extraction of bending modulus of bRBCs from the micropore filtration experiments using the theoretical models is outlined as a condensed scheme in S1 File.

## Conclusions

We have conclusively shown from our flow experiments that treating RBCs with BSA at suitable concentrations renders the RBC cell membrane stiffer and that micro circulation time of such CMS elevated RBCs is clearly affected. The elevated CMS can mimic the RBC CMS observed for individuals with a medical condition of hyperglycemia. This has been established from reorientation dynamics of BSA treated RBCs in an OT and comparison with results of similar experiments that were carried out earlier on hRBCs. The fact that RBC CMS elevated to an extent seen in hRBCs has a definite effect on the RBC flow rate in micro channels calls for an investigation into the medical implications of such a find. While the flow experiments reported here have been carried out at a low hematocrit level for the reasons that have been outlined, we expect that a similar trend will be seen at normal hematocrit levels as well. However, the extent of change will not be a linear extrapolation of the situation seen with individual RBCs. This is because flow processes at higher hematocrit levels will involve deformation and passage of stacks or rouleaux of RBCs. Their deformation and passage characteristics are unlikely to be linearly related to the parameters for single RBCs.

Artificially changing the CMS of RBCs extracted from an individual with no chronic medical conditions to different extents and using those in a study present distinct advantages as it could isolate the consequences of a CMS change alone. It is, however, physically impossible that increasing BSA concentrations will increase the RBC CMS indefinitely and we can expect a saturation effect at higher concentrations of BSA. This has, however, not been observed by us in the BSA concentration range within which we have carried out this study.

We have used a highly simplified model for RBC deformation to estimate the membrane bending modulus of bRBCs from the RBC flow experiments. We have also outlined a possibility of estimating the RBC CMS from the velocity of passage of the RBCs in broader channels using the translational drag modification factor. The RBC flow through narrow channels can serve as a cost effective method to gauge the RBC CMS. Given that RBCs with CMS affected to the extent seen in individuals with hyperglycemia show a marked difference in flow experiments, it could be used as a basis for a new diagnostic technique that may enable earlier detection of an underlying medical condition where RBC CMS is known to be affected.

## Supporting information

S1 File Contains appendices and Eqs S1-S7. **S1 Appendix:** Optical tweezers set up. **S2 Appendix:** Drag acting on RBC in narrow channels. **S3 Appendix:** Flow contraction from active area to 5*μ*m pore. **S4 Appendix:** Extraction of modified rotational drag factor (*α*). **S5 Appendix:** Extraction of bending modulus of bRBCs from their flow rate in narrow channels: A condensed scheme.
(PDF)

S1 Fig Optical tweezers set up used for carrying out RBC reorientation experiment. (TIF)

S2 Fig Simulated flow velocity profile of a flow contraction from a larger channel with a fluid velocity of 0.64mm/s (*A*_*active*_ = 19.63*μm*^2^) to a 5*μ*m pore diameter channel using OpenFOAM. The flow is along the axis of the channel (Z axis). The pore entrance is located at *z* = 15*μ*m.
(TIF)

S1 Image (ZIP)

S2 Image (ZIP)

S1 Review (ZIP)

S1 Video (ZIP)

S2 Video (ZIP)

S3 Video (ZIP)
