# Correction: Estimation of membrane bending modulus of stiffness tuned human red blood cells from micropore filtration studies

**DOI:** 10.1371/journal.pone.0228125

**Published:** 2020-01-16

**Authors:** Rekha Selvan, Praveen Parthasarathi, Shruthi S. Iyengar, Sharath Ananthamurthy, Sarbari Bhattacharya

There are errors in the Author Contributions. The correct contributions are:

**Conceptualization:** Rekha Selvan, Sharath Ananthamurthy, Sarbari Bhattacharya

**Data curation:** Rekha Selvan, Shruthi S. Iyengar

**Formal analysis:** Rekha Selvan, Sarbari Bhattacharya

**Funding acquisition:** Sharath Ananthamurthy

**Investigation:** Rekha Selvan, Sarbari Bhattacharya

**Methodology:** Rekha Selvan, Praveen Parthasarathi, Sarbari Bhattacharya

**Project administration:** Sarbari Bhattacharya

**Resources:** Sharath Ananthamurthy

**Software:** Rekha Selvan, Praveen Parthasarathi

**Supervision:** Sharath Ananthamurthy, Sarbari Bhattacharya

**Validation:** Rekha Selvan, Praveen Parthasarathi, Shruthi S. Iyengar

**Visualization:** Rekha Selvan, Sarbari Bhattacharya

**Writing – original draft:** Rekha Selvan, Sarbari Bhattacharya

**Writing – review & editing:** Rekha Selvan, Shruthi S. Iyengar, Sharath Ananthamurthy, Sarbari Bhattacharya
