# Supplementary material for: Estimation of membrane bending modulus of stiffness tuned human red blood cells from micropore filtration studies
Source: PLoS One. 2019 Dec 31;14(12):e0226640. doi: 10.1371/journal.pone.0226640 (PMC6938315; doi:10.1371/journal.pone.0226640)
Supplement: S1 File — S1 Appendix: Optical tweezers set up. S2 Appendix: Drag acting on RBC in narrow channels. S3 Appendix: Flow contraction from active area to 5μm pore. S4 Appendix: Extraction of modified rotational drag factor (α). S5 Appendix: Extraction of bending modulus of bRBCs from their flow rate in narrow channels: A condensed scheme. (PDF) [file pone.0226640.s001.pdf]

Estimation of membrane bending modulus of stiffness tuned human red blood cells from micropore filtration studies

Supporting information

S1 Appendix Optical tweezers set up The optical tweezers set up used for measuring reorientation times is shown in S1 Fig. Laser of wavelength 1064 nm(Ventus IR) with an oil immersion objective(100X zoom and NA=1.4) was used for tweezing purposes. The microscope was used in inverted configuration with XYZ manual stage control. The samples were imaged and recorded using a CCD camera.

S1 Fig. Optical Tweezers set up used for carrying out RBC reorientation experiment

S2 Appendix Drag acting on RBC in narrow channels

To find the drag acting on the RBC, we use results from [1] with inclusion of an RBC CMS dependent translational drag factor β. Lubrication theory is used to obtain the drag acting on axisymmetric particles (RBCs, in our case) of radius b in a channel of comparable radius a (b_0 = b/a $\geq$ 0.9). This is a scenario, where both the fluid and RBCs are in motion. The drag coefficients K_U and K_V are obtained for the two cases when (i) the RBC is in motion but the fluid is stationary and (ii) the fluid is moving while RBC is stationary. The total drag acting on the RBC is then given by

Eq S1

$$D = 6\pi\eta b(-K_U U\beta + K_V V)$$

where K_U and K_V are computed using the integrals,

Eq S2

$$K_U = \frac{-1}{3b_o}\int_{-z_o}^{+z_o} \frac{1 + R_o^2(z)}{1 - R_o^2(z) + (1 + R_o^2(z))lnR_o(z)}dz$$

Eq S3

$$K_V = \frac{-1}{3b_o}\int_{-z_o}^{+z_o} \frac{1}{1 - R_o^2(z) + (1 + R_o^2(z))lnR_o(z)}dz$$

where z is along the axis of the channel and R_o(z) is the shape factor describing the shape of the RBC. The integral is evaluated over the RBC extent along the z-axis from $-z_o$ to $+z_o$. In the case of the 8μm channel, we assume the RBCs to be oblate spheroids of surface area $136\mu m^2$. We assume the major axis of the ellipse to be 7.5μm ($2b$) and compute the minor axis ($2z_o$) to match the surface area of RBC.

Eq S4

$$R_o(z) = b_o(1 - (\frac{z}{a})^2)^{0.5}$$

The values obtained for K_U and K_V are 3908 and 2116 respectively. In the case of the 5μm channel, RBCs would be deformed into paraboloids. The height of the paraboloid to fit a 5μm channel was found to be 6.16μm using the model described in the results, analysis and discussions section. The shape factor used here is

Eq S5

$$Ro(z) = b_o(1 - (\frac{z}{h})^2)$$

The drag coefficients K_U and K_V were obtained by evaluating integrals given in Eq S2 and Eq S3 over the height of the paraboloid and found to be 1290 and 690 respectively.

S3 Appendix Flow contraction from active area to 5μm pore We have used an active area of $12.56mm^2$ to find N^* in our analysis. There will be a finite spread in the flow of RBC suspension around the active area while flowing from the inlet of the filter holder on to the filter paper (due to the separation between the two). Hence, we consider an excess area of ring of 0.5 mm radius around the active area. Therefore, the flow converges from an area of $19.63mm^2$ to an effective reduced cross sectional area given by the product of N_{pore} and cross section of pore of diameter 5μm. Pore density (N_{pore}) was estimated from the photographs of the filter papers.

S2 Fig Simulated flow velocity profile of a flow contraction from a larger channel with a fluid velocity of 0.64mm/s ($A_{active} = 19.63\mu m^2$) to a 5$\mu$m pore diameter channel using OpenFOAM. The flow is along the axis of the channel (Z axis). The pore entrance is located at z=15μm.

According to Bernoulli's principle, with convergent flows, the velocity increases in the narrower channels to satisfy the continuity equation. We find that the resulting steady state peak velocity is 7.56 mm/s. Of course, this transition from the velocity in the larger channel to that in the narrower one is gradual and will happen over a finite axial distance from the pore entrance. Also, owing to the extended size of the RBC, the deformation process might take place anywhere between 1.5μm to 8μm from the entrance of a 5μm pore. Using Open Field Operations And Manipulations (OpenFOAM) – a Computational Fluid Dynamics software, we have obtained a velocity profile for such an abrupt contraction and a plot for this is shown in S2 Fig. Thus a velocity of 4.27mm/s (about 30% to 65 % of the fully developed flow velocity in the narrow channel [2] is a reasonable one to use for the computation of the energy incident per unit time on the RBC surface presented perpendicular to the flow direction.

S4 Appendix Extraction of modified rotational drag factor (α) The theoretical variation of t_{re} with P for a rigid circular disc with radius identical to that of an RBC was obtained in [3] by solving the rotational Langevin equation. However, a circular disc is a poor approximation of the RBC shape and thereby requires a factor α which can modify the Perrin coefficient 32/3, appropriate for a circular disc such that α(32/3) becomes the suitable Perrin coefficient for a perfectly rigid RBC shaped object. Then the appropriate Langevin equation then becomes

Eq S6

$$I\ddot{\theta} + \frac{32}{3}\alpha\eta r_o^3\dot{\theta} = \tau_{laser} + \tau_{thermal}$$

where I is the moment of inertia of the RBC about the axis of reorientation, $\dot{\theta}$ and $\ddot{\theta}$ are the first and second derivative of the angular displacement θ with respect to time, respectively, η the viscosity of the surrounding medium, r_o the radius of RBC and τ_{laser} and $\tau_{thermal}$, the laser and thermal generated torques acting on the RBC, respectively. The factor α is determined from the experimental variation of t_{re} with P for each type of bRBC at the lowest powers (first three data points). Note that the α values vary with the BSA concentration indicating that the rotational drag of the RBCs are themselves affected by BSA treatment. The expression for excess energy (ΔE) required

to rotate an RBC by 90 over and above that required for it's perfectly rigid counterpart is given by Eq S7 [3].

Eq S7

$$\Delta E(P) = \frac{\pi}{2}\frac{32}{3}\alpha\eta {r_o}^3 (\frac{\pi/2}{t_{re(theory)}} - \frac{\pi/2}{t_{re(experimental)}})$$

The excess energy ΔE is divided by α to enable comparisons across various bRBCs to study effect brought about by RBC CMS change alone.

S5 Appendix Extraction of bending modulus of bRBCs from their flow rate in narrow channels: A condensed scheme

- RBCs suspended in PBS with different BSA concentrations at hematocrit of 5% were passed through polycarbonate membrane filters that had pores of diameter (i) $5\mu m$ and (ii) $8\mu m$ at a constant flow rate of $400\mu l/min$. Four samples were collected sequentially at the output stage at intervals of 1 minute each. The average concentration of RBCs measured from these was taken as N_{out}. Using equation Eq 1, the average number of RBCs that flow out of each pore every minute is obtained.

$$N^* = \frac{N_{out}Q}{A_{active}N_{pore}} \quad (1)$$

  From N^*, the average filtration time (T) of a single RBC at each BSA concentration for each of the two pore diameters is obtained.

- In the case of the $8\mu m$ pore, RBCs do not undergo deformation and thereby, $T = T_p$ (passage time). Using this, the velocities of RBCs in the channel length (U) are obtained. The theoretical passage velocities of bRBCs in such pores are also obtained using lubrication theory by incorporating, additionally, a translational drag modification factor (β) to account for the effect of BSA treatment on the RBC translational drag. Since U of various bRBCs in $8\mu m$ pores are known, we use Eq 2 to obtain β.

$$U = \frac{1}{K_U\beta}(K_V V - \frac{\rho V^2 a^2}{12\eta b}) \quad (2)$$

- Since β does not depend on the flow properties of the channel, the same values of β are used for computing U of various bRBCs in the $5\mu m$ pores. Having obtained U, the passage times (T_p) in $5\mu m$ pore are obtained as the channel length (thickness of the filter paper) is known. T is the sum of deformation time (T_d) and T_p . The experimental deformation times for various bRBCs in the $5\mu m$ pores are obtained using the values of T and T_p.

- The theoretical expression for T_d for a $5\mu m$ pore is then estimated from the theoretical model set up by us using Eqs 3, 4 and 5.
  Theoretical estimate of energy required by an RBC to deform into a paraboloid

$$\varepsilon_{def} = \int \frac{1}{2}E_b(2H - c_0)^2 dA \quad (3)$$

  Energy incident per unit time at the surface of the RBC deforming at the entrance of pore

$$P_{inc} = \frac{1}{8}\pi\rho v_{max}^3 a^2 \quad (4)$$

Thus, the time of deformation becomes,

$$T_d = \frac{\varepsilon_{def}}{P_{inc}} \tag{5}$$

From reorientation experiments, we find that b(0.5)RBC mimics the characteristics of control RBCs. We thereby assign bending modulus value of the control to b(0.5)RBC and using Eq 5, we find the value of v_{max}, the fluid velocity at which the deformation takes place. We expect other bRBCs to also deform at the same velocity as the flow characteristics should be independent of the type of RBC being studied. So, using Eq 5, bending modulus values of all bRBCs are obtained.

References

1. Chen TC, Skalak R. Stokes flow in a cylindrical tube containing a line of spheroidal particles. Appl Sci Res. 1970;22:403.

2. Xia Y, Callaghan PT, Jeffrey KR. Imaging velocity profiles: flow through an abrupt contraction and expansion. AlChE J. 1992;38(9):1408.

3. Parthasarathi P, et al. Orientational dynamics of human red blood cells in an optical trap. J Biomed Opt. 2013;18(2):025001.

Essential raw data extracted from the images and videos recorded during the experiments

Mircopore filtration studies Typical image sequences used to obtain the output concentration of RBCs using hemocytometer have been attached (S1 and S2 image sequences). Raw data of average concentration of bRBCs (N_{out}) that was obtained after passing them through cyclopore filters of pores of diameters 5μm and 8 μm is tabulated below.

Data set	$\mathbf{N_{out}}$(cells/μl)	
	**5 μm pore**	**8 μm pore**
b(0.50)RBC	437710 ± 5432	337504 ± 4570
b(0.70)RBC	370672 ± 17690	346887 ± 27344
b(1.00)RBC	289721 ± 6527	360941 ± 3703
b(1.18)RBC	276944 ± 18406	369732 ± 26836
b(1.35)RBC	233765 ± 5845	388833 ± 7500

Optical tweezers based reorientation experiments Sample videos of RBCs reorientation under optical tweezers have been uploaded(see S1 to S3 videos). The protocol used to extract the same has been provided in the manuscript. The reorientation times (t_{re}) extracted from the videos for all bRBCs at all the laser powers is tabulated below.

**Data set**	**Laser power P(W)**	**Error(W)**	t_{re}**(s)**	**Error(s)**
Control	0.00215	7.81e-06	1.535	0.4461
	0.00405	3.27e-05	0.855	0.2256
	0.00717	8.08e-06	0.554	0.1268
	0.00933	7.72e-05	0.487	0.1298
	0.01180	1.50e-05	0.492	0.1129
	0.01376	1.04e-04	0.395	0.0769
	0.01611	2.61e-05	0.322	0.0667
	0.01811	6.98e-05	0.325	0.0701
	0.01964	1.02e-05	0.293	0.0577
	0.02203	3.07e-05	0.253	0.0533
	0.02310	7.75e-05	0.238	0.0454
	0.02459	3.23e-05	0.239	0.0615
	0.02843	1.94e-05	0.242	0.0553
b(0.50)RBC	0.00205	2.14e-05	1.464	0.3747
	0.00399	5.19e-06	0.886	0.1972
	0.00650	5.20e-05	0.597	0.1770
	0.00851	9.53e-06	0.522	0.0500
	0.01030	4.05e-05	0.395	0.0554
	0.01224	1.02e-05	0.409	0.0550
	0.01435	3.67e-05	0.350	0.0971
	0.01650	7.49e-05	0.359	0.0937
	0.01820	2.40e-05	0.250	0.0510
	0.02032	1.16e-05	0.320	0.1395
	0.02310	6.48e-05	0.250	0.0442
	0.02570	3.09e-05	0.250	0.0444
	0.02800	5.72e-05	0.241	0.0431
b(0.70)RBC	0.00208	3.20e-05	1.366	0.2260
	0.00428	1.50e-05	1.123	0.1902
	0.00660	8.33e-05	0.623	0.1530
	0.00882	4.85e-06	0.562	0.0765
	0.01130	6.76e-05	0.535	0.1073
	0.01434	2.05e-05	0.395	0.0937
	0.01680	3.74e-05	0.337	0.0712
	0.01985	1.04e-05	0.350	0.0843
	0.02275	6.25e-05	0.263	0.0416
	0.02372	5.92e-05	0.281	0.0655
	0.02605	2.21e-05	0.250	0.0001
	0.03030	1.16e-05	0.187	0.0360
b1.00)RBC	0.00202	3.50e-05	1.294	0.2549
	0.00468	1.77e-05	0.731	0.1069
	0.00692	2.87e-05	0.555	0.2263
	0.00993	3.20e-05	0.465	0.1130
	0.01242	1.66e-05	0.416	0.1169
	0.01550	2.81e-05	0.291	0.0625
	0.01909	6.48e-05	0.296	0.0888
	0.02034	2.56e-05	0.296	0.0786
	0.02258	9.60e-05	0.270	0.0322
	0.00265	3.27e-05	0.226	0.0572
	0.02828	1.97e-05	0.245	0.0538
	0.03015	6.75e-05	0.242	0.0847

**Data set**	**Laser power P(W)**	**Error(W)**	t_{re}**(s)**	**Error(s)**
b(1.35)RBC	0.00205	2.14e-05	1.137	0.2137
	0.00399	5.20e-06	0.687	0.1593
	0.00650	5.20e-05	0.500	0.0968
	0.00850	9.53e-06	0.461	0.0866
	0.01030	4.05e-05	0.380	0.0588
	0.01224	1.02e-05	0.336	0.0814
	0.01435	3.67e-05	0.321	0.0668
	0.01650	7.50e-06	0.325	0.0645
	0.01820	2.40e-05	0.250	0.0559
	0.02032	1.16e-05	0.243	0.0375
	0.02570	3.10e-06	0.215	0.0329
	0.02800	5.72e-06	0.187	0.0395
b(1.75)RBC	0.00206	1.44e-05	1.011	0.2474
	0.00402	5.37e-05	0.678	0.1148
	0.00784	1.52e-05	0.441	0.1123
	0.01295	5.26e-05	0.320	0.0557
	0.01435	1.86e-05	0.300	0.0385
	0.01695	5.25e-05	0.267	0.0492
	0.01940	1.75e-05	0.253	0.0421
	0.02210	4.25e-05	0.229	0.0351
	0.02680	4.14e-05	0.222	0.0344
b(0.1)RBC for comparison	0.00198	4.80e-05	2.048	0.4980
	0.00395	1.33e-05	1.193	0.3312
	0.00590	5.00e-05	0.513	0.1025
	0.00821	7.00e-06	0.508	0.0759
	0.01032	1.50e-05	0.518	0.1667
	0.01215	3.22e-05	0.437	0.0600
	0.01432	3.70e-05	0.343	0.0747
	0.01665	1.68e-05	0.343	0.0361
	0.01995	1.70e-05	0.312	0.0684
	0.02285	1.50e-05	0.277	0.0329
	0.02484	1.85e-05	0.281	0.0625
	0.02816	1.10e-05	0.291	0.0322
	0.02999	5.96e-05	0.216	0.0324
