# Supplementary figures and images for: Estimation of membrane bending modulus of stiffness tuned human red blood cells from micropore filtration studies
Source: PLoS One. 2019 Dec 31;14(12):e0226640. doi: 10.1371/journal.pone.0226640 (PMC6938315; doi:10.1371/journal.pone.0226640)

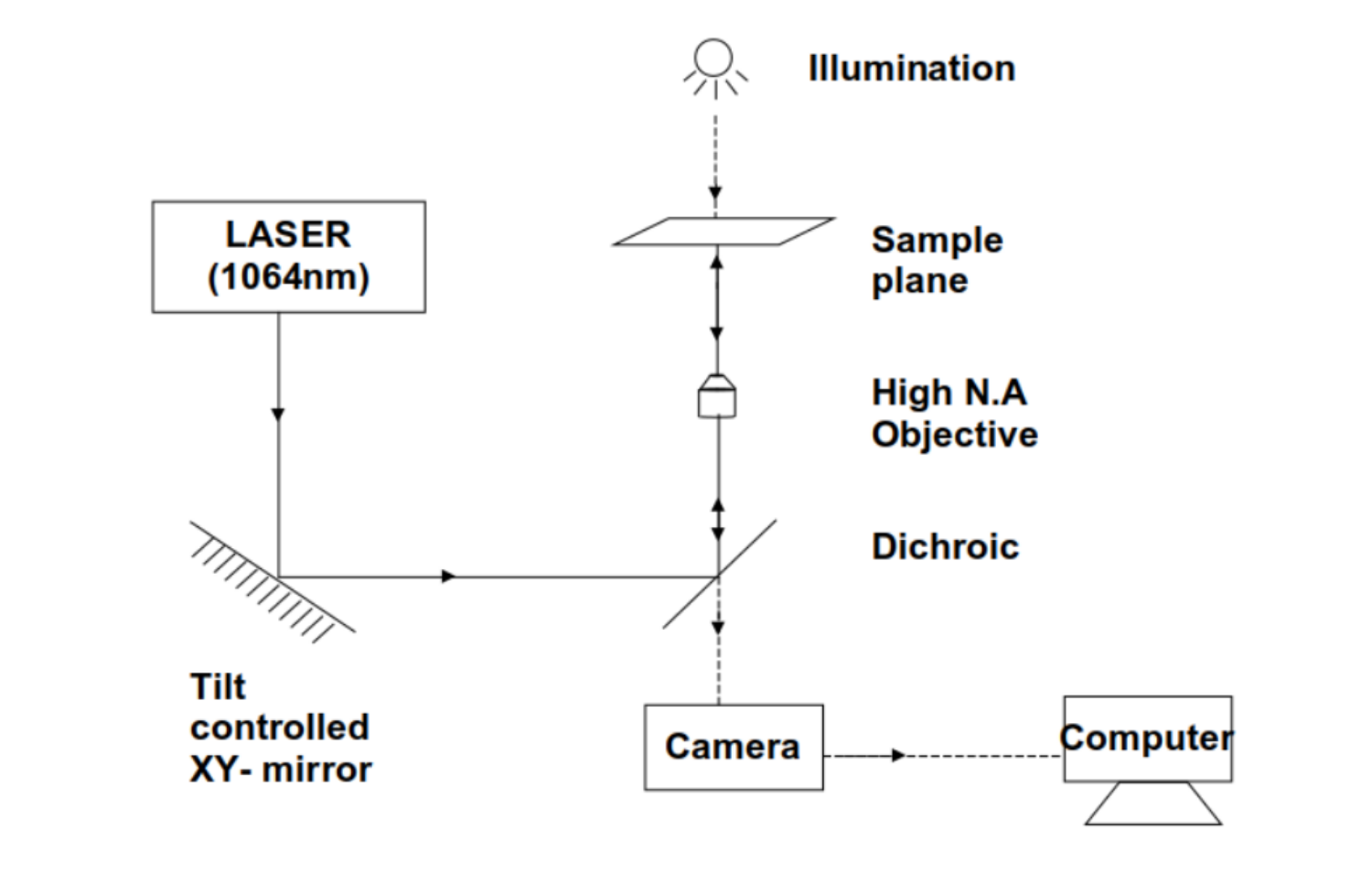

Supplement: S1 Fig — (TIF) [file pone.0226640.s002.tif]

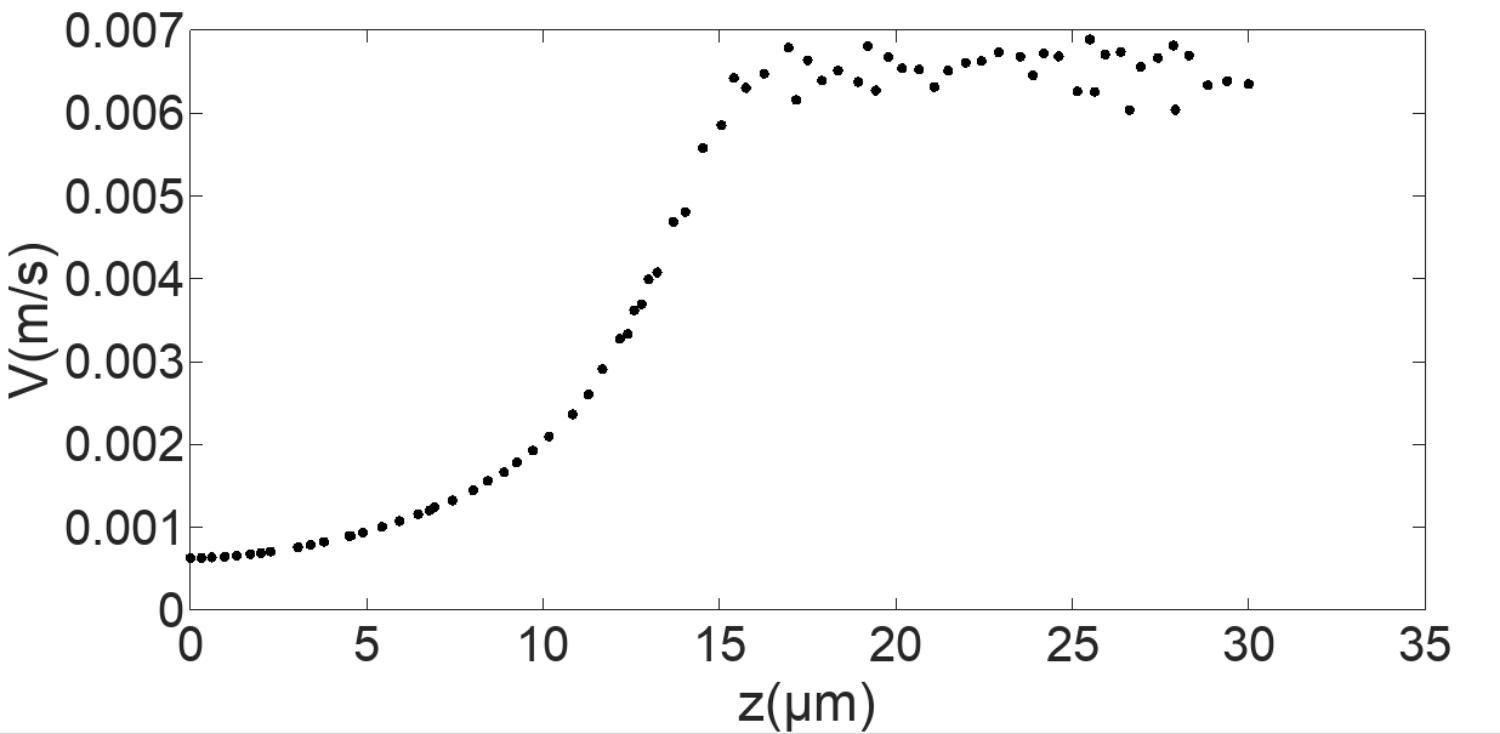

Supplement: S2 Fig — The pore entrance is located at z = 15μm. (TIF) [file pone.0226640.s003.tif]

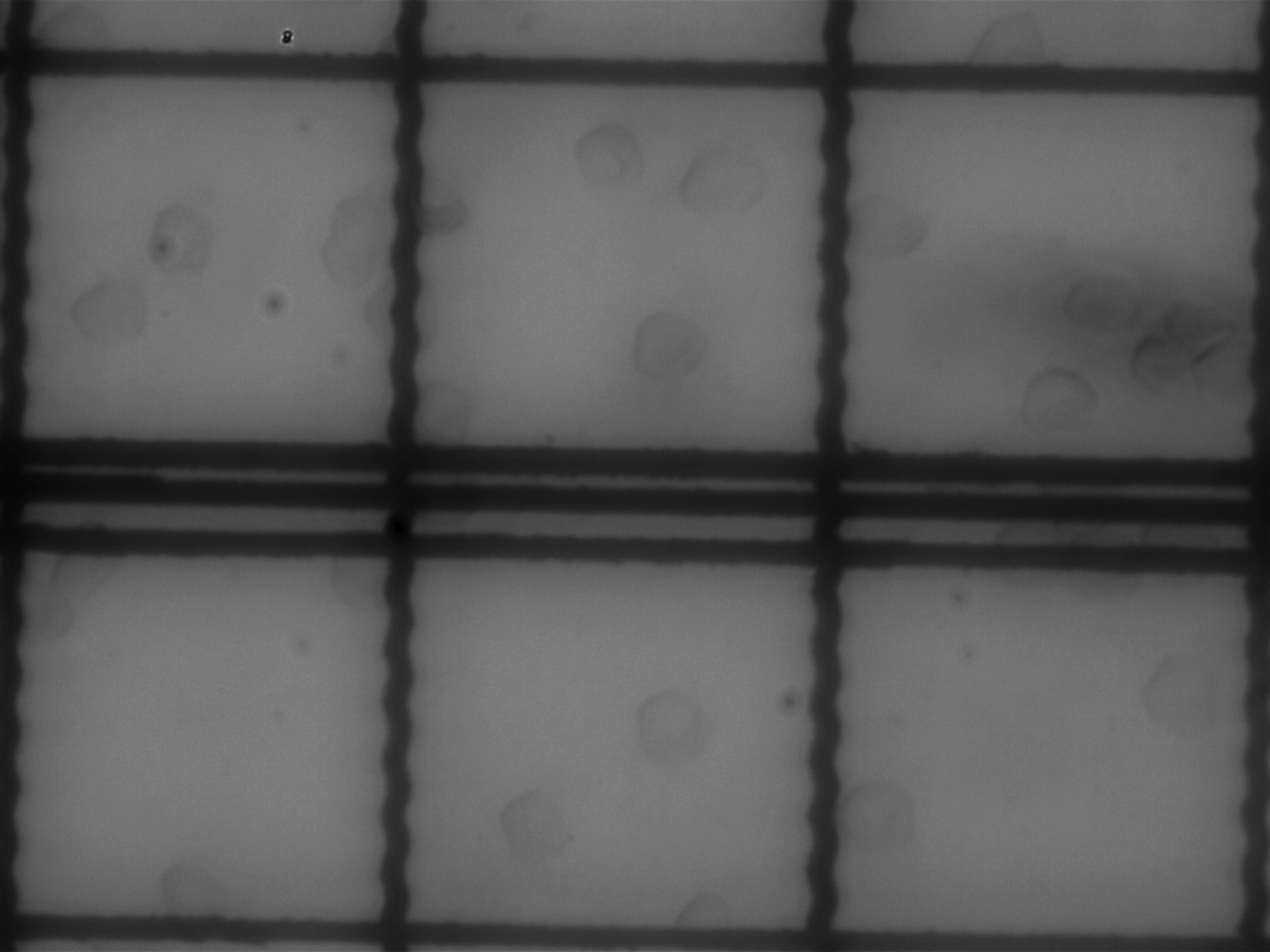

Supplement: S1 Image — (ZIP) [file pone.0226640.s004.zip › S1_imageseq_5_0.5_D2/D2-0001.png]

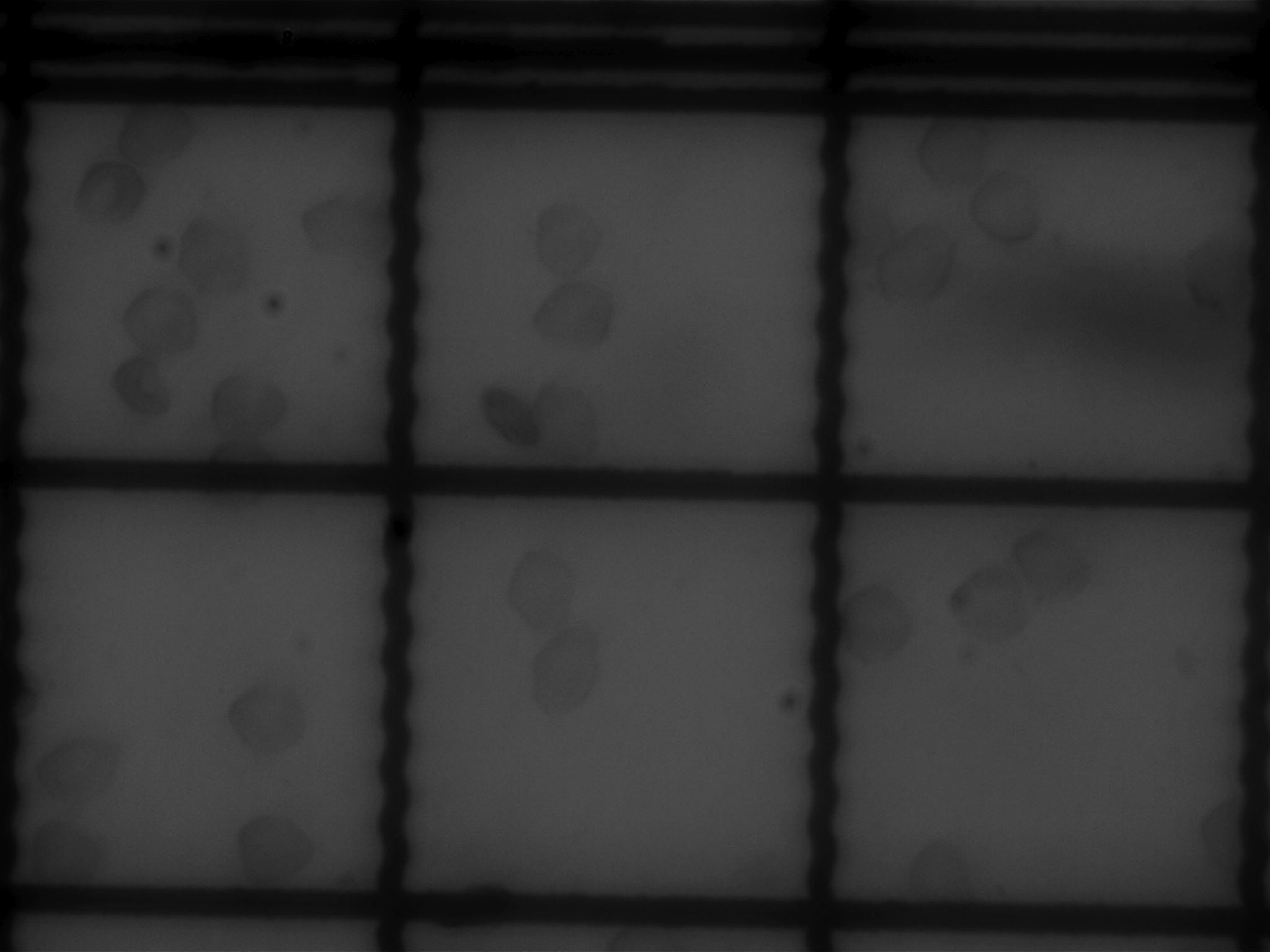

Supplement: S1 Image — (ZIP) [file pone.0226640.s004.zip › S1_imageseq_5_0.5_D2/D2-0002.png]

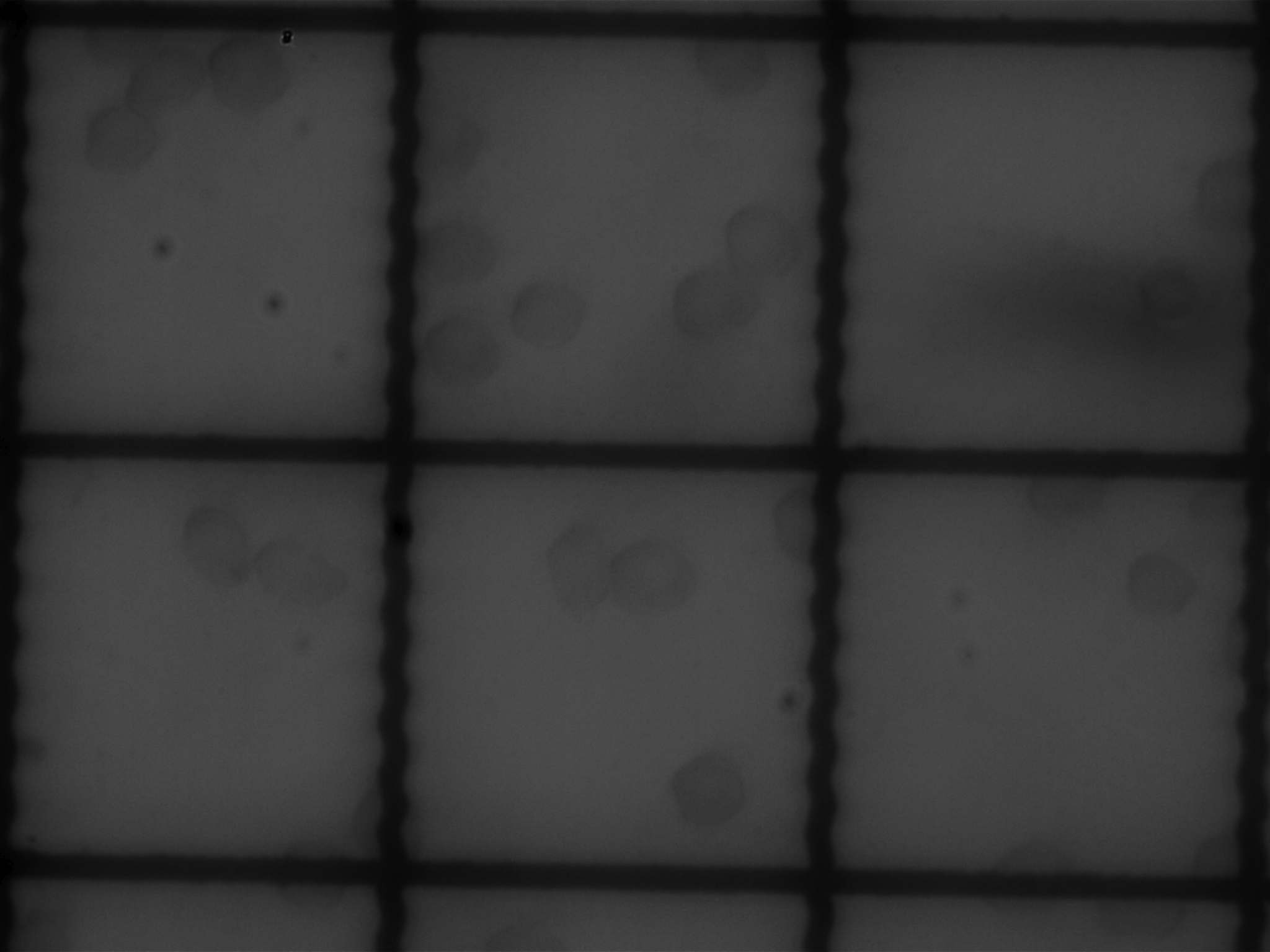

Supplement: S1 Image — (ZIP) [file pone.0226640.s004.zip › S1_imageseq_5_0.5_D2/D2-0003.png]

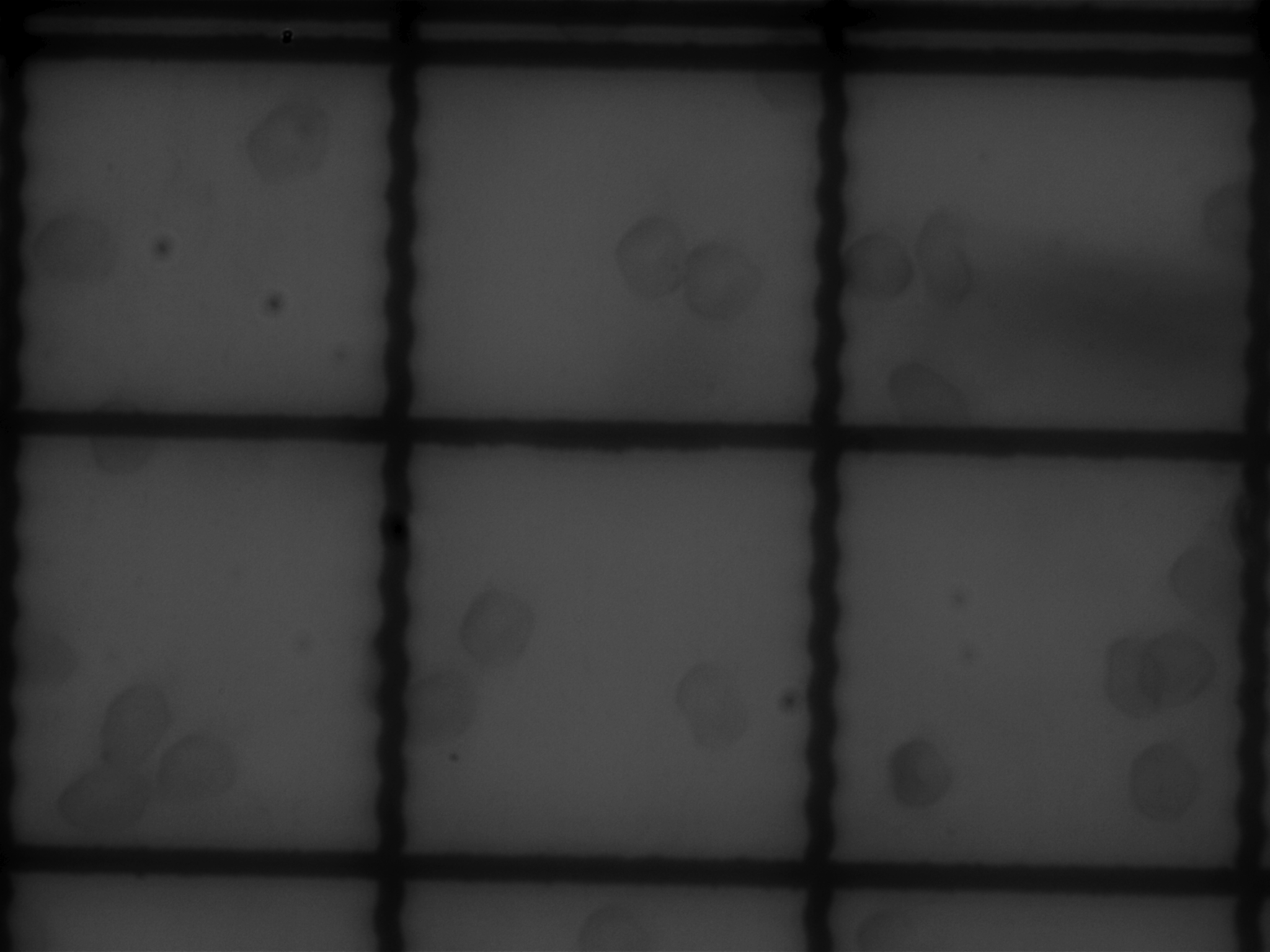

Supplement: S1 Image — (ZIP) [file pone.0226640.s004.zip › S1_imageseq_5_0.5_D2/D2-0004.png]

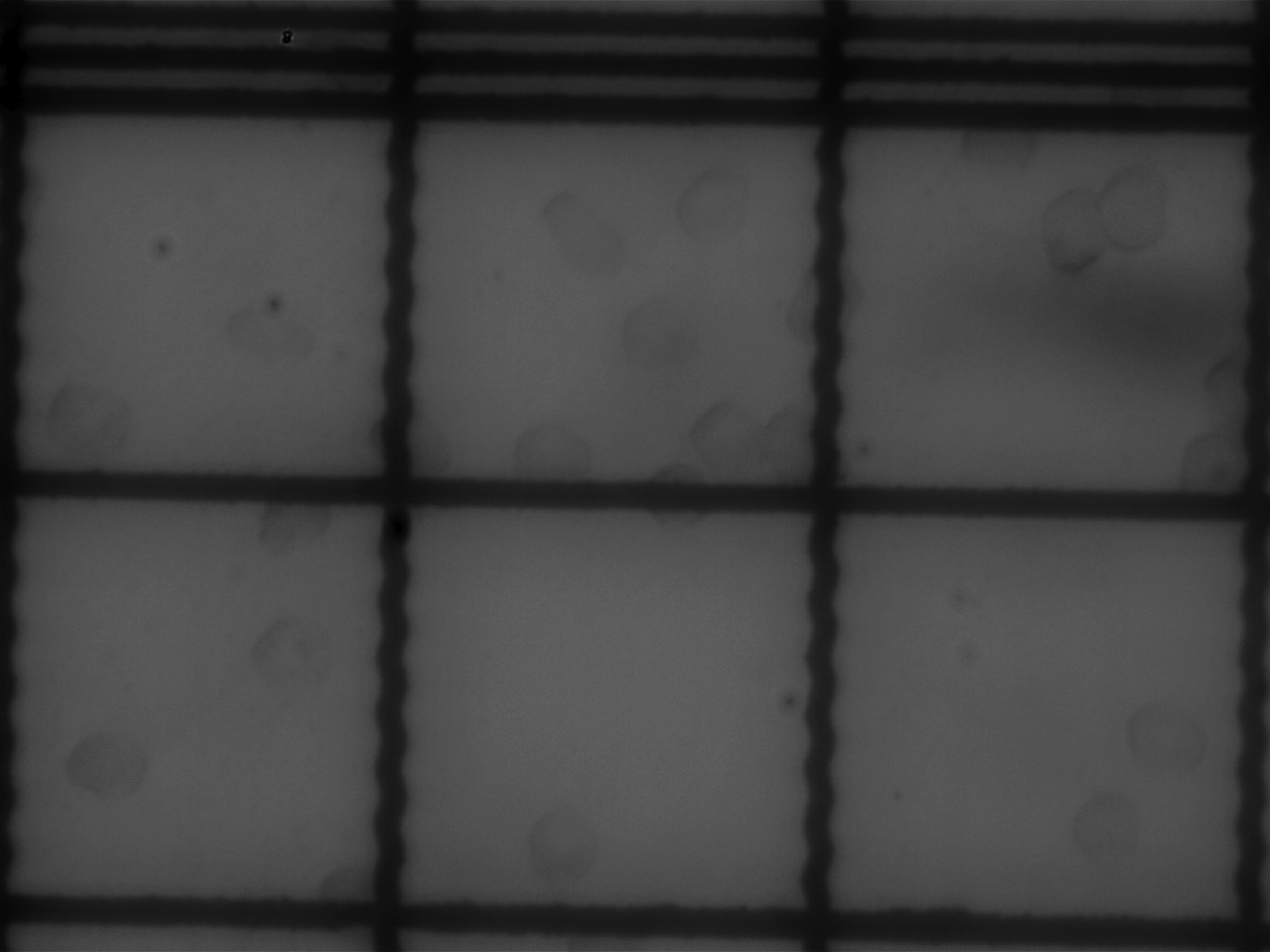

Supplement: S1 Image — (ZIP) [file pone.0226640.s004.zip › S1_imageseq_5_0.5_D2/D2-0005.png]

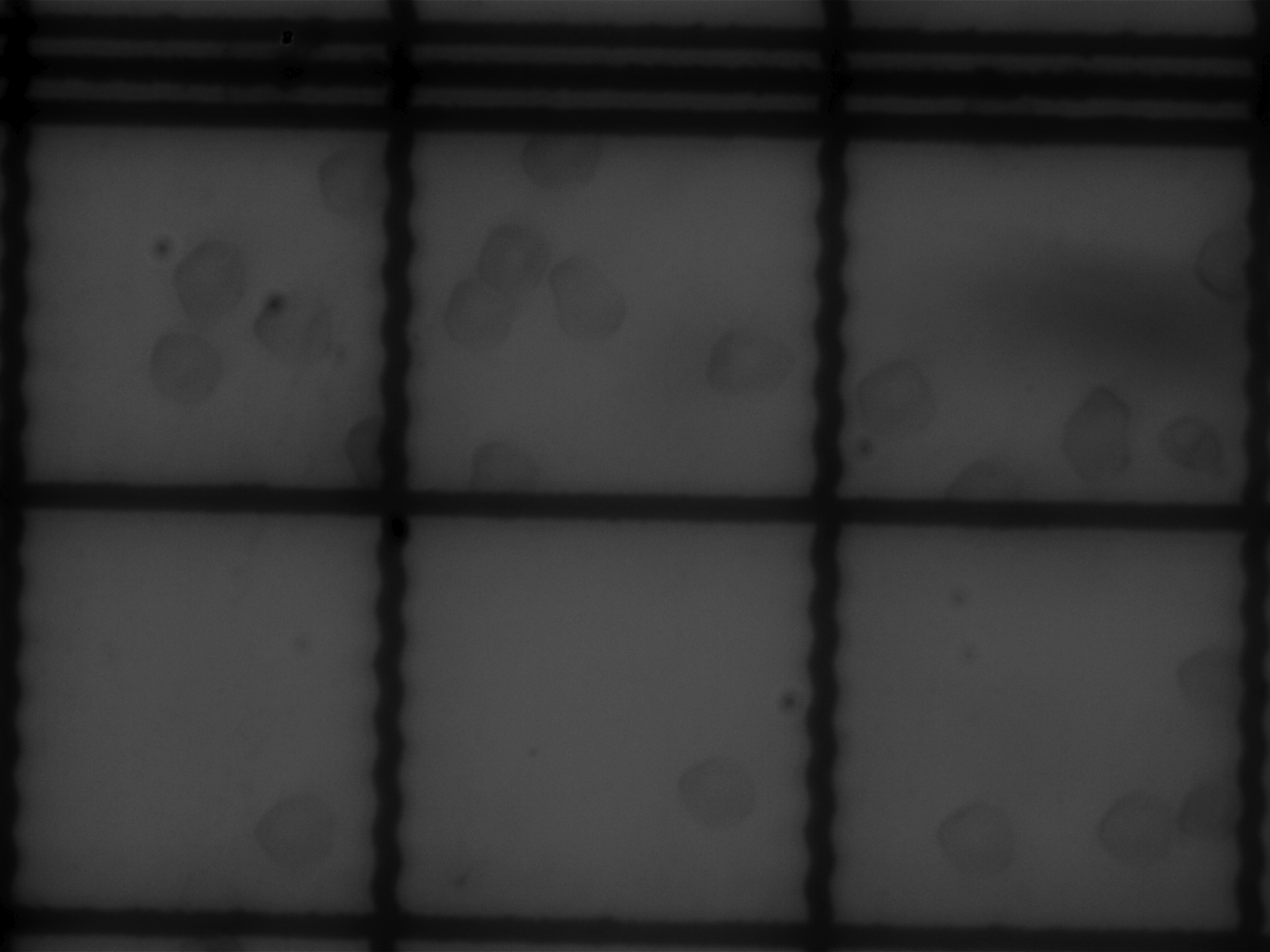

Supplement: S1 Image — (ZIP) [file pone.0226640.s004.zip › S1_imageseq_5_0.5_D2/D2-0006.png]

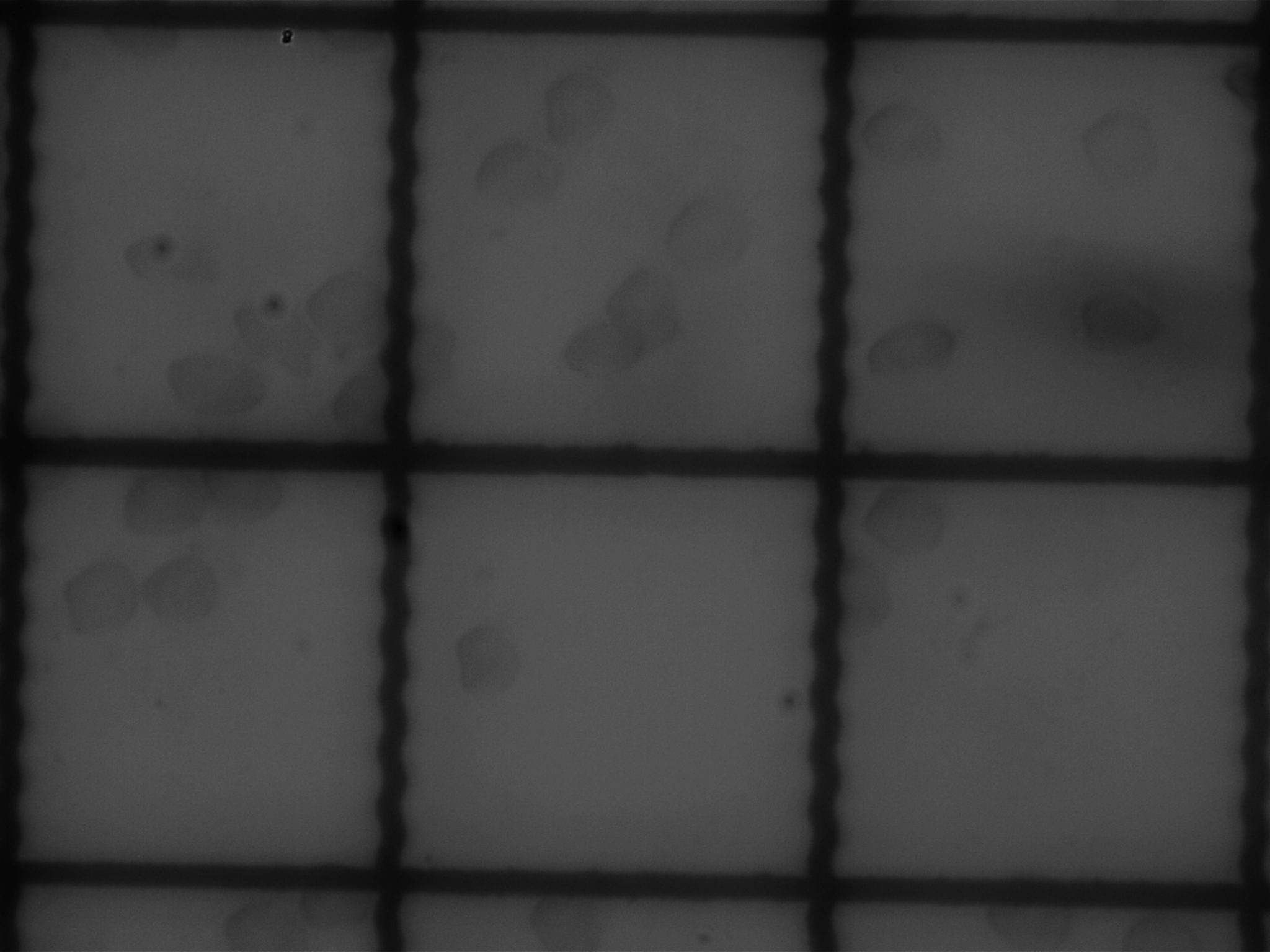

Supplement: S1 Image — (ZIP) [file pone.0226640.s004.zip › S1_imageseq_5_0.5_D2/D2-0007.png]

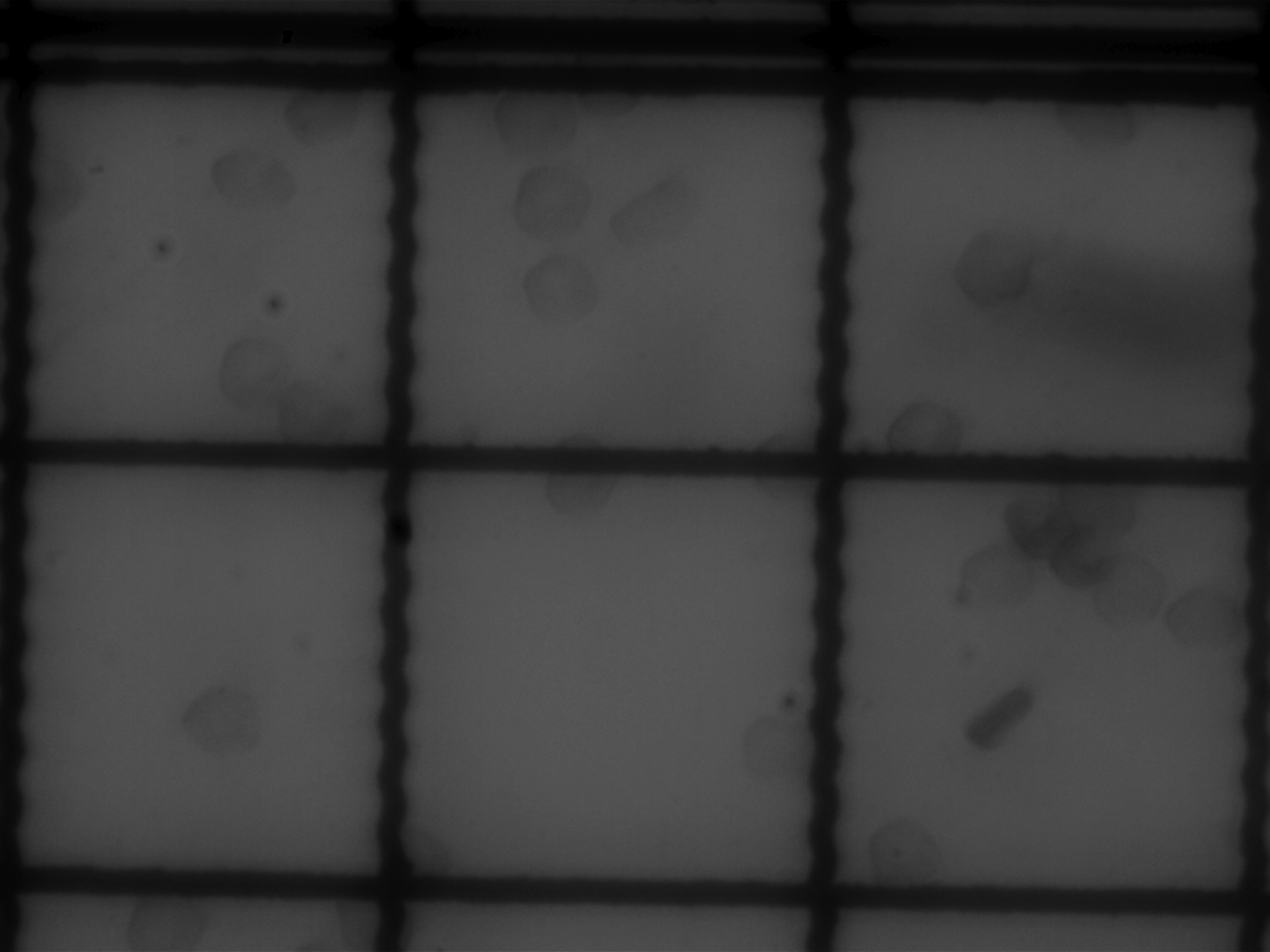

Supplement: S1 Image — (ZIP) [file pone.0226640.s004.zip › S1_imageseq_5_0.5_D2/D2-0008.png]

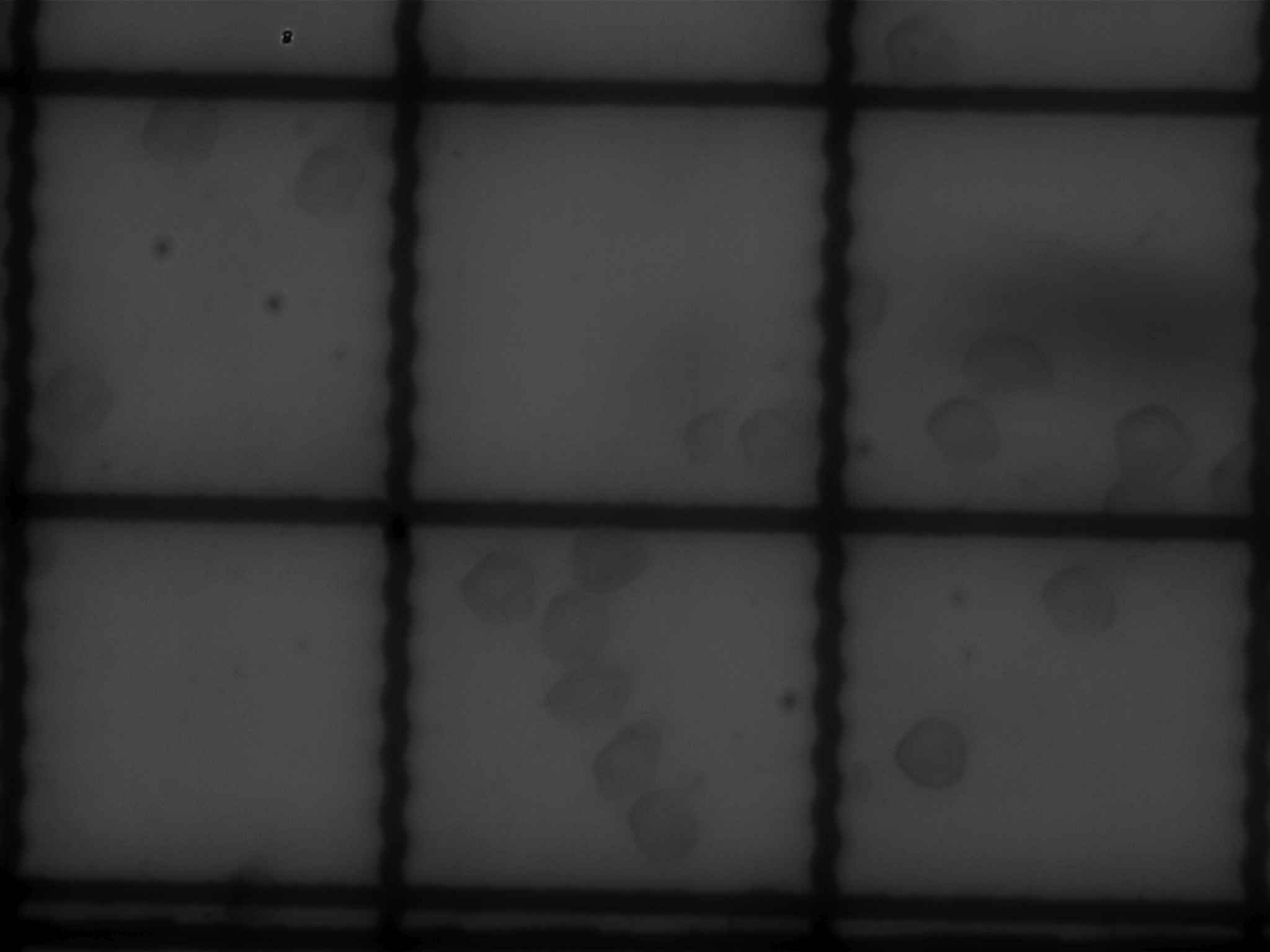

Supplement: S1 Image — (ZIP) [file pone.0226640.s004.zip › S1_imageseq_5_0.5_D2/D2-0009.png]

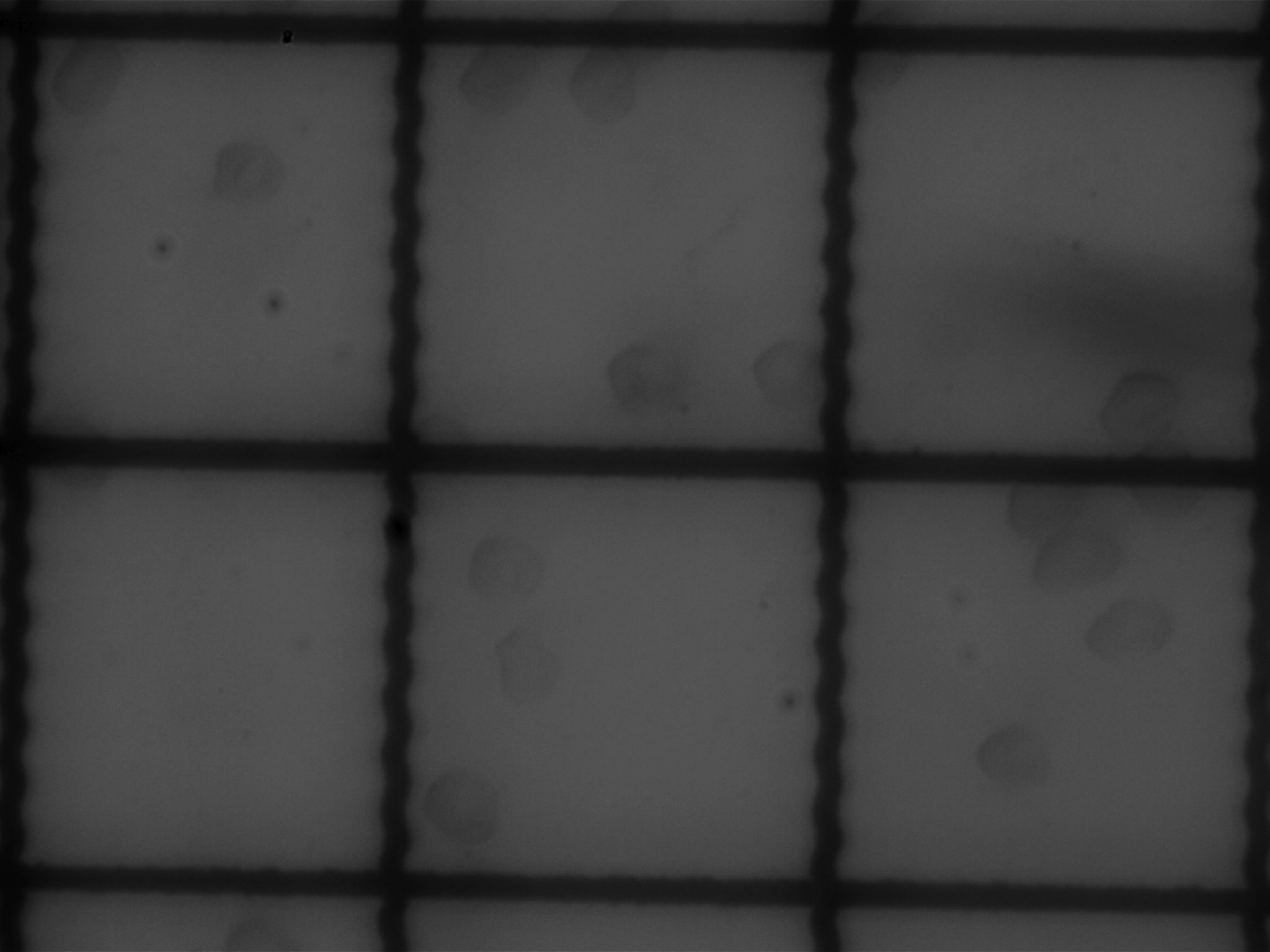

Supplement: S1 Image — (ZIP) [file pone.0226640.s004.zip › S1_imageseq_5_0.5_D2/D2-0010.png]

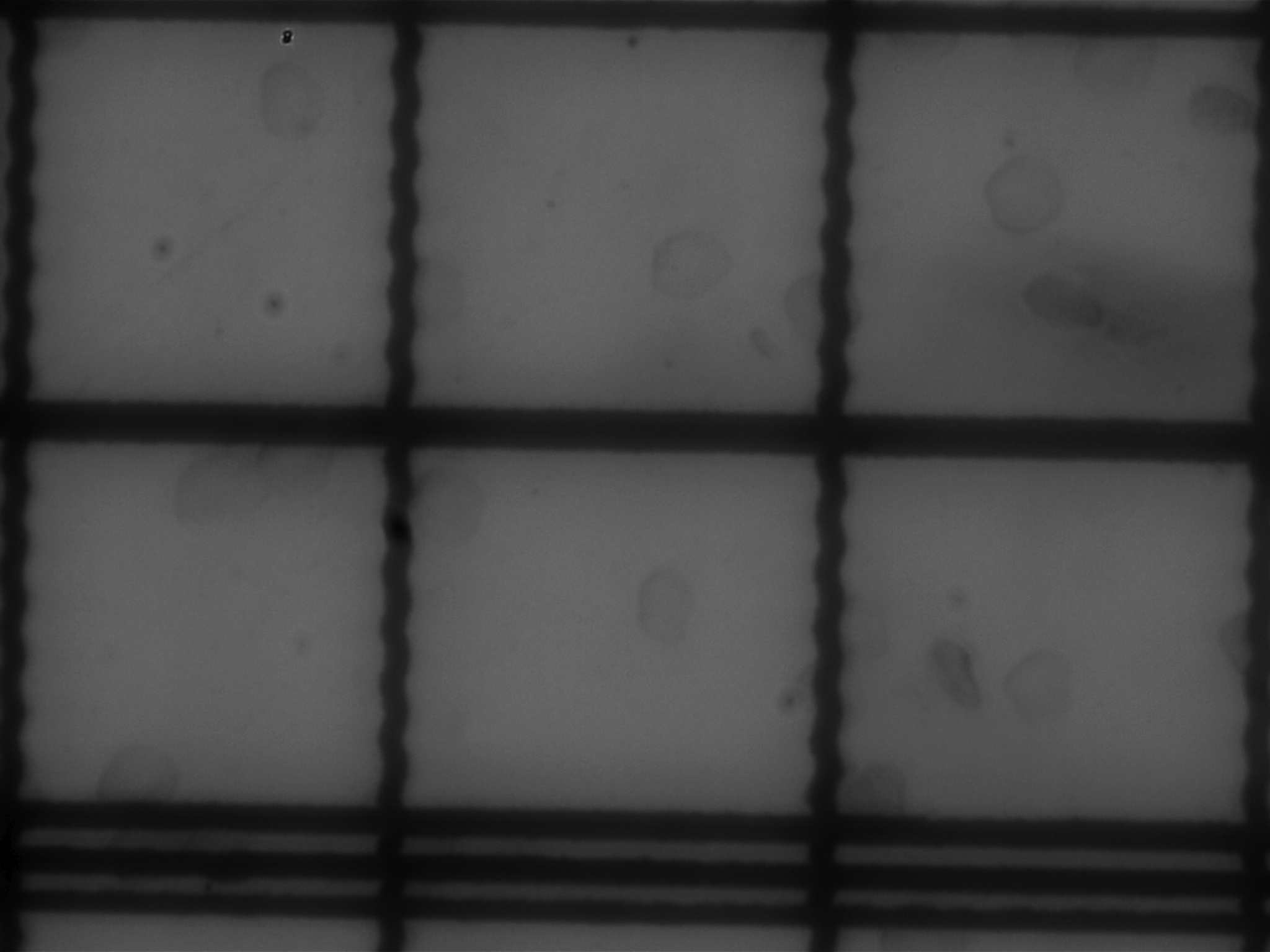

Supplement: S1 Image — (ZIP) [file pone.0226640.s004.zip › S1_imageseq_5_0.5_D2/D2-0011.png]

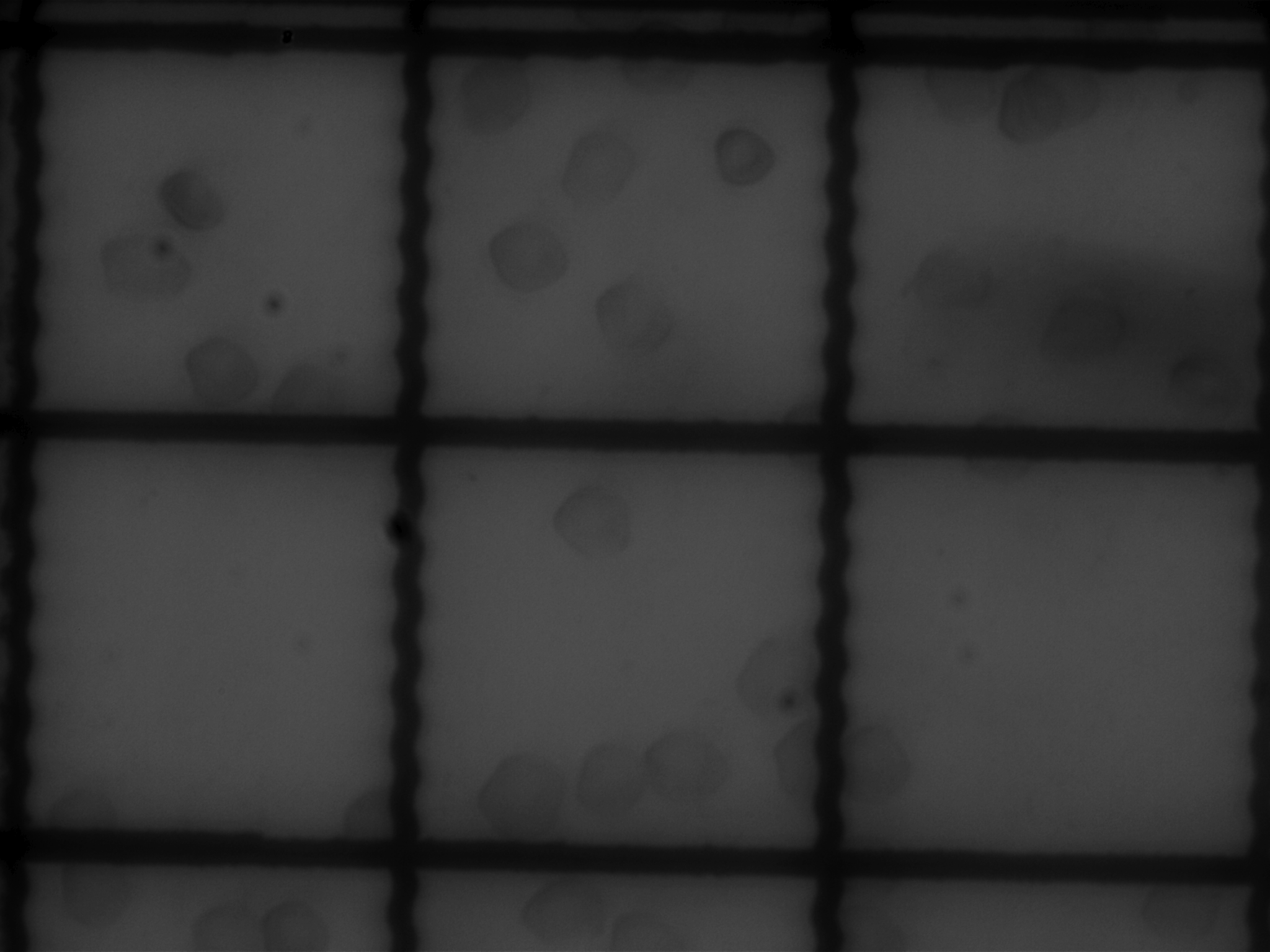

Supplement: S1 Image — (ZIP) [file pone.0226640.s004.zip › S1_imageseq_5_0.5_D2/D2-0012.png]

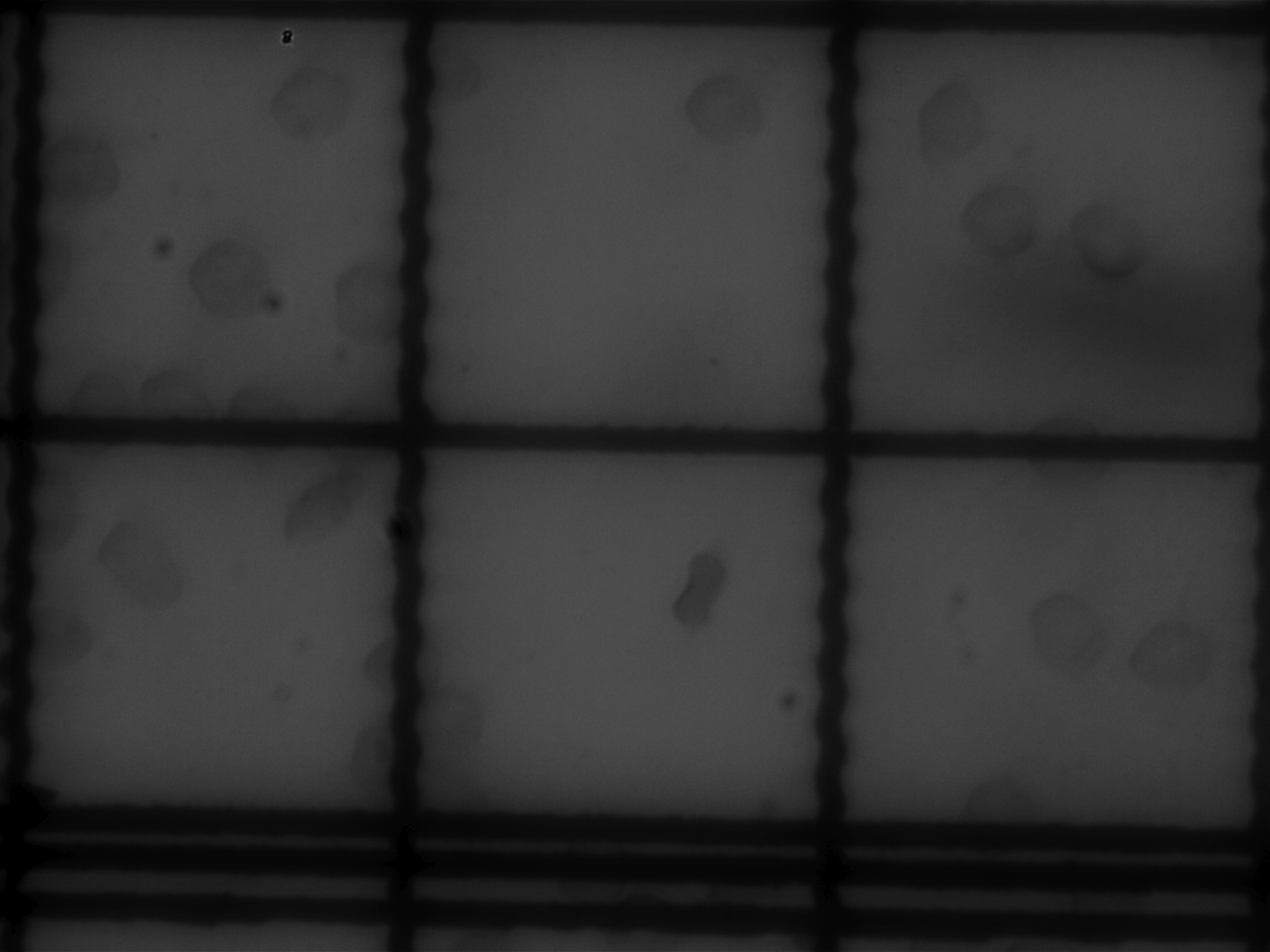

Supplement: S1 Image — (ZIP) [file pone.0226640.s004.zip › S1_imageseq_5_0.5_D2/D2-0013.png]

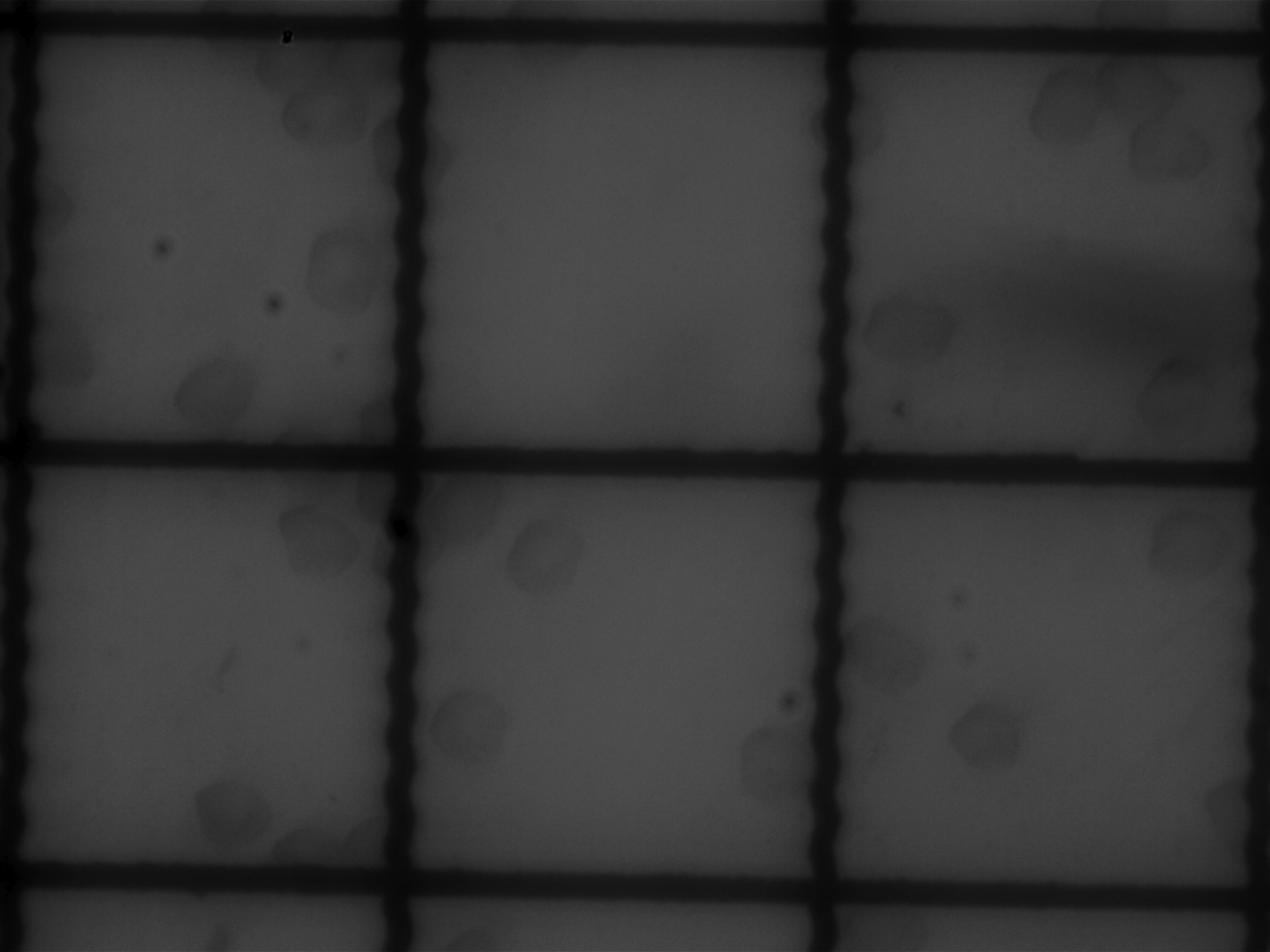

Supplement: S1 Image — (ZIP) [file pone.0226640.s004.zip › S1_imageseq_5_0.5_D2/D2-0014.png]

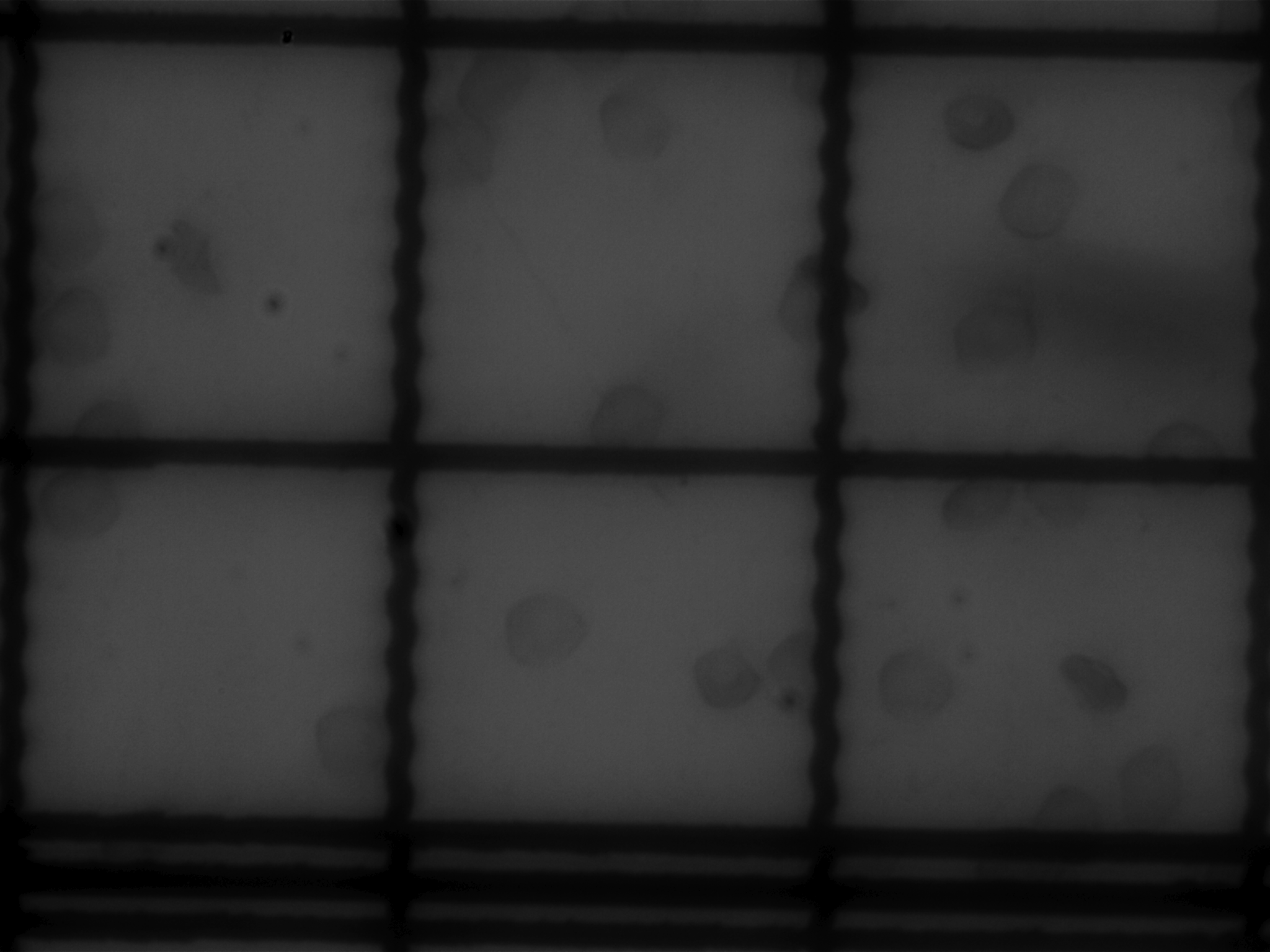

Supplement: S1 Image — (ZIP) [file pone.0226640.s004.zip › S1_imageseq_5_0.5_D2/D2-0015.png]

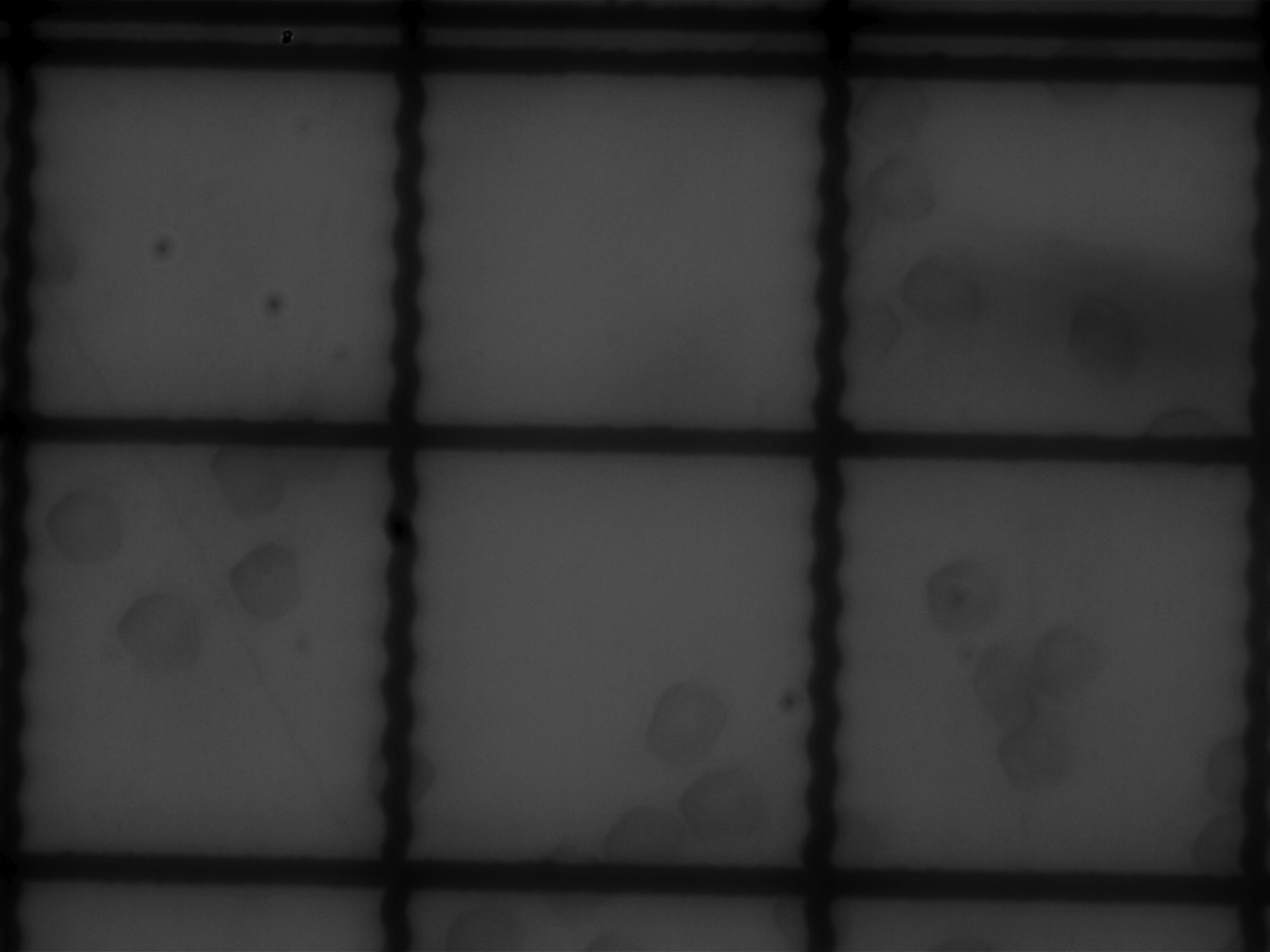

Supplement: S1 Image — (ZIP) [file pone.0226640.s004.zip › S1_imageseq_5_0.5_D2/D2-0016.png]

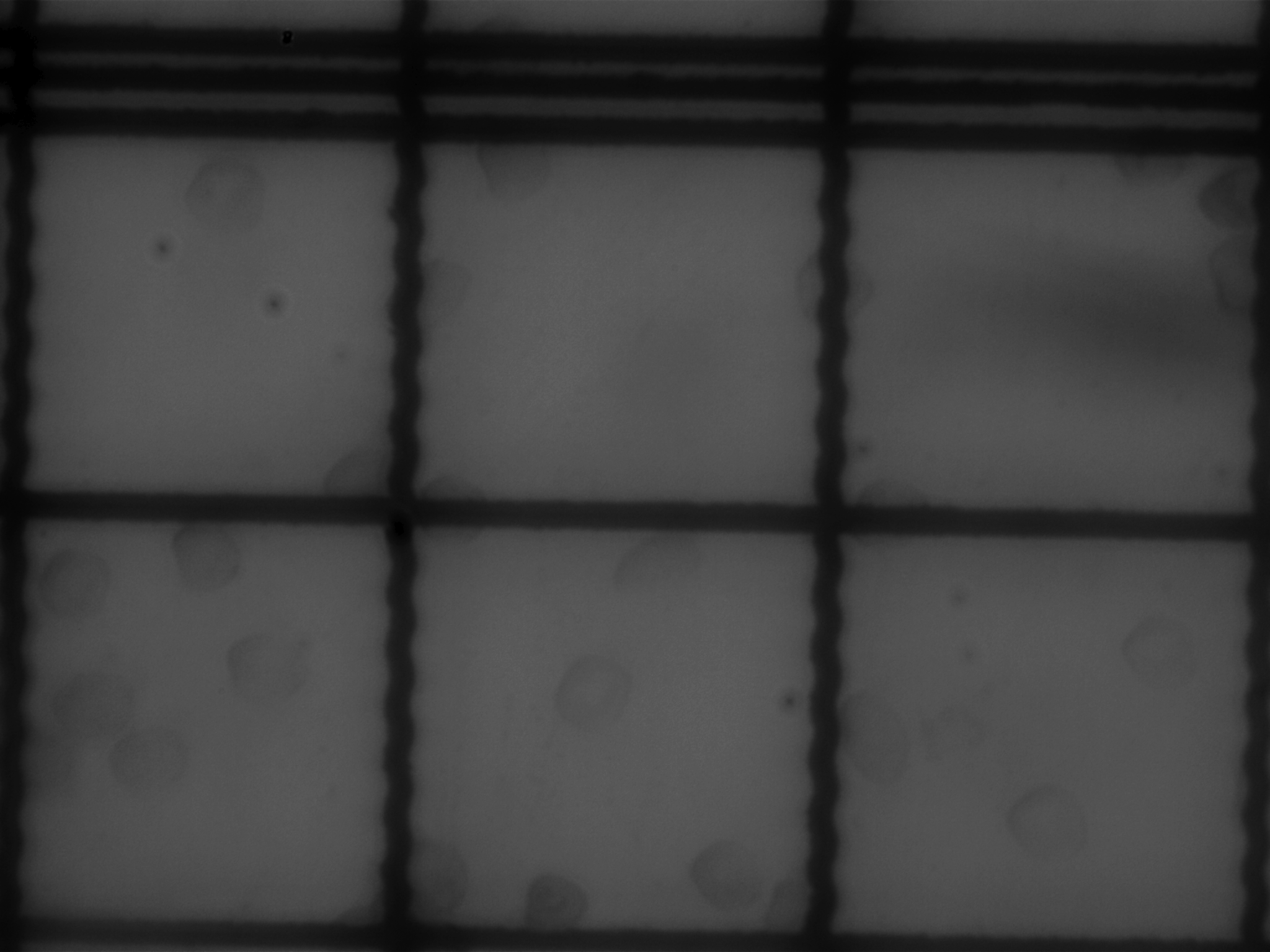

Supplement: S1 Image — (ZIP) [file pone.0226640.s004.zip › S1_imageseq_5_0.5_D2/D2-0017.png]

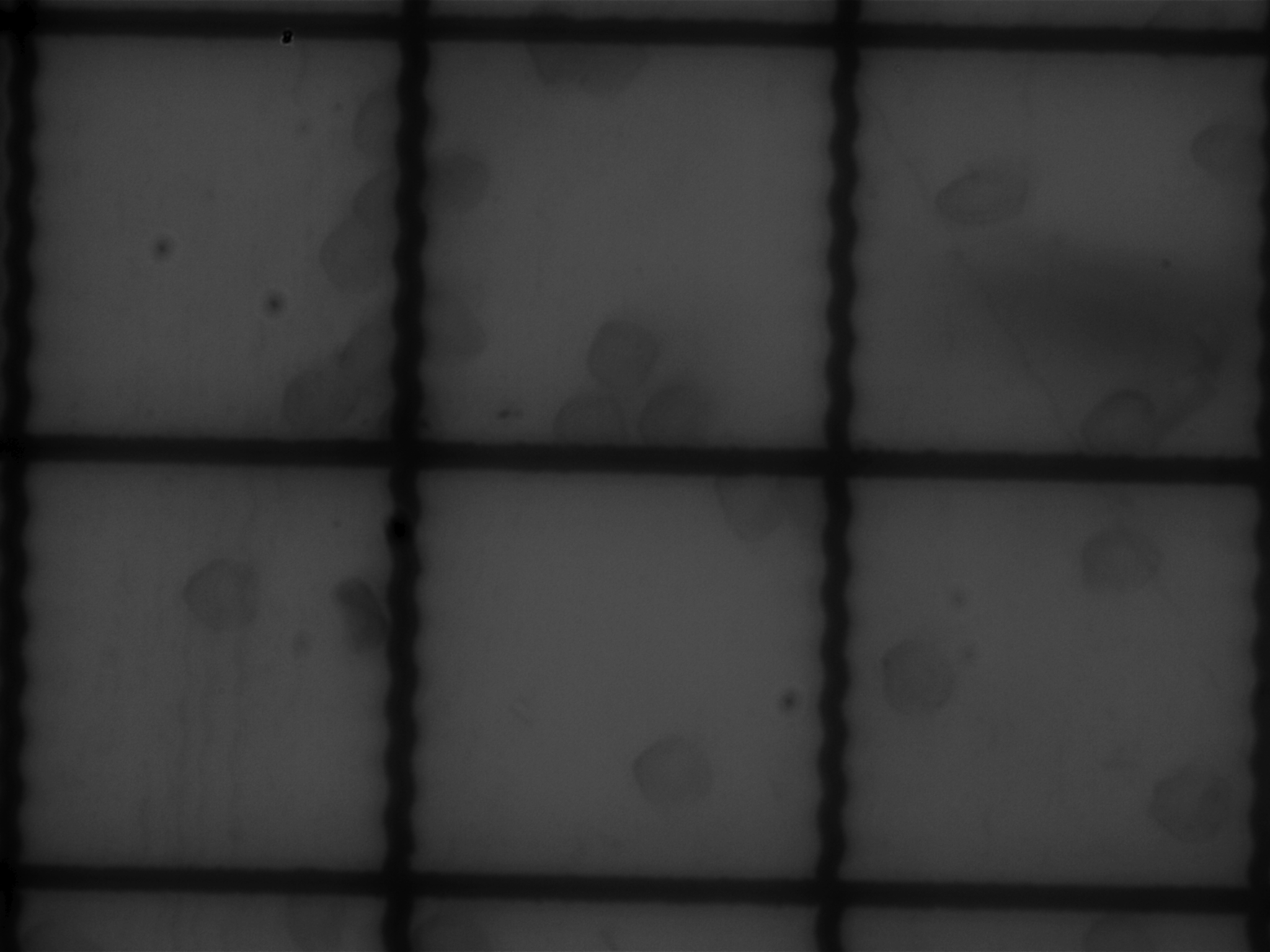

Supplement: S1 Image — (ZIP) [file pone.0226640.s004.zip › S1_imageseq_5_0.5_D2/D2-0018.png]

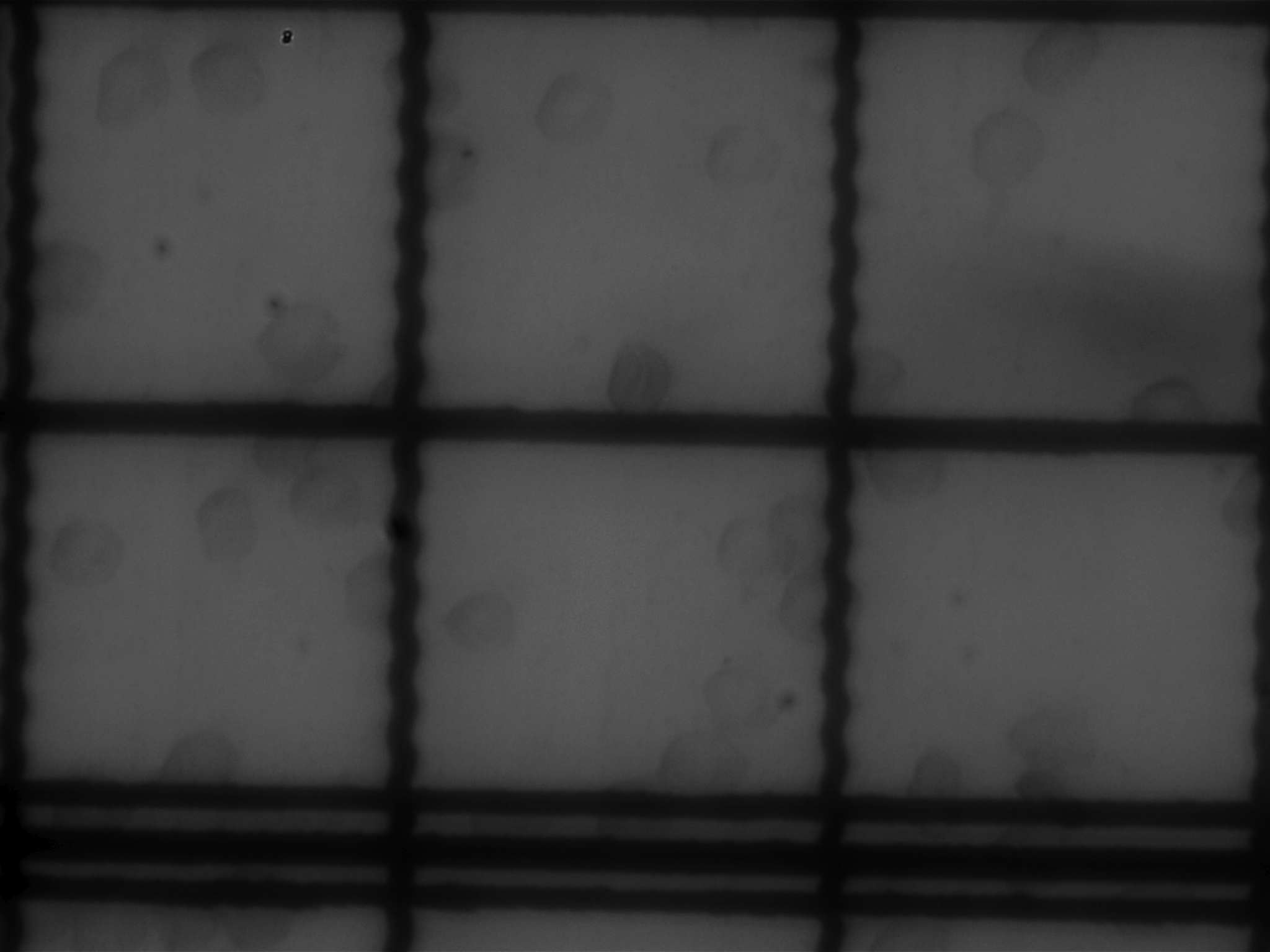

Supplement: S1 Image — (ZIP) [file pone.0226640.s004.zip › S1_imageseq_5_0.5_D2/D2-0019.png]

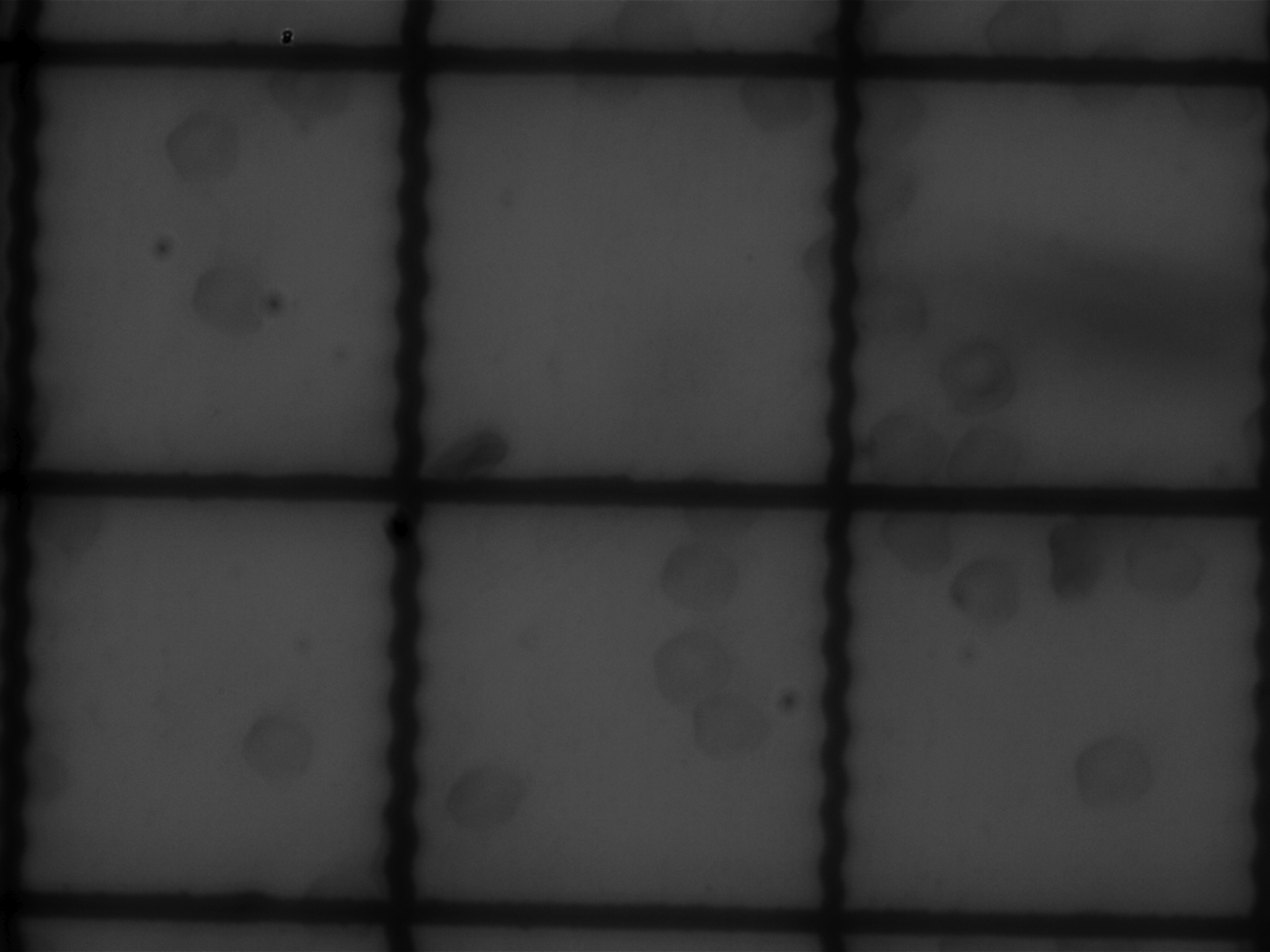

Supplement: S1 Image — (ZIP) [file pone.0226640.s004.zip › S1_imageseq_5_0.5_D2/D2-0020.png]

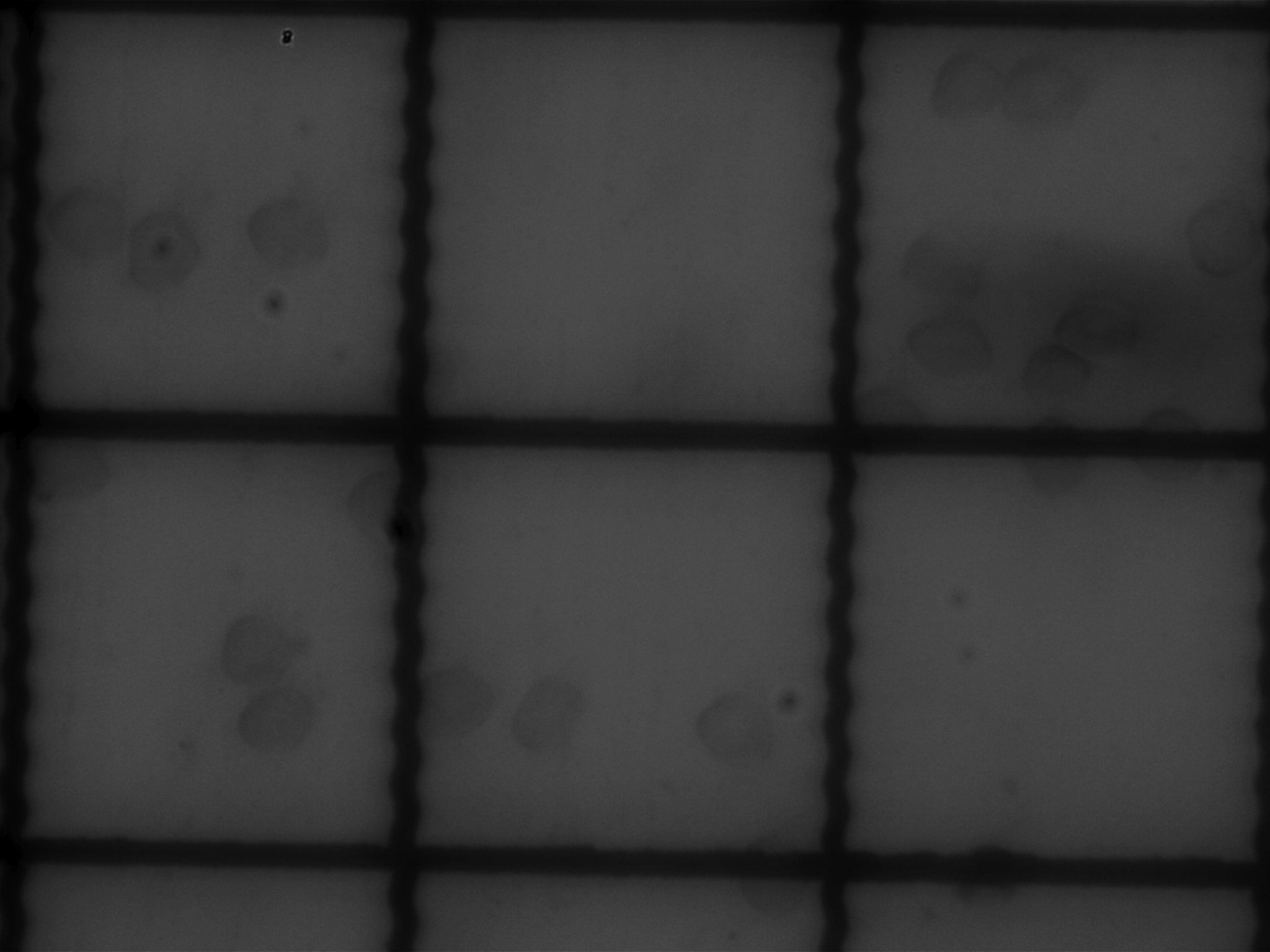

Supplement: S1 Image — (ZIP) [file pone.0226640.s004.zip › S1_imageseq_5_0.5_D2/D2-0021.png]

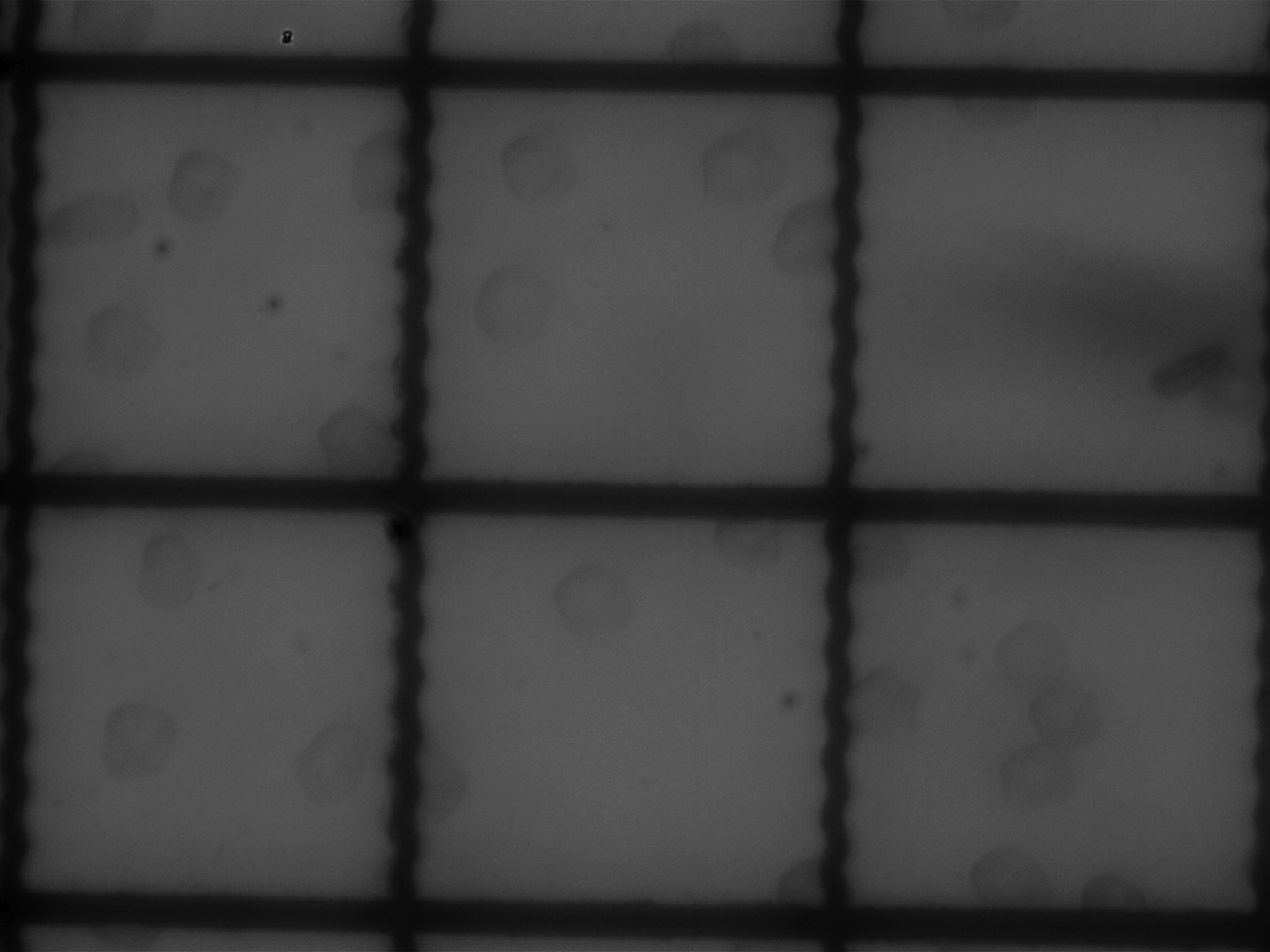

Supplement: S1 Image — (ZIP) [file pone.0226640.s004.zip › S1_imageseq_5_0.5_D2/D2-0022.png]

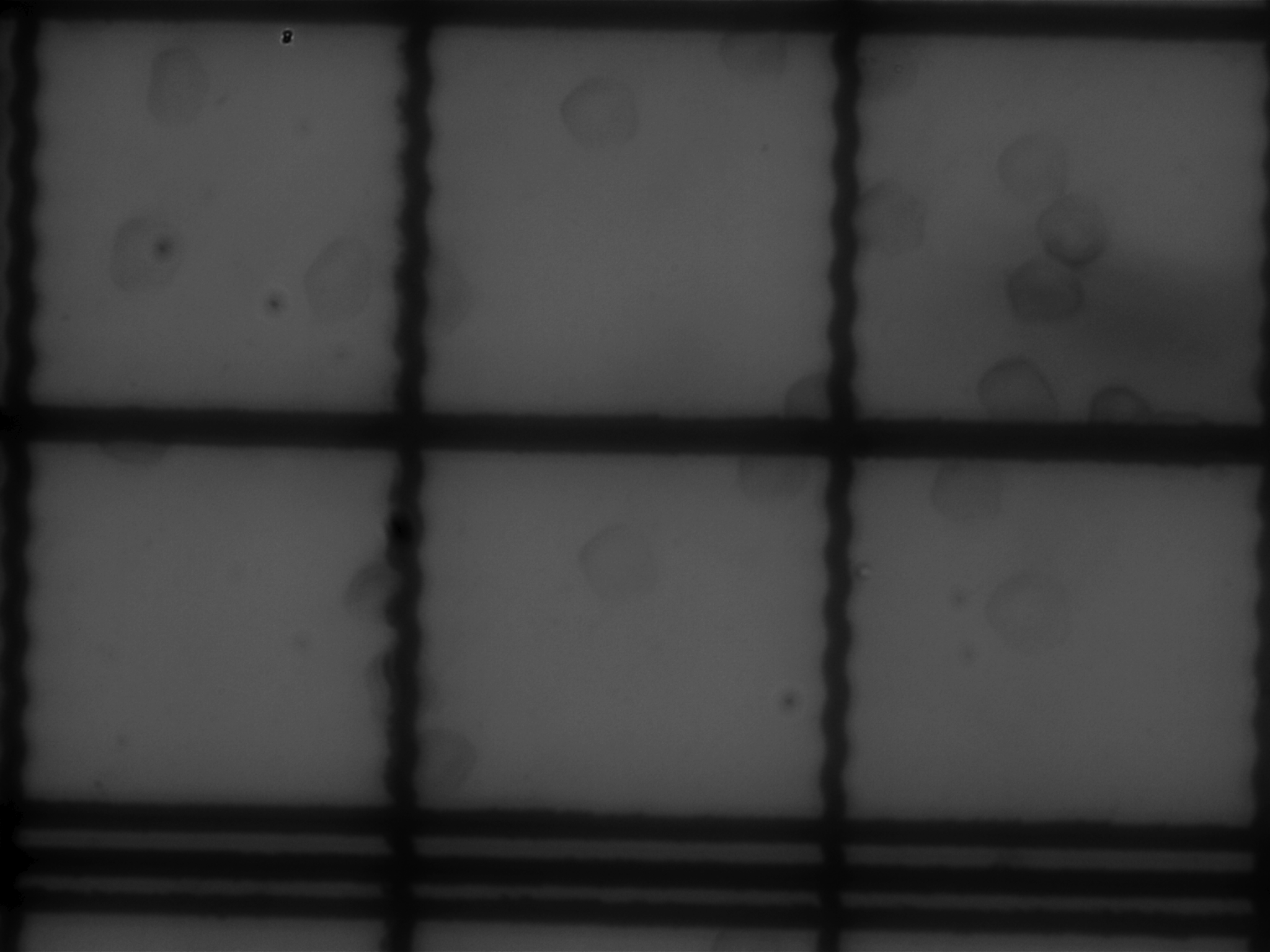

Supplement: S1 Image — (ZIP) [file pone.0226640.s004.zip › S1_imageseq_5_0.5_D2/D2-0023.png]

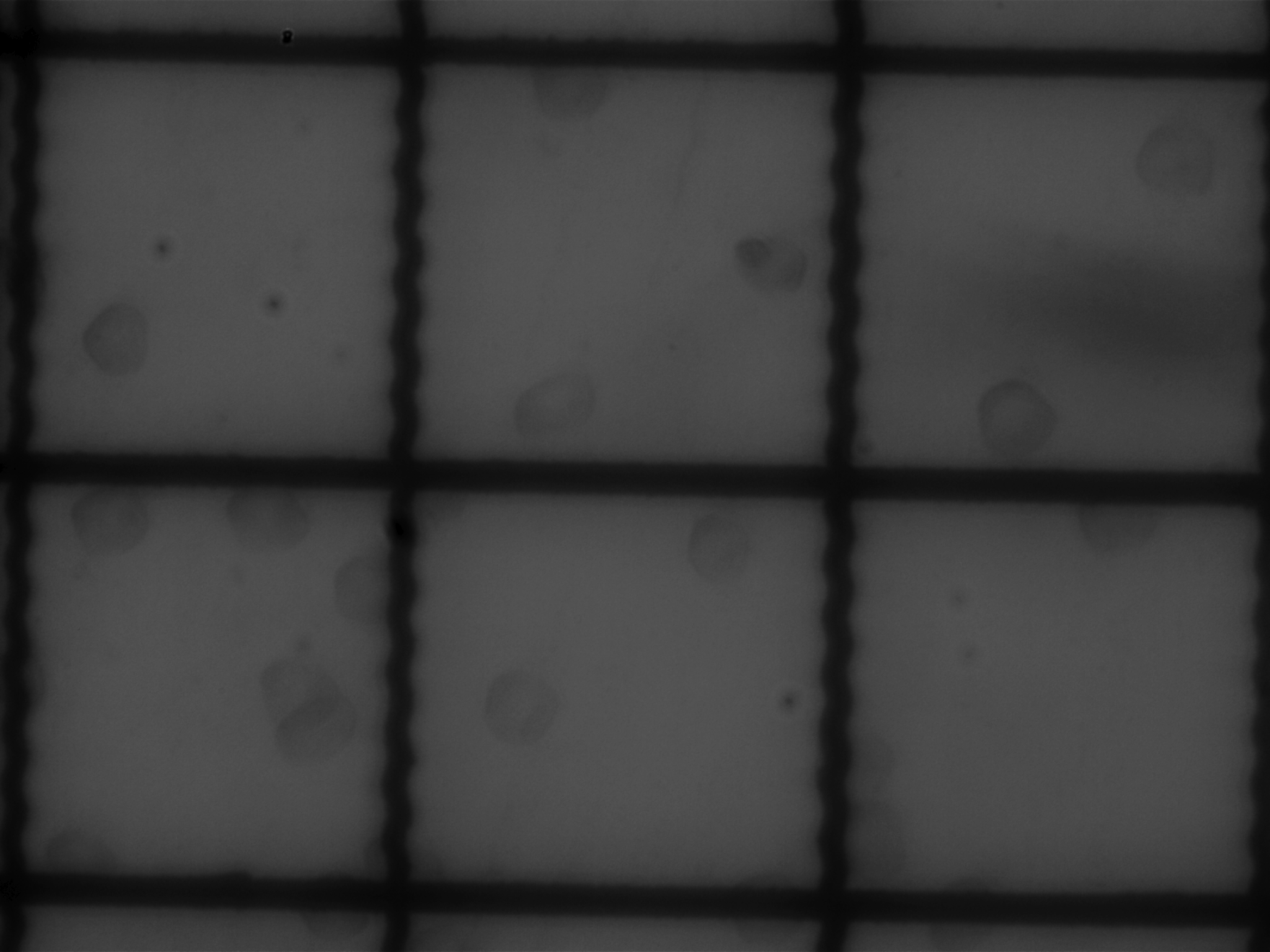

Supplement: S1 Image — (ZIP) [file pone.0226640.s004.zip › S1_imageseq_5_0.5_D2/D2-0024.png]

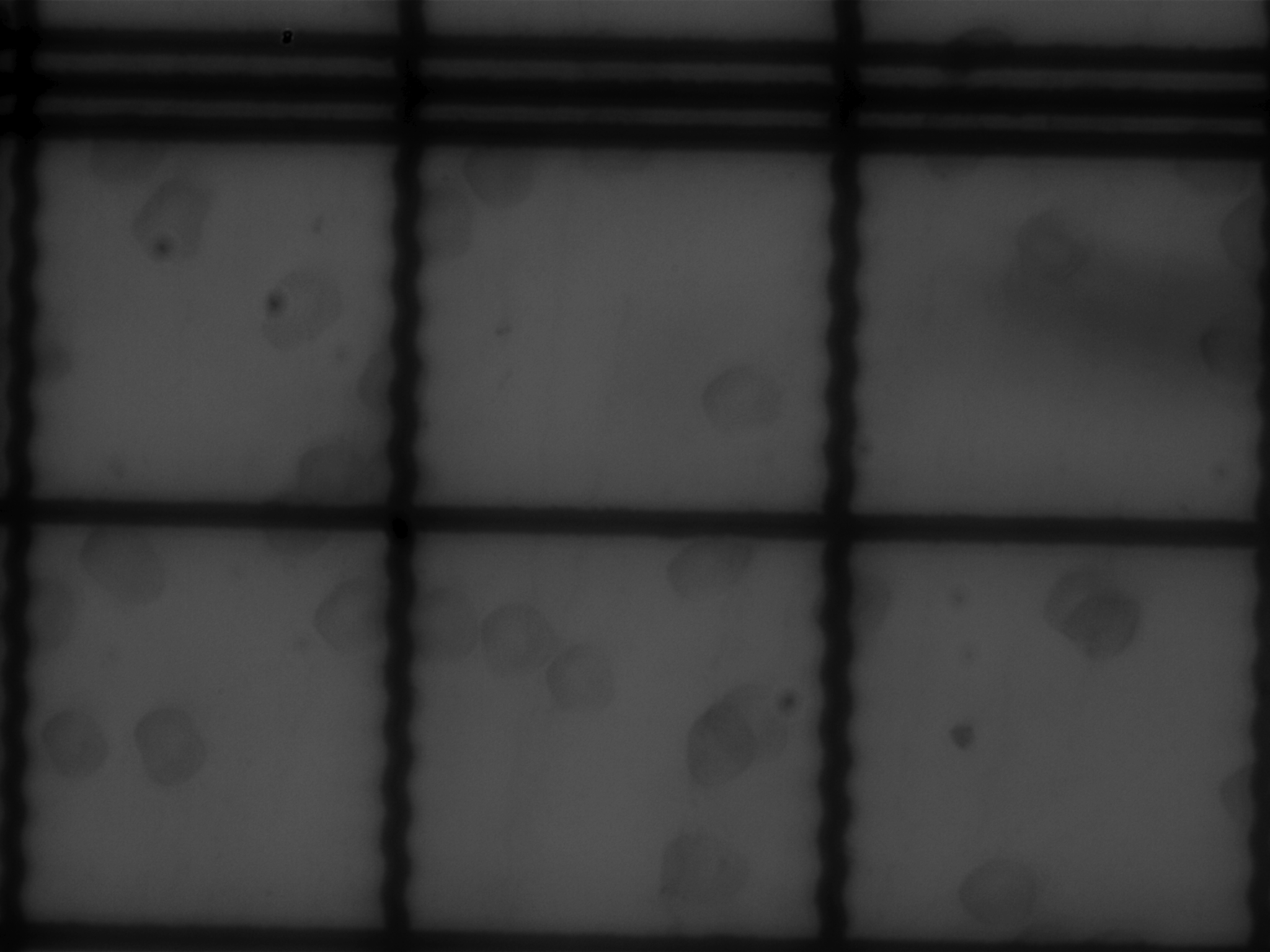

Supplement: S1 Image — (ZIP) [file pone.0226640.s004.zip › S1_imageseq_5_0.5_D2/D2-0025.png]

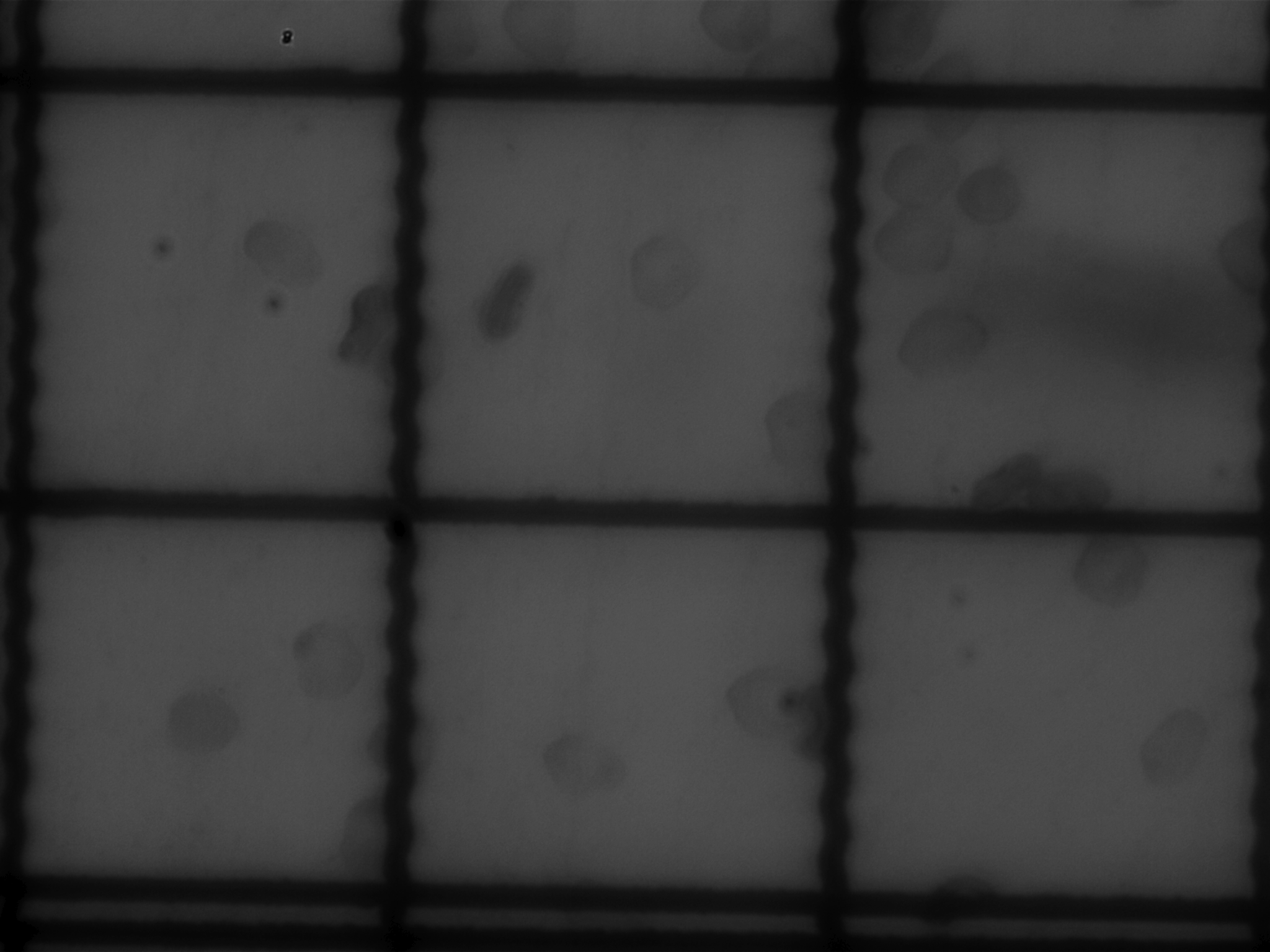

Supplement: S1 Image — (ZIP) [file pone.0226640.s004.zip › S1_imageseq_5_0.5_D2/D2-0026.png]

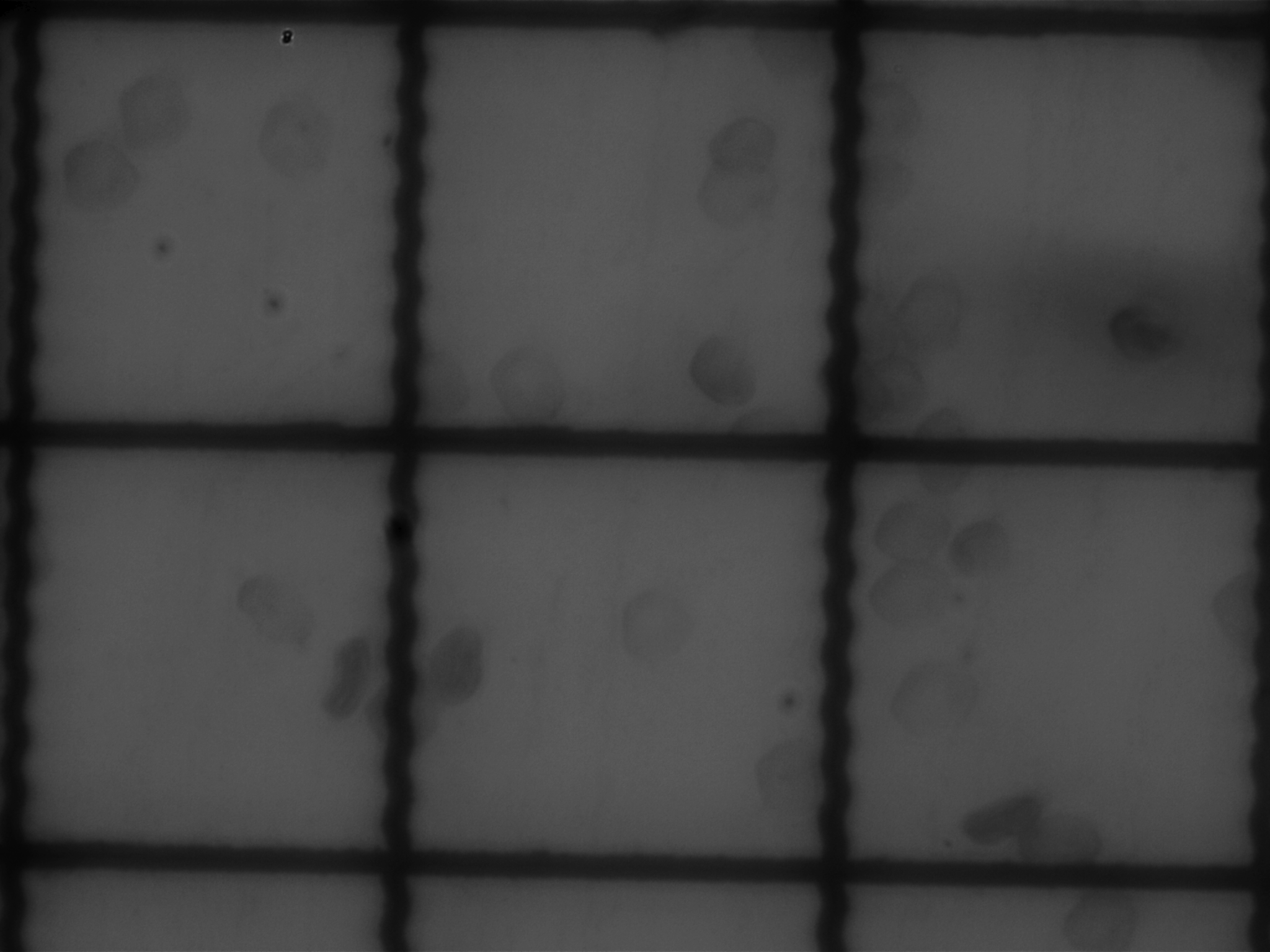

Supplement: S1 Image — (ZIP) [file pone.0226640.s004.zip › S1_imageseq_5_0.5_D2/D2-0027.png]

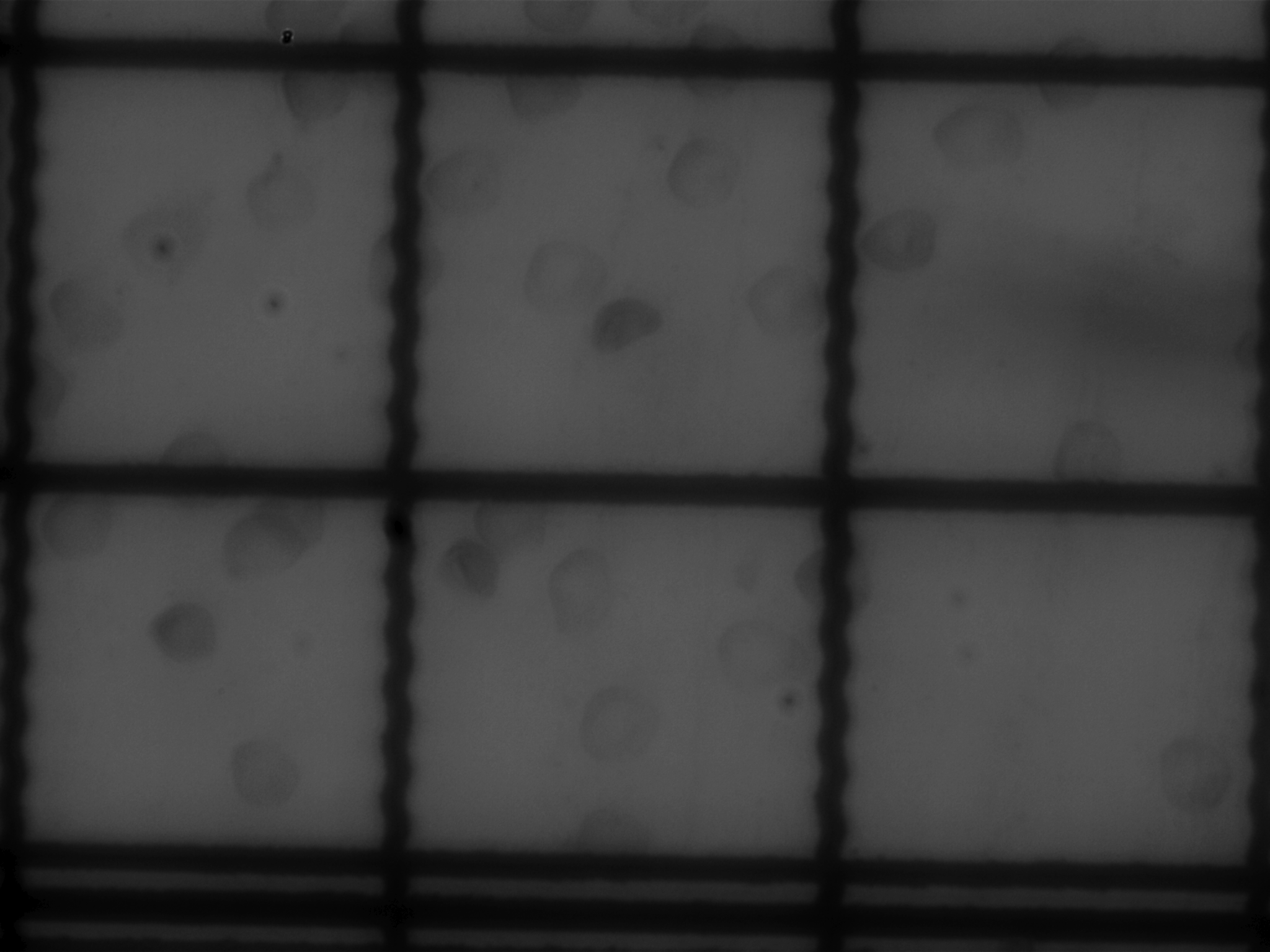

Supplement: S1 Image — (ZIP) [file pone.0226640.s004.zip › S1_imageseq_5_0.5_D2/D2-0028.png]

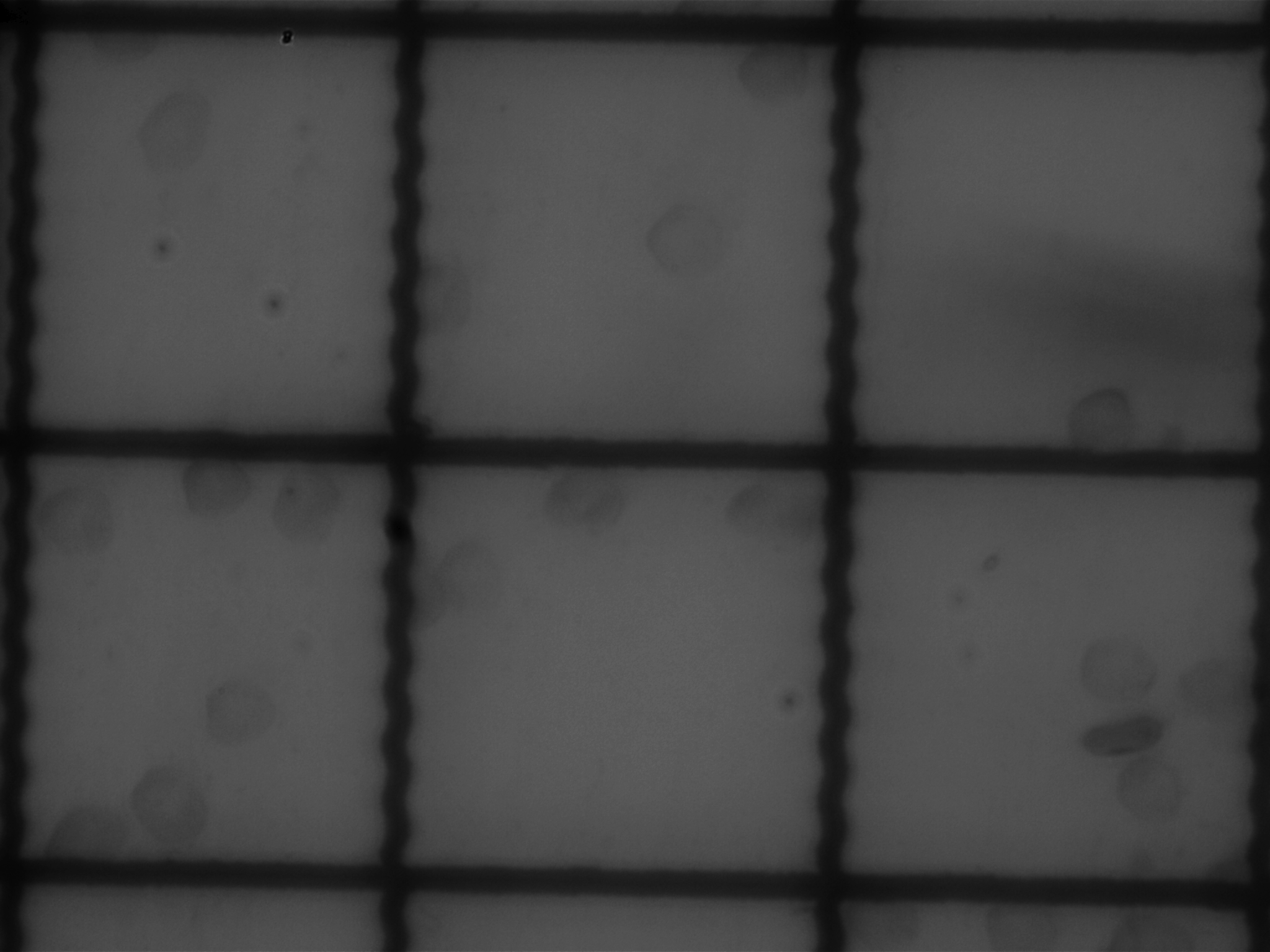

Supplement: S1 Image — (ZIP) [file pone.0226640.s004.zip › S1_imageseq_5_0.5_D2/D2-0029.png]

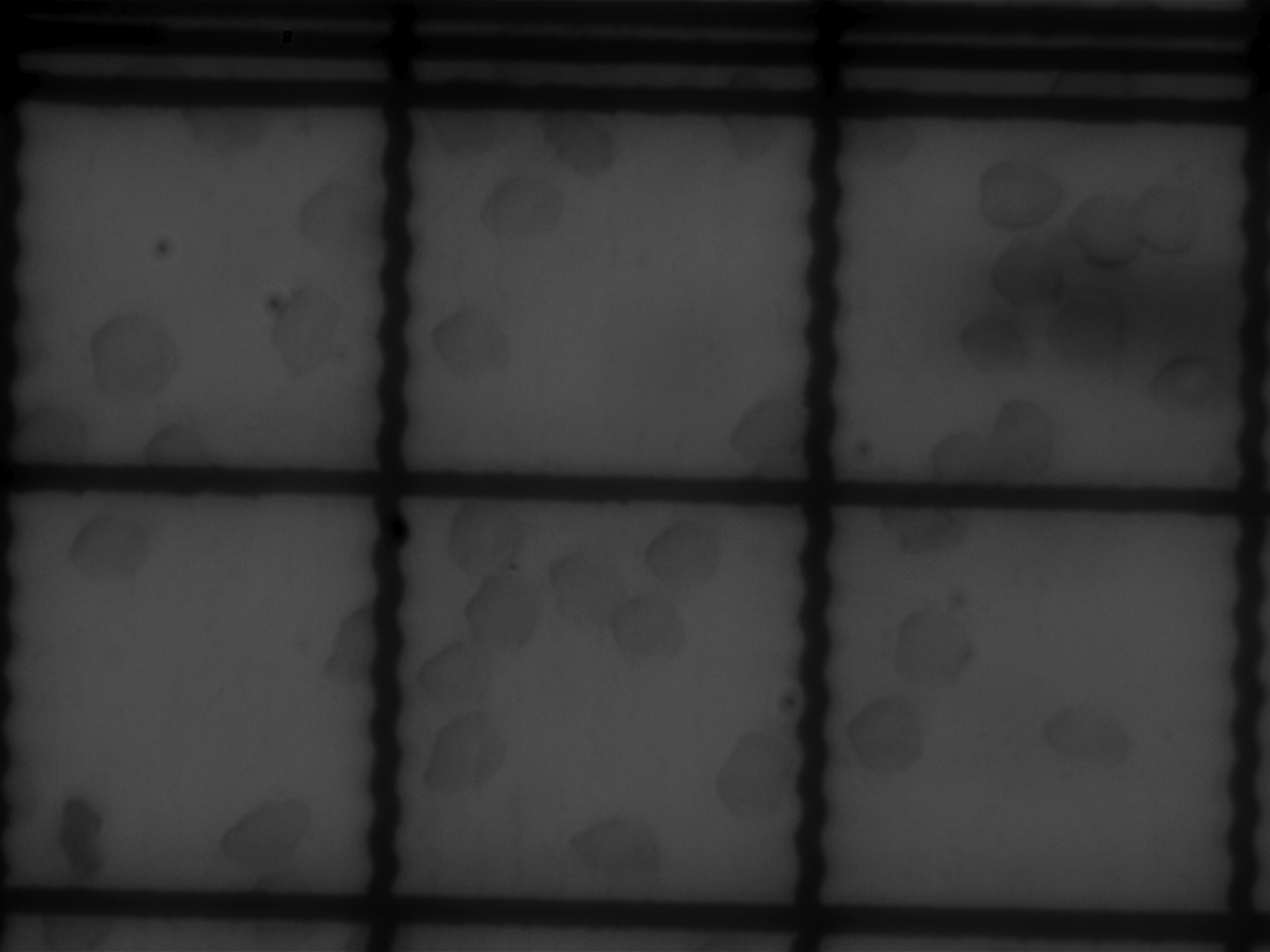

Supplement: S2 Image — (ZIP) [file pone.0226640.s005.zip › S2_imageseq_8_1.0_C1/C1-0002.png]

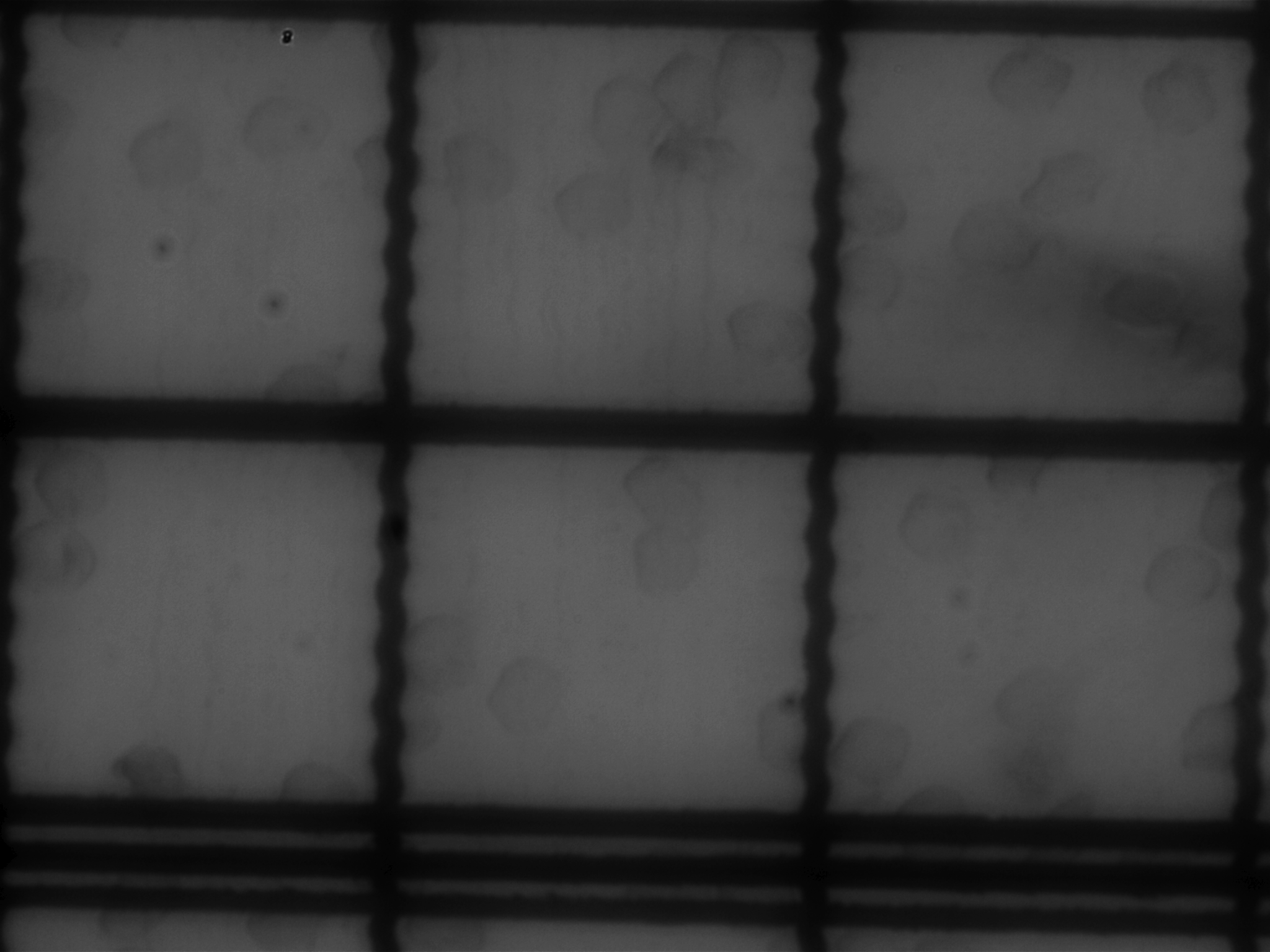

Supplement: S2 Image — (ZIP) [file pone.0226640.s005.zip › S2_imageseq_8_1.0_C1/C1-0001.png]

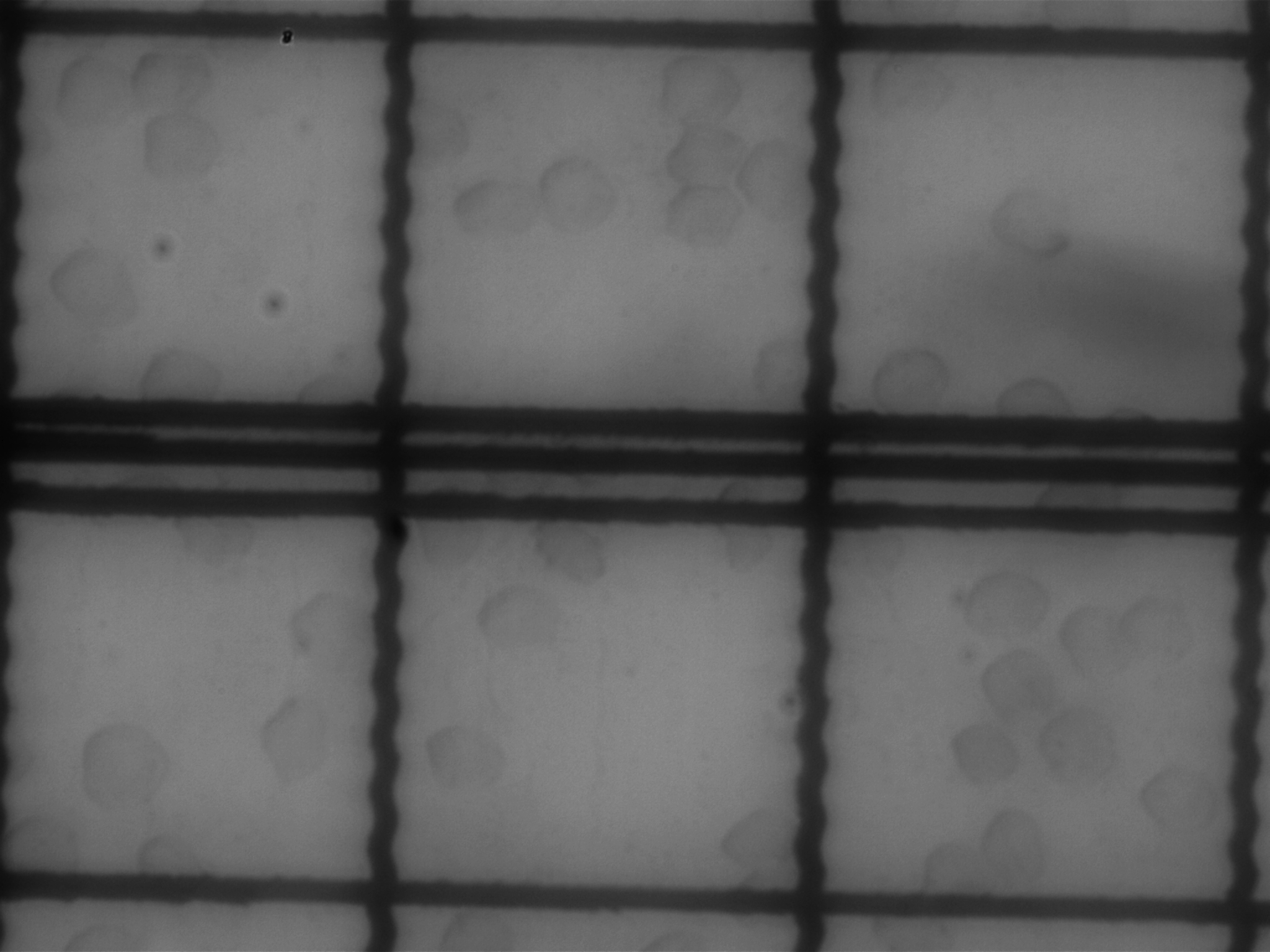

Supplement: S2 Image — (ZIP) [file pone.0226640.s005.zip › S2_imageseq_8_1.0_C1/C1-0003.png]

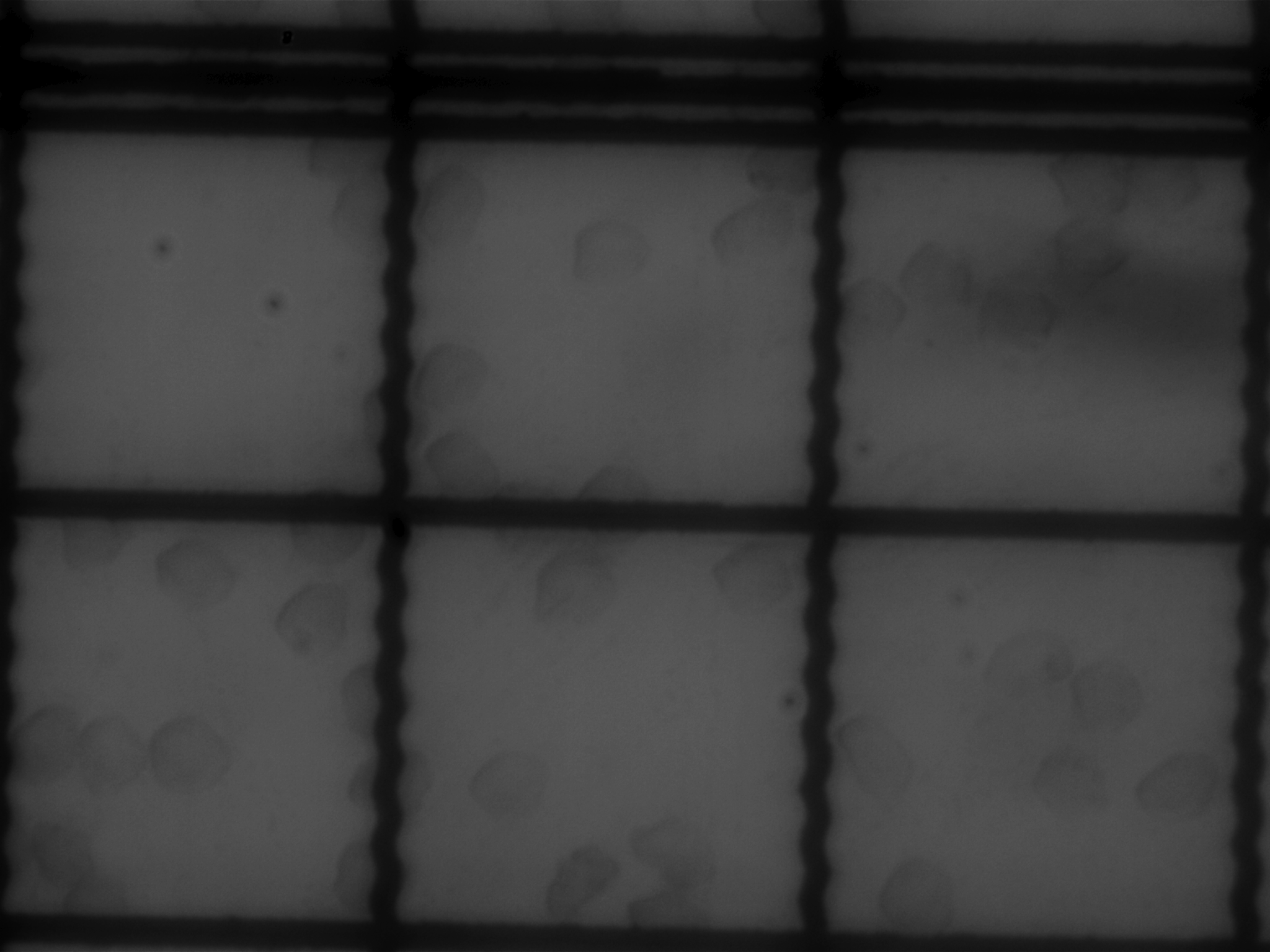

Supplement: S2 Image — (ZIP) [file pone.0226640.s005.zip › S2_imageseq_8_1.0_C1/C1-0004.png]

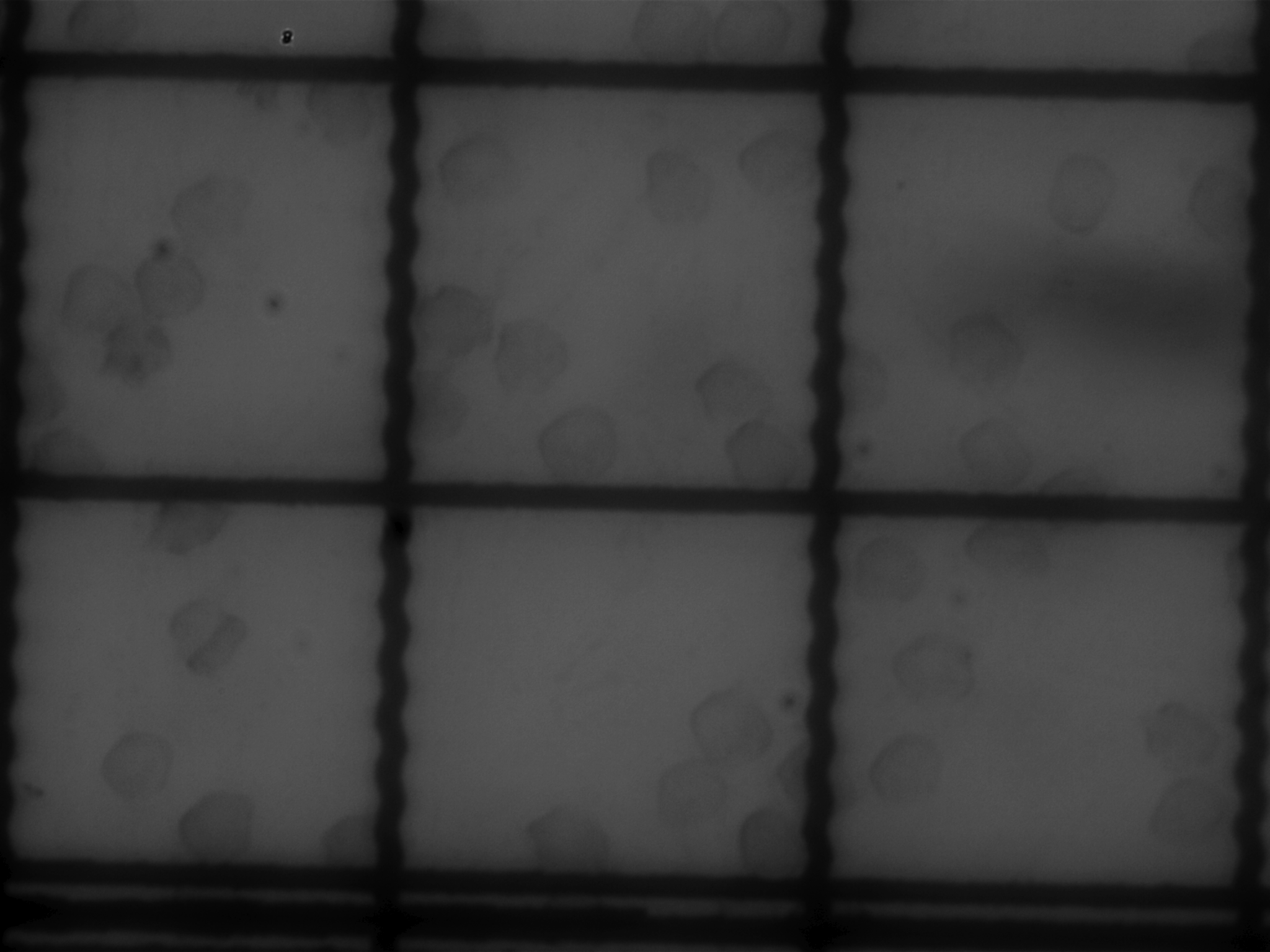

Supplement: S2 Image — (ZIP) [file pone.0226640.s005.zip › S2_imageseq_8_1.0_C1/C1-0005.png]

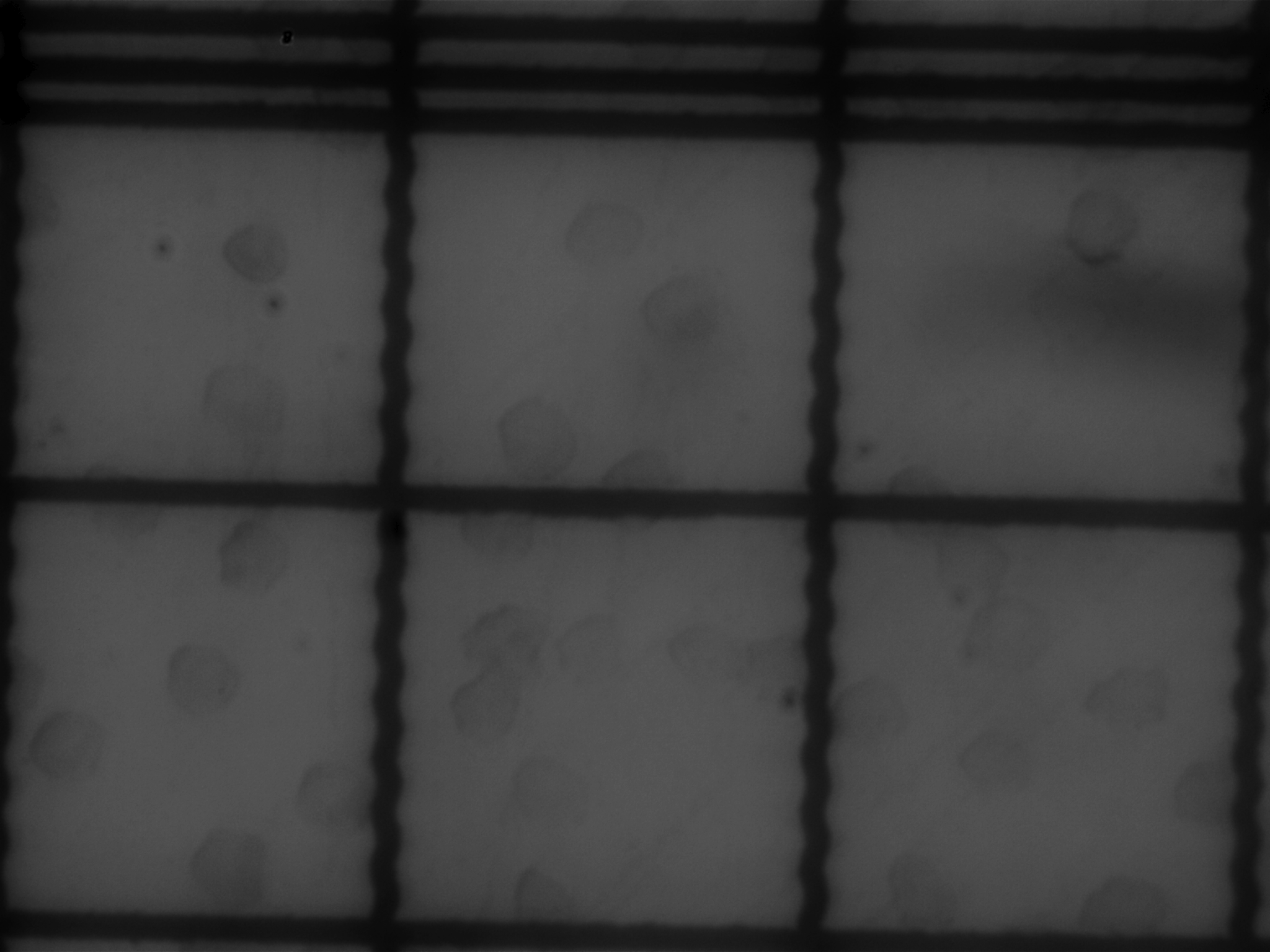

Supplement: S2 Image — (ZIP) [file pone.0226640.s005.zip › S2_imageseq_8_1.0_C1/C1-0006.png]

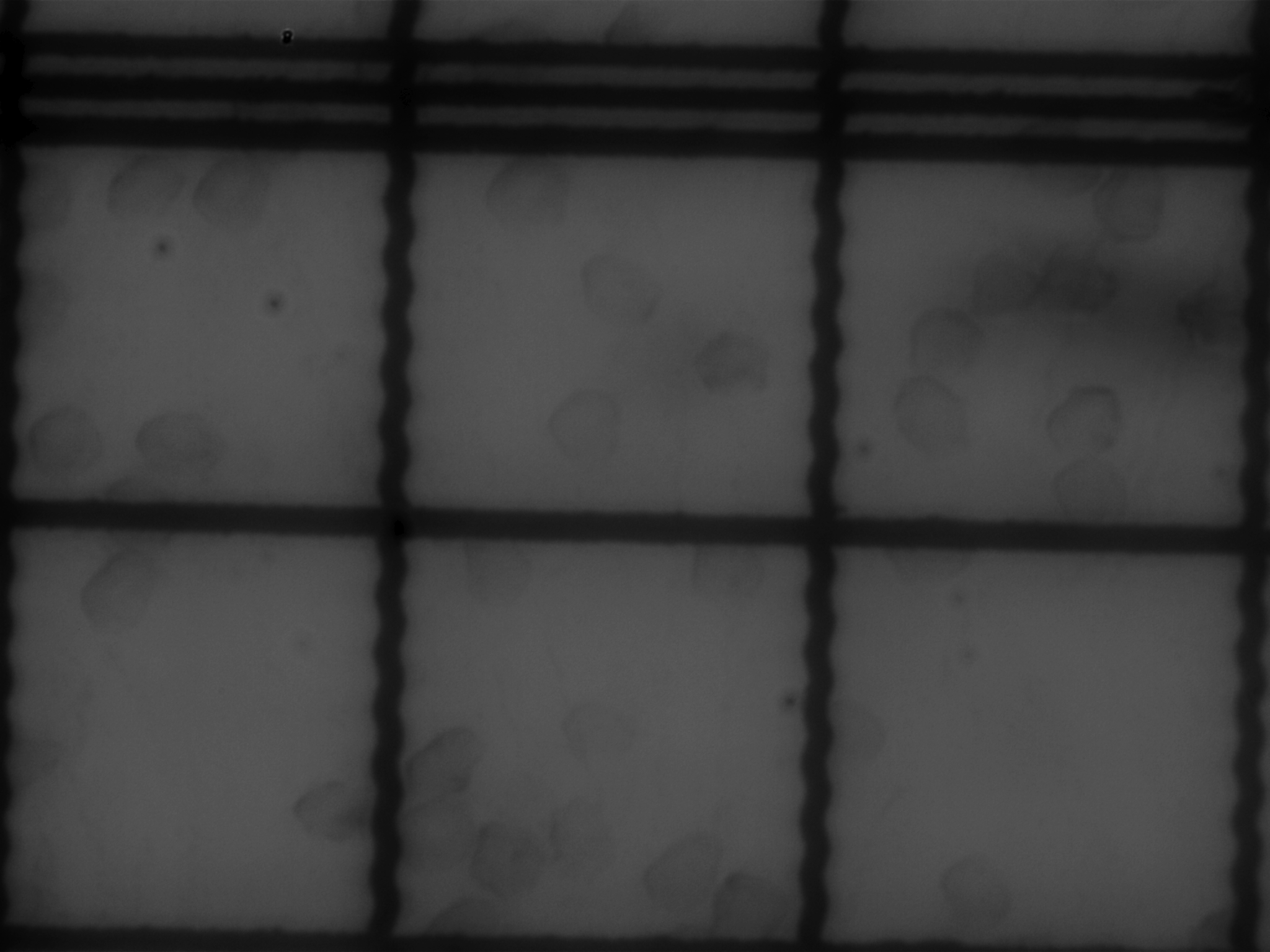

Supplement: S2 Image — (ZIP) [file pone.0226640.s005.zip › S2_imageseq_8_1.0_C1/C1-0007.png]

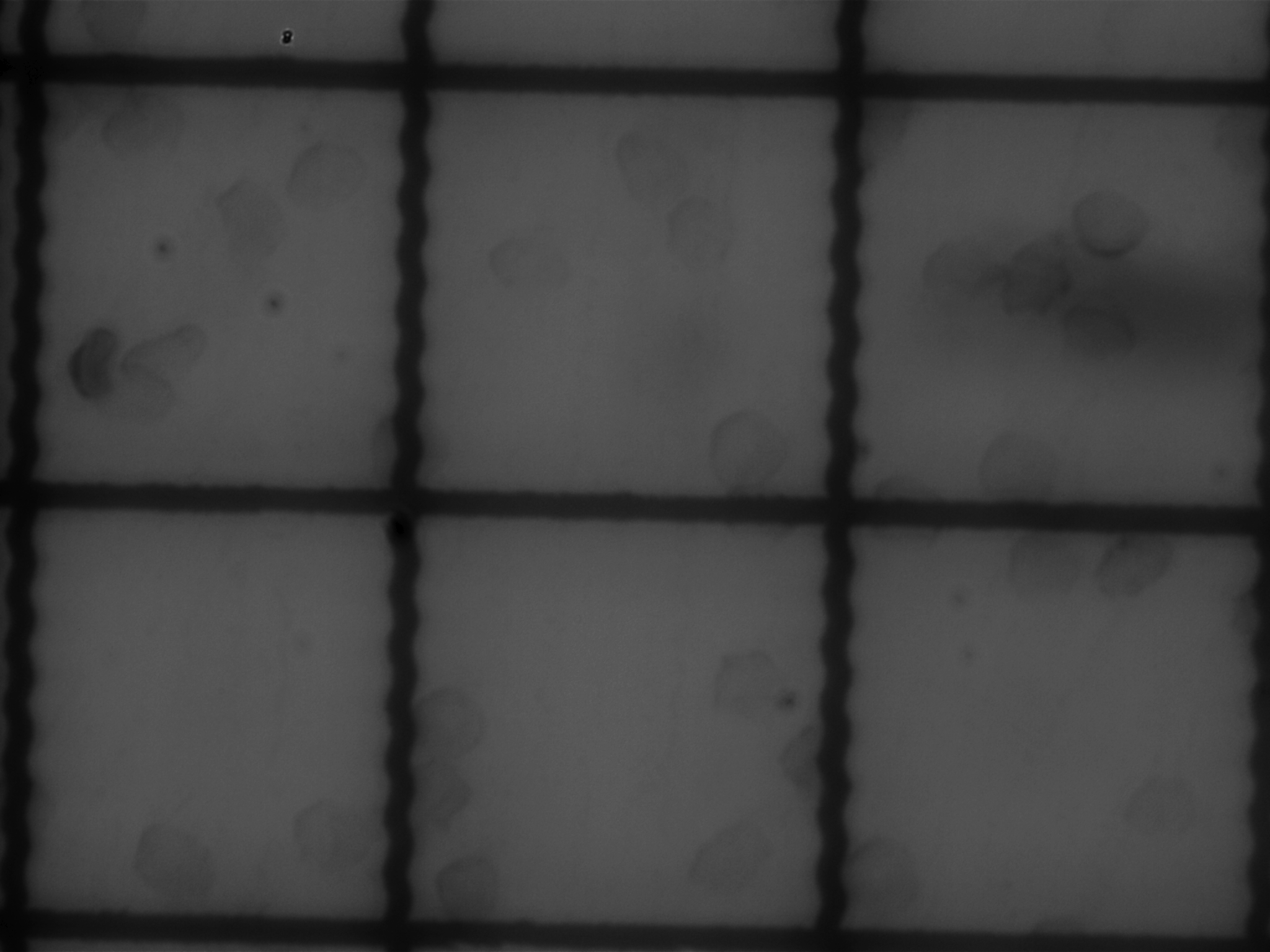

Supplement: S2 Image — (ZIP) [file pone.0226640.s005.zip › S2_imageseq_8_1.0_C1/C1-0008.png]

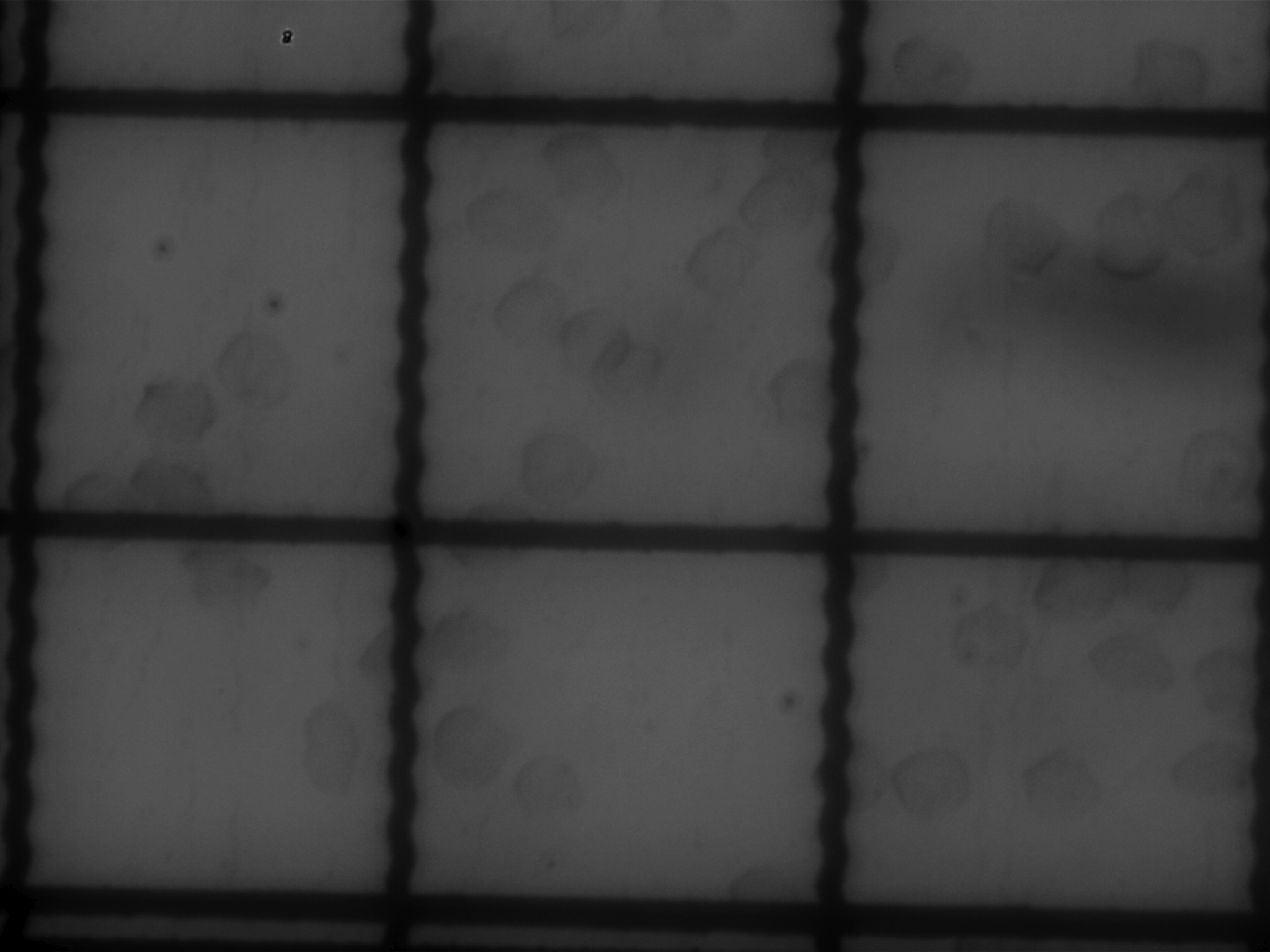

Supplement: S2 Image — (ZIP) [file pone.0226640.s005.zip › S2_imageseq_8_1.0_C1/C1-0009.png]

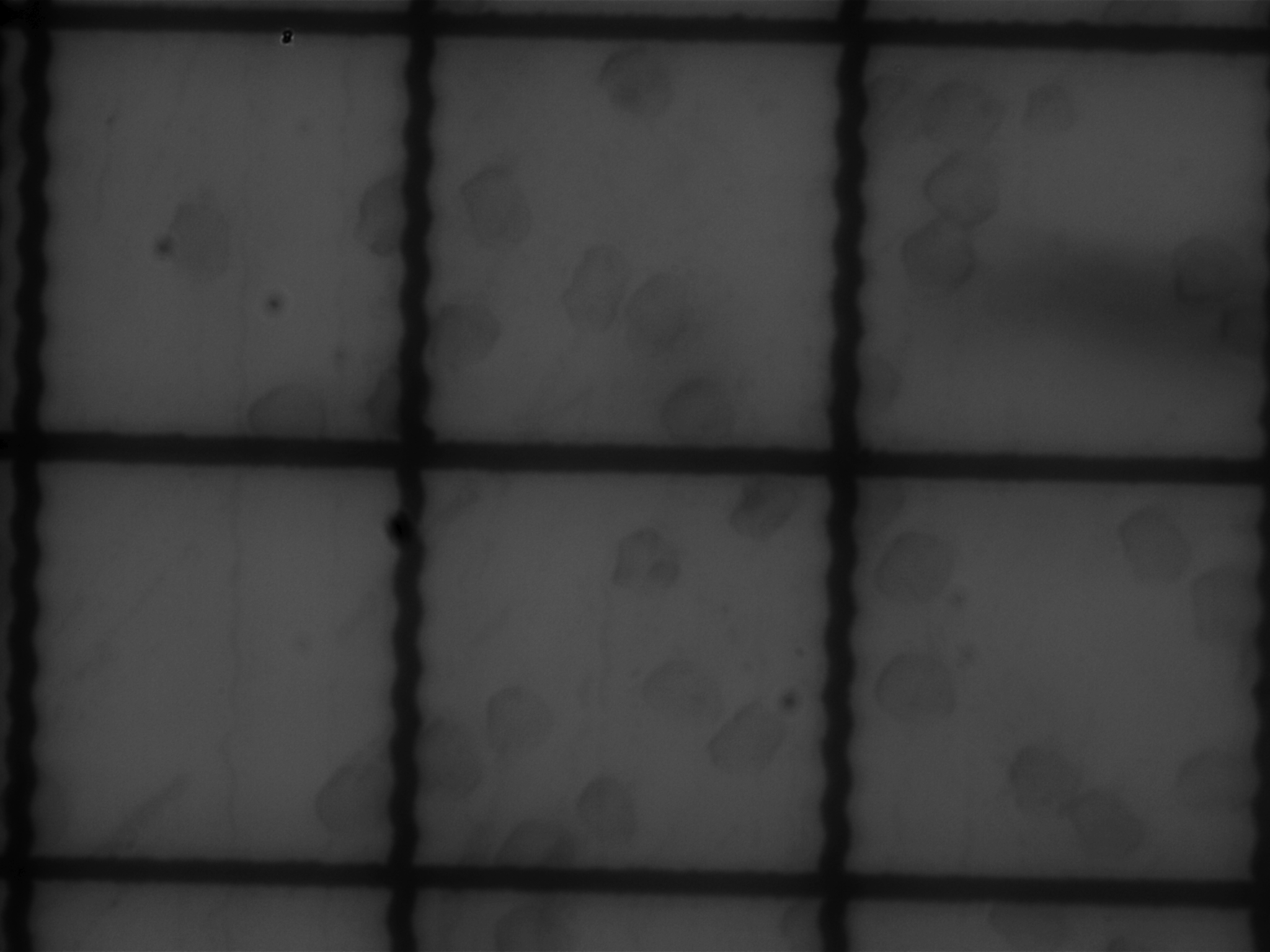

Supplement: S2 Image — (ZIP) [file pone.0226640.s005.zip › S2_imageseq_8_1.0_C1/C1-0010.png]

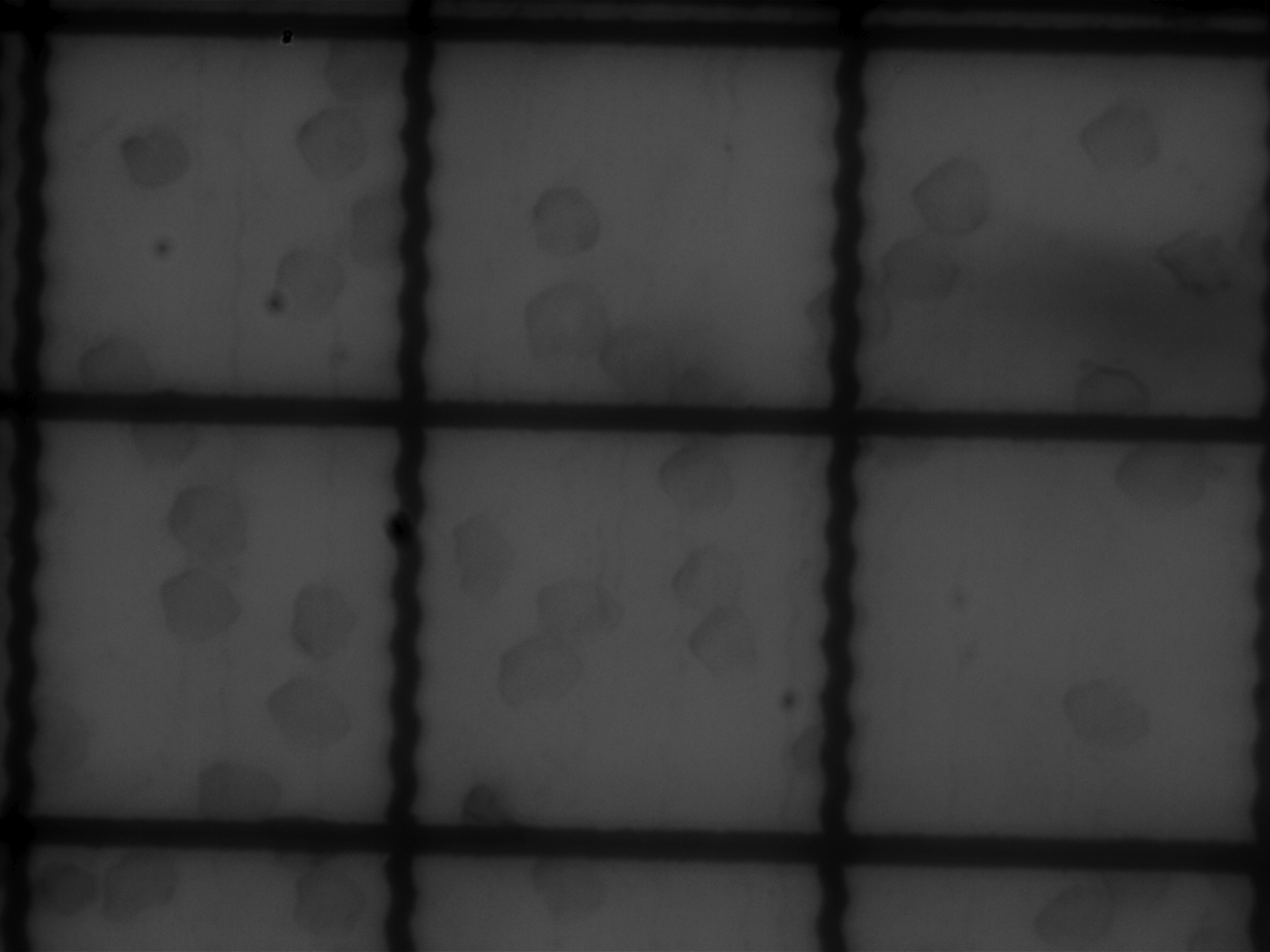

Supplement: S2 Image — (ZIP) [file pone.0226640.s005.zip › S2_imageseq_8_1.0_C1/C1-0011.png]

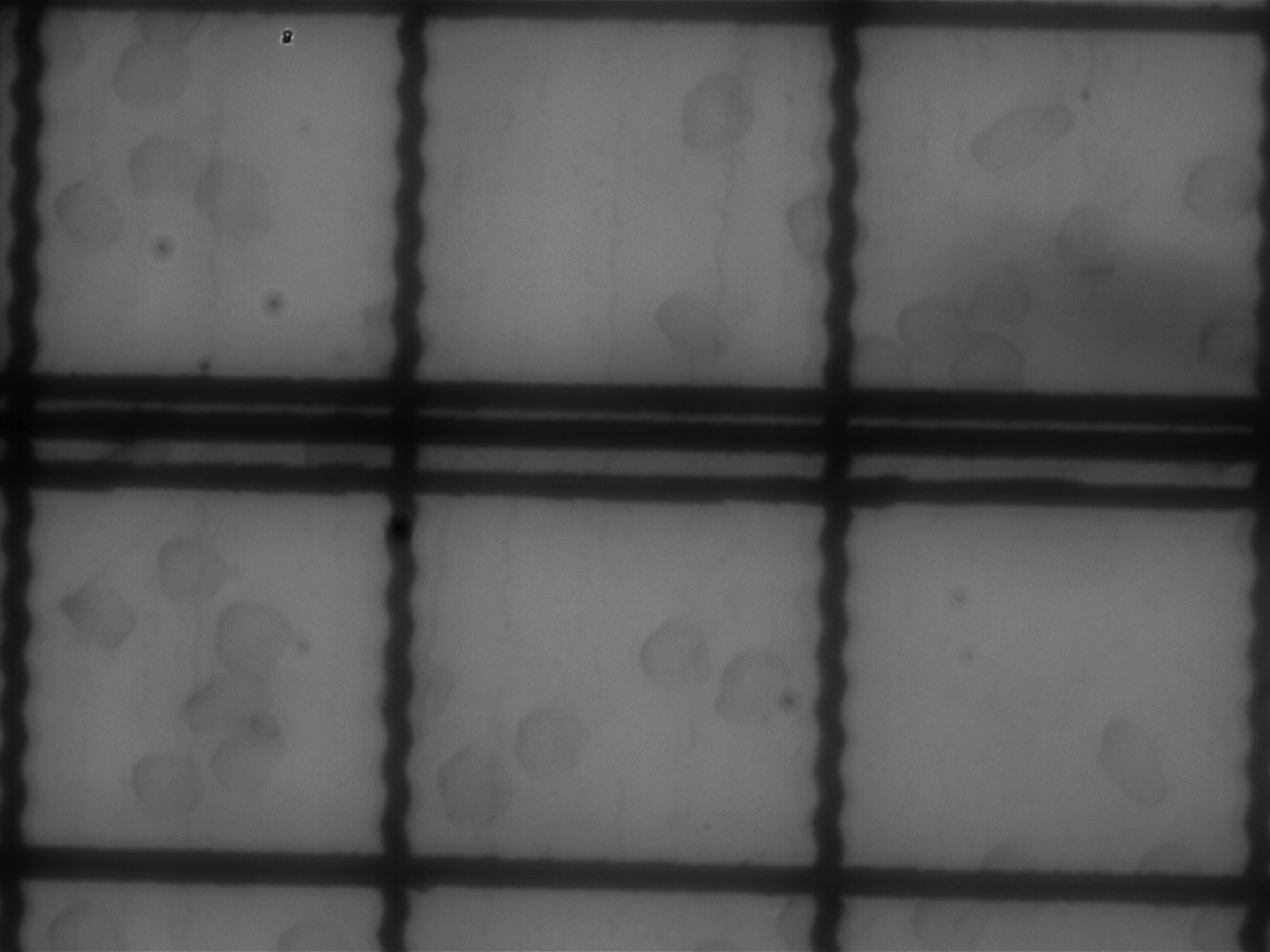

Supplement: S2 Image — (ZIP) [file pone.0226640.s005.zip › S2_imageseq_8_1.0_C1/C1-0012.png]

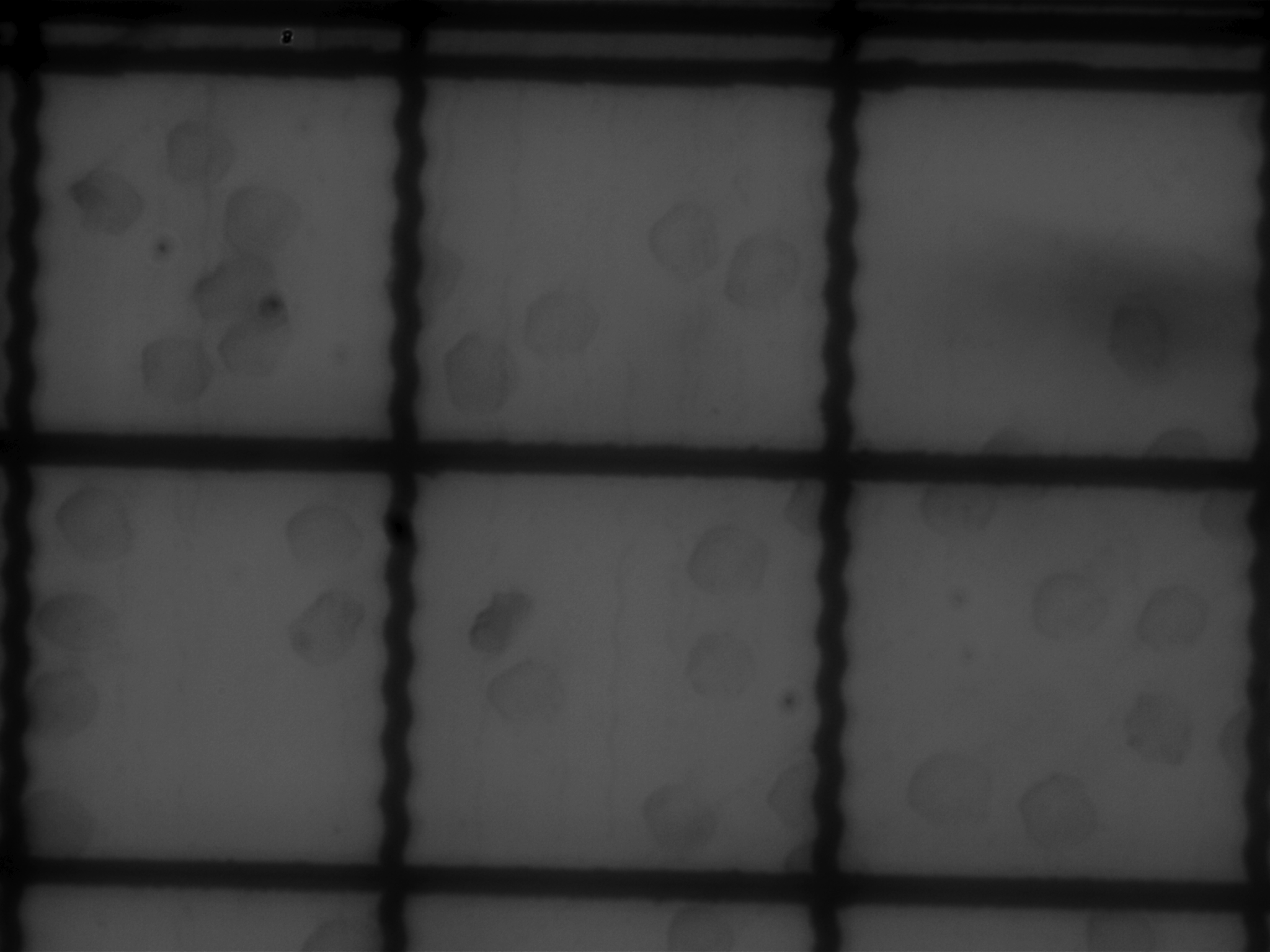

Supplement: S2 Image — (ZIP) [file pone.0226640.s005.zip › S2_imageseq_8_1.0_C1/C1-0013.png]

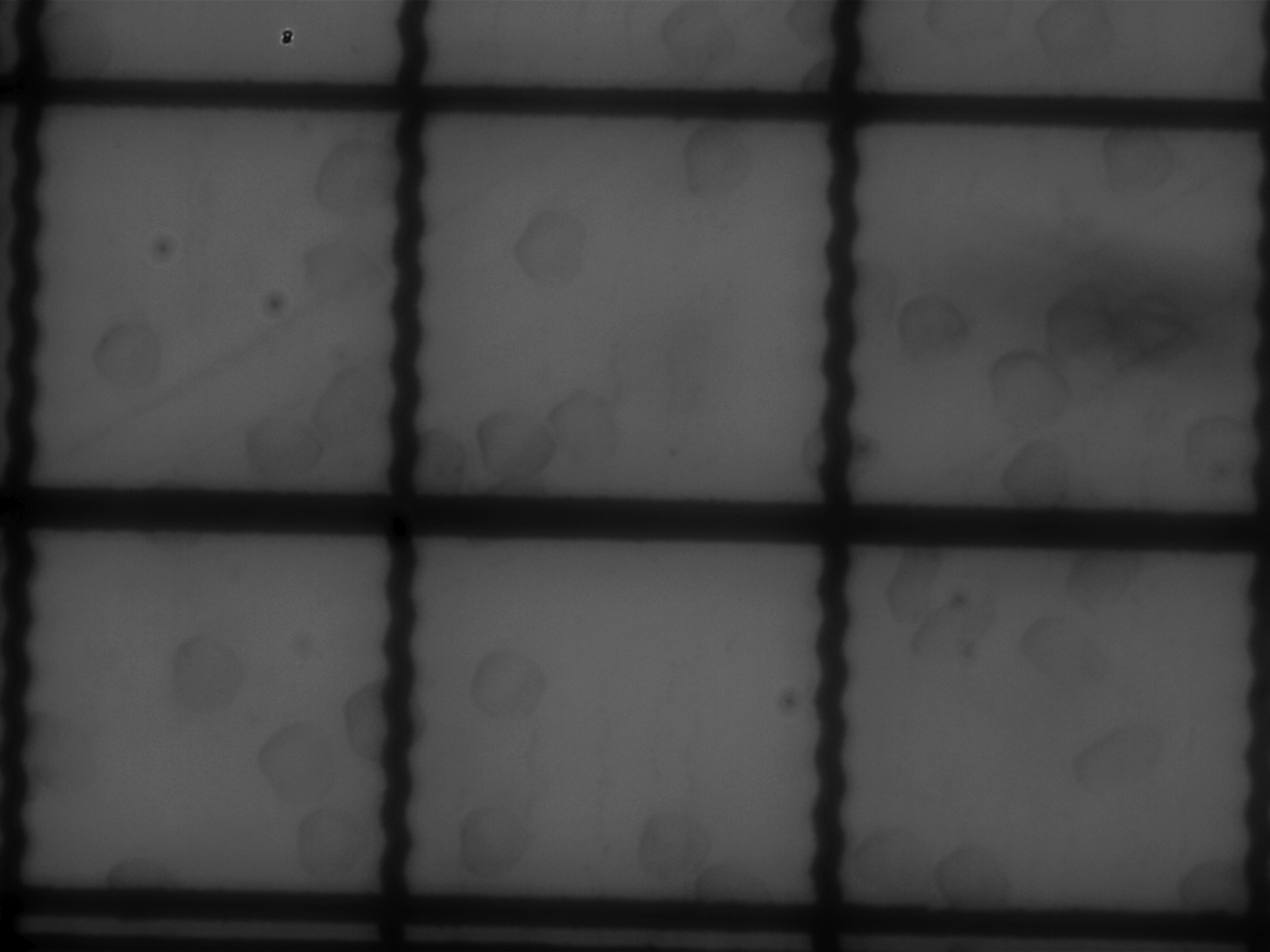

Supplement: S2 Image — (ZIP) [file pone.0226640.s005.zip › S2_imageseq_8_1.0_C1/C1-0014.png]

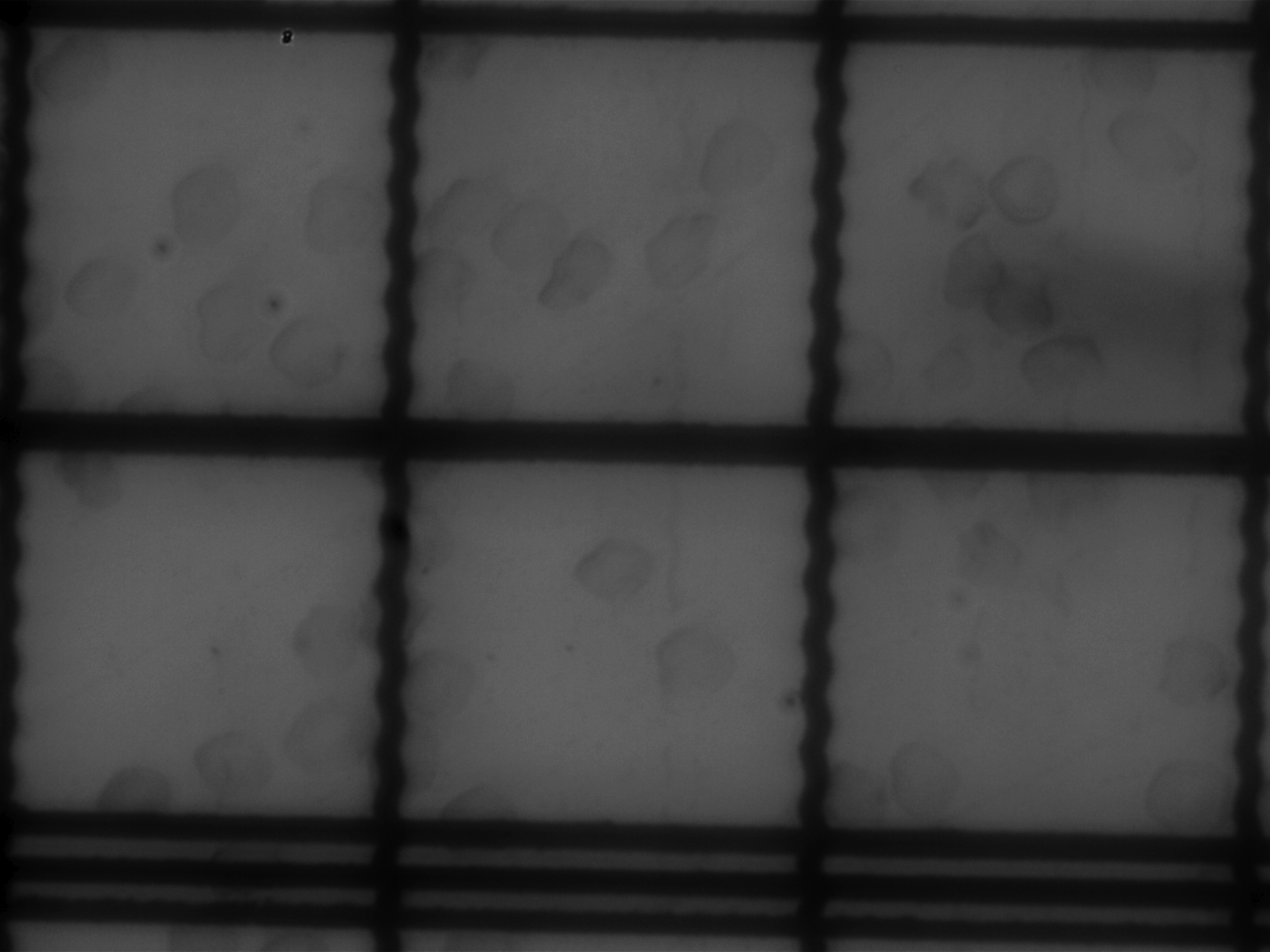

Supplement: S2 Image — (ZIP) [file pone.0226640.s005.zip › S2_imageseq_8_1.0_C1/C1-0015.png]

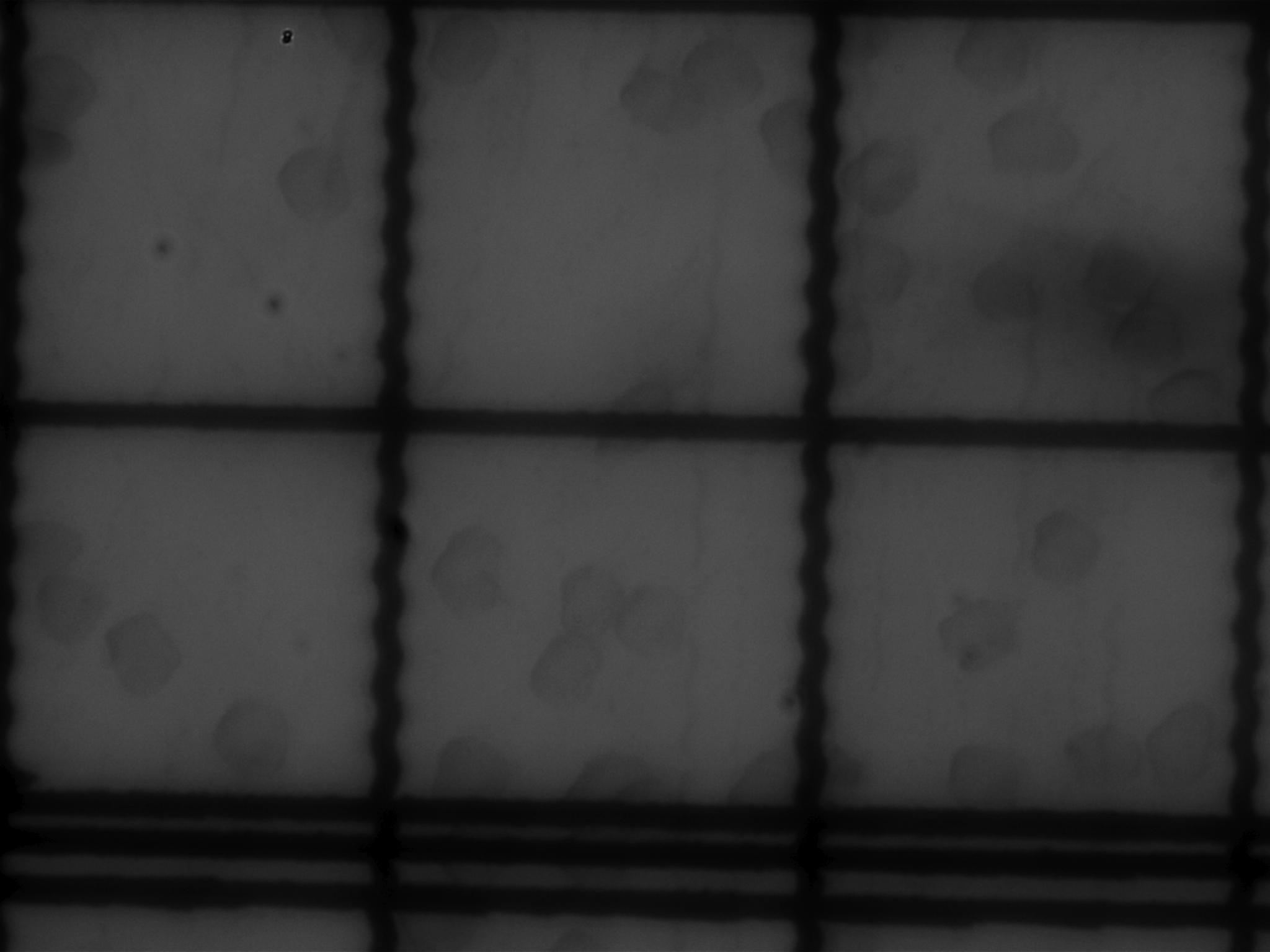

Supplement: S2 Image — (ZIP) [file pone.0226640.s005.zip › S2_imageseq_8_1.0_C1/C1-0016.png]

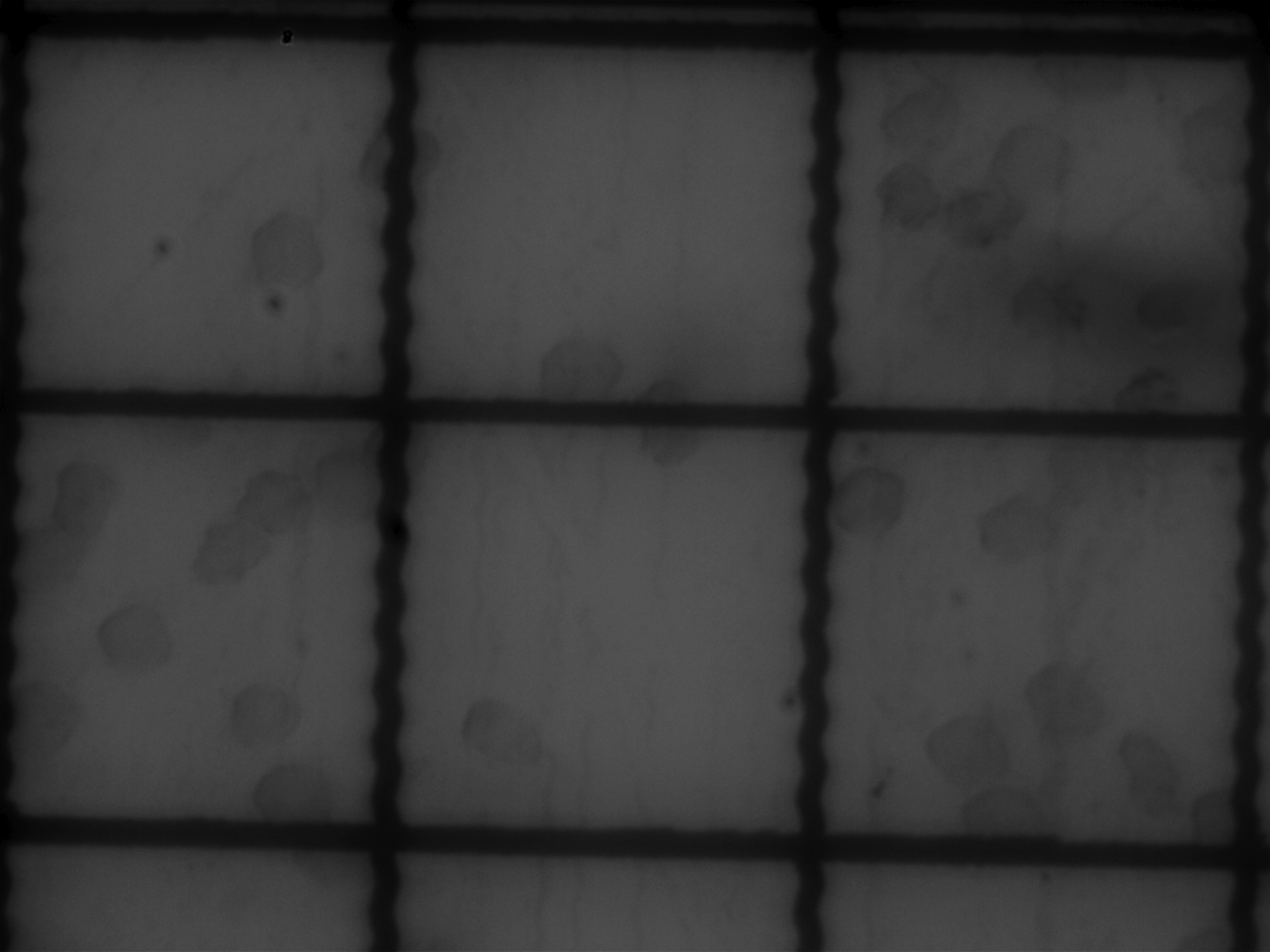

Supplement: S2 Image — (ZIP) [file pone.0226640.s005.zip › S2_imageseq_8_1.0_C1/C1-0017.png]

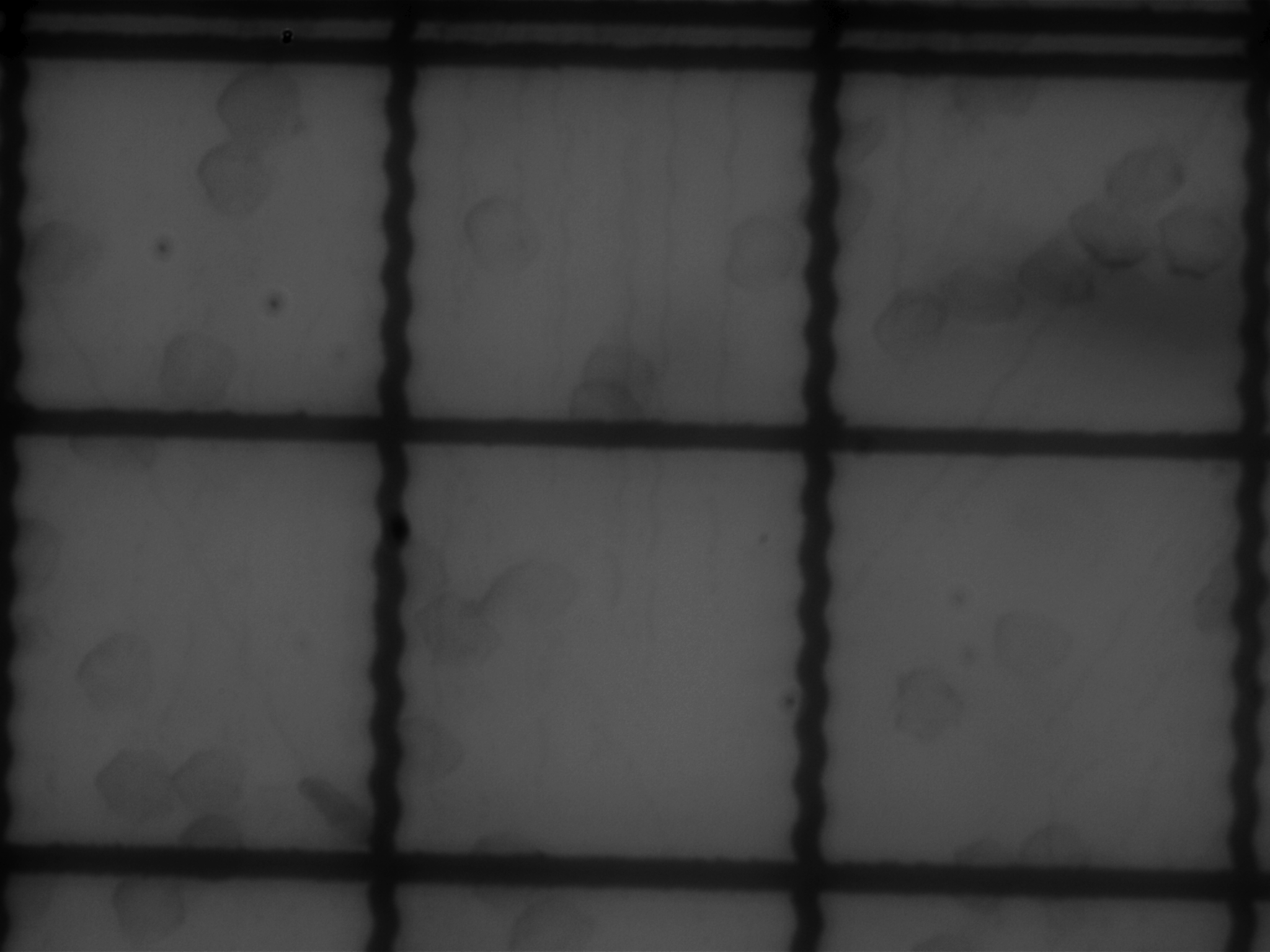

Supplement: S2 Image — (ZIP) [file pone.0226640.s005.zip › S2_imageseq_8_1.0_C1/C1-0018.png]

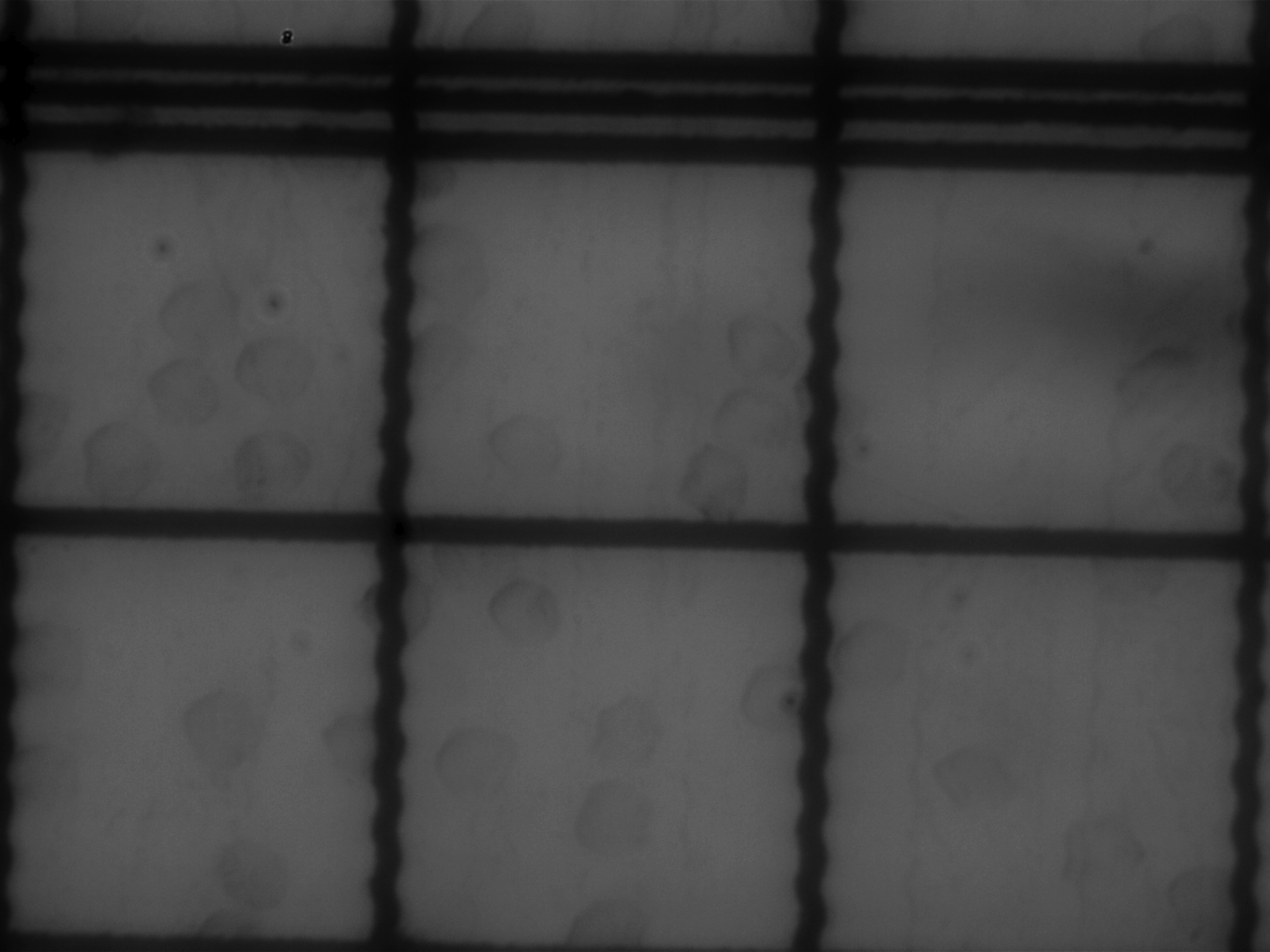

Supplement: S2 Image — (ZIP) [file pone.0226640.s005.zip › S2_imageseq_8_1.0_C1/C1-0019.png]

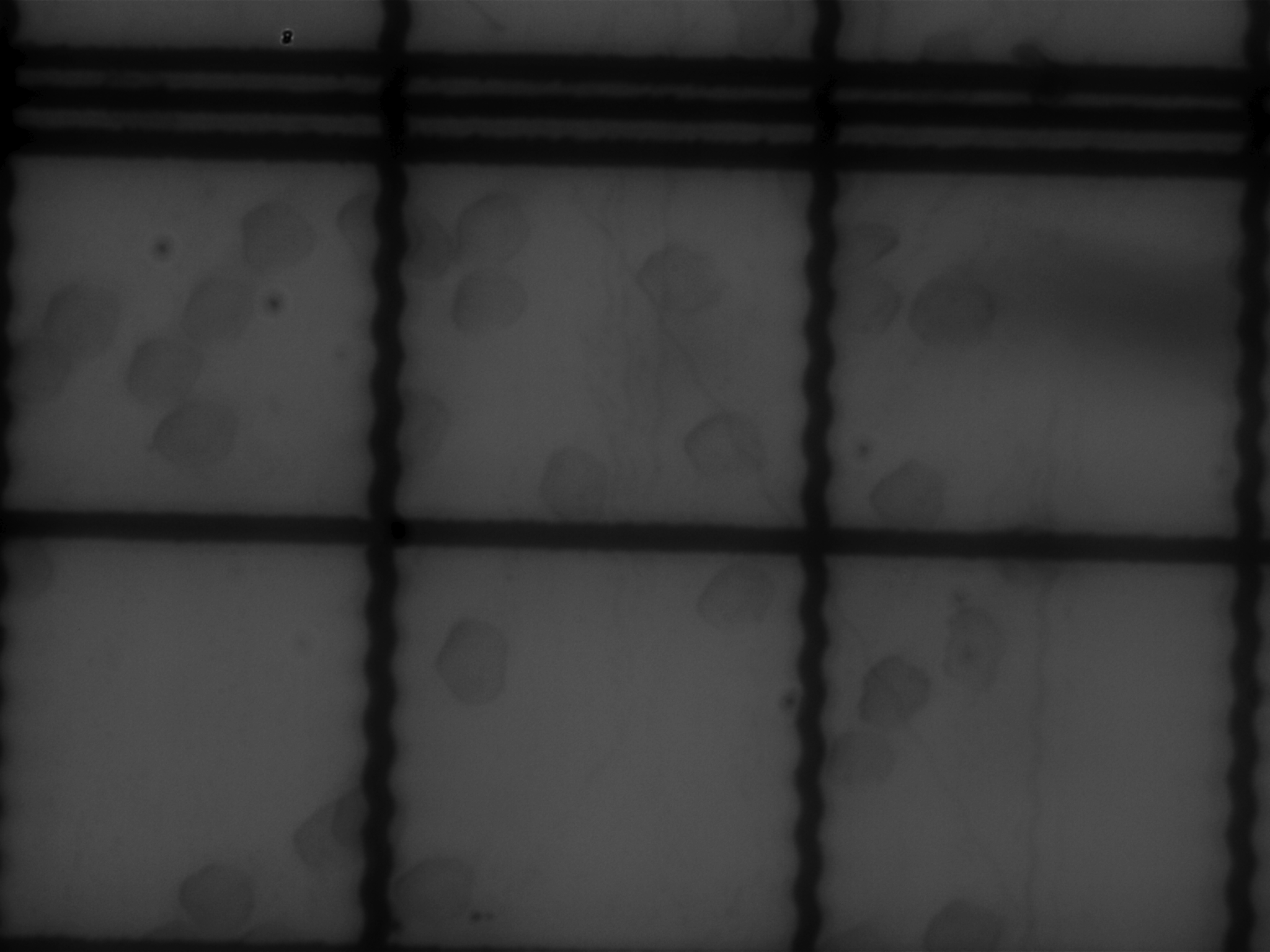

Supplement: S2 Image — (ZIP) [file pone.0226640.s005.zip › S2_imageseq_8_1.0_C1/C1-0020.png]

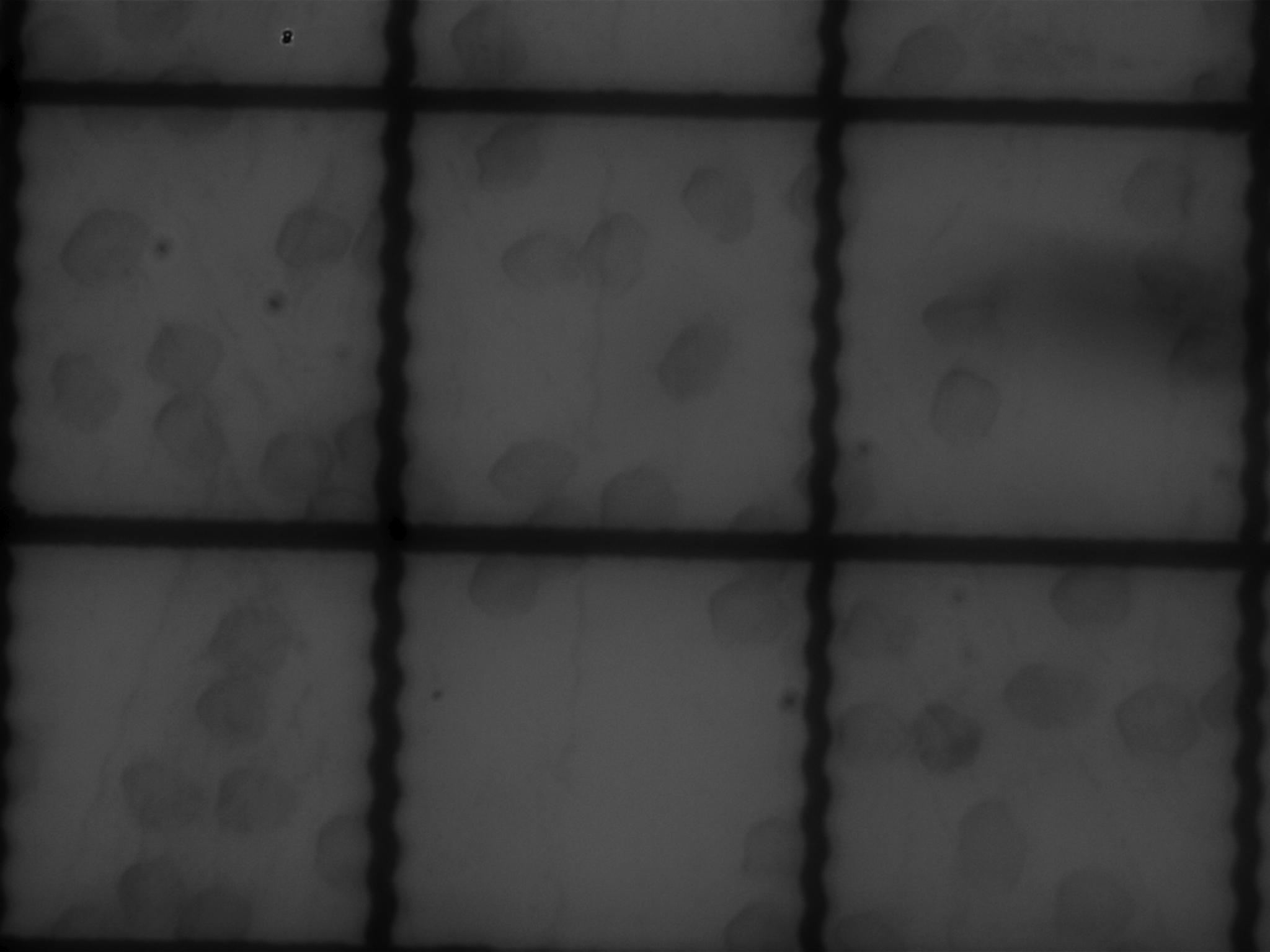

Supplement: S2 Image — (ZIP) [file pone.0226640.s005.zip › S2_imageseq_8_1.0_C1/C1-0021.png]

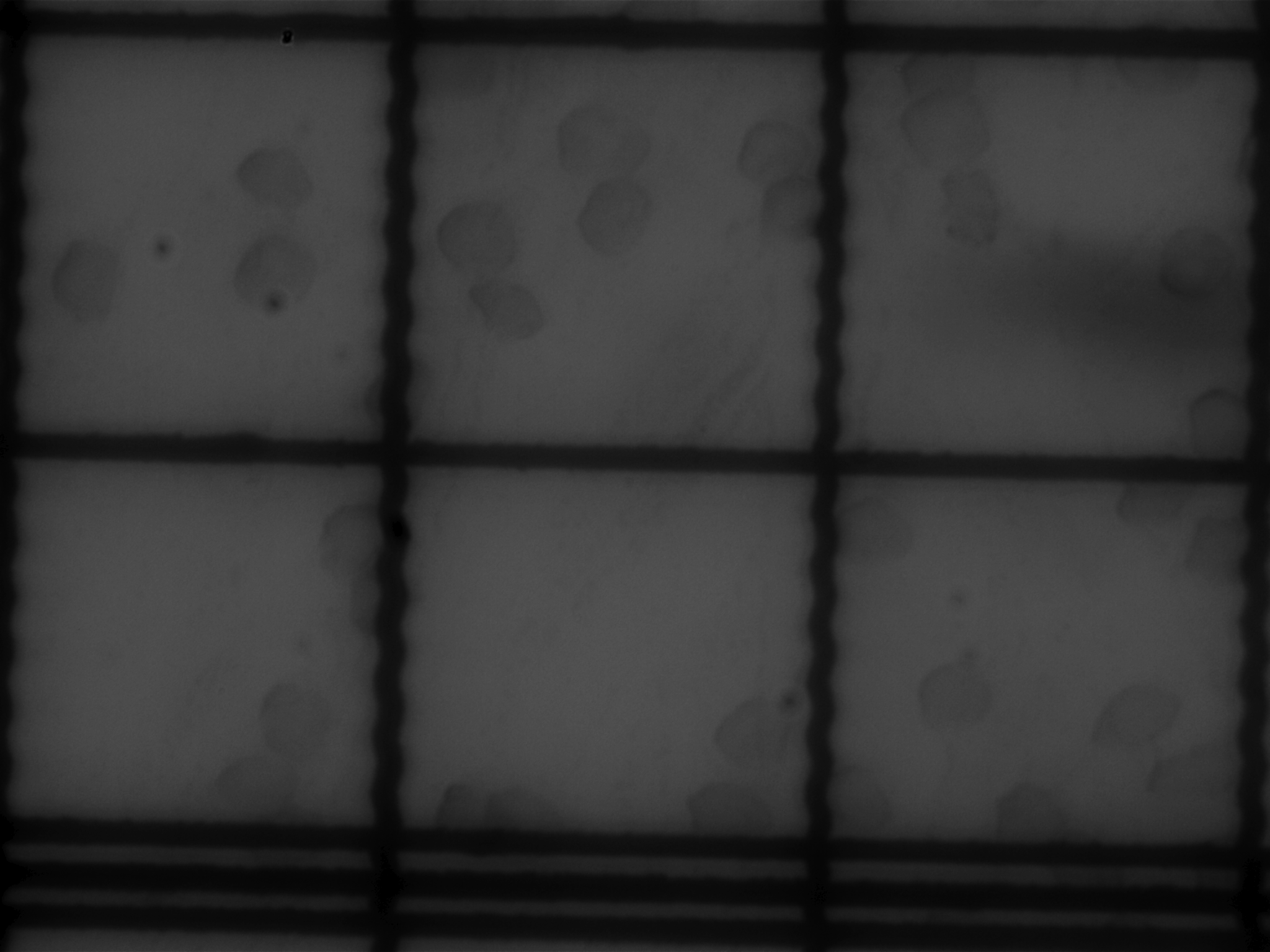

Supplement: S2 Image — (ZIP) [file pone.0226640.s005.zip › S2_imageseq_8_1.0_C1/C1-0022.png]

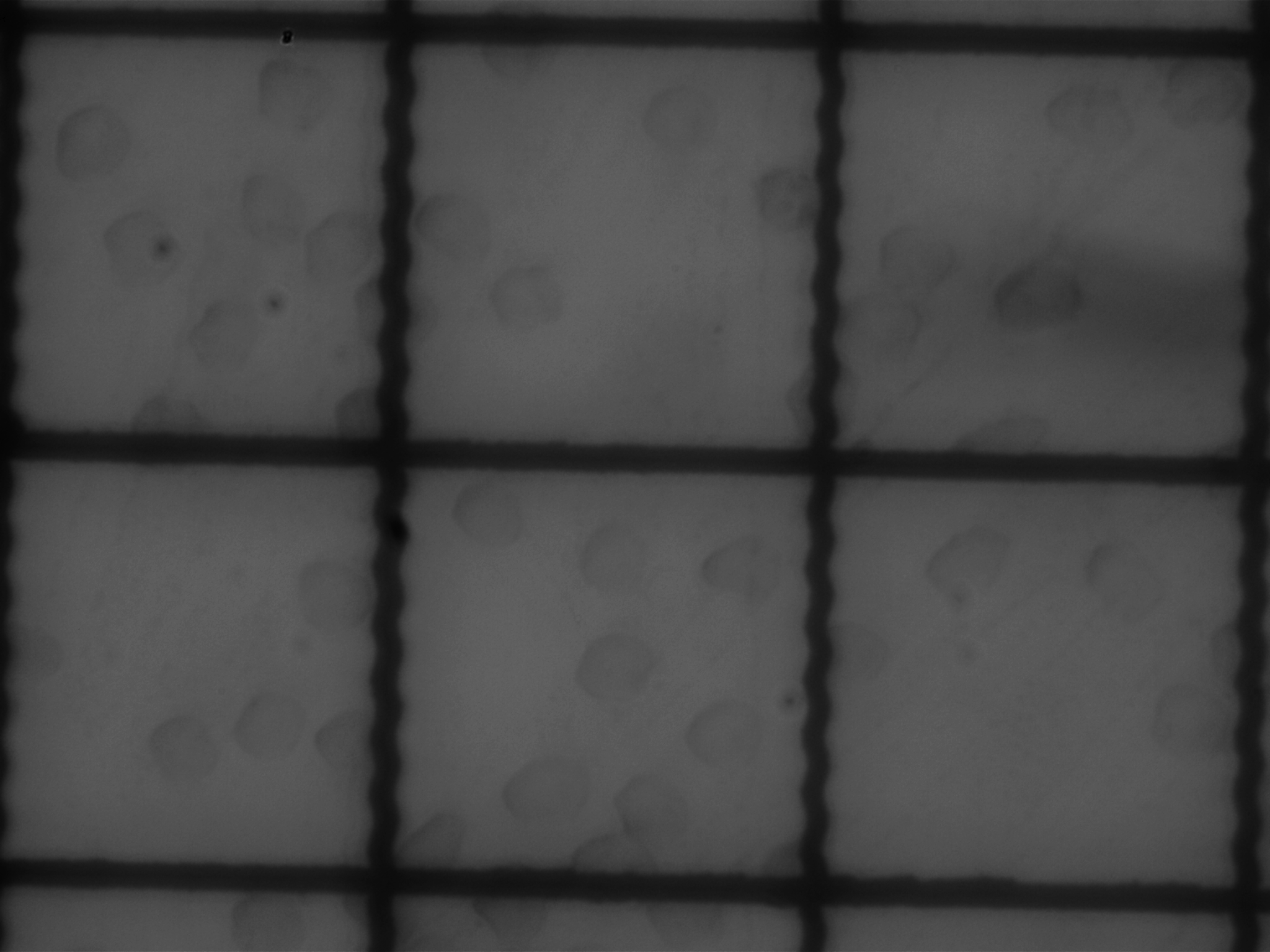

Supplement: S2 Image — (ZIP) [file pone.0226640.s005.zip › S2_imageseq_8_1.0_C1/C1-0023.png]

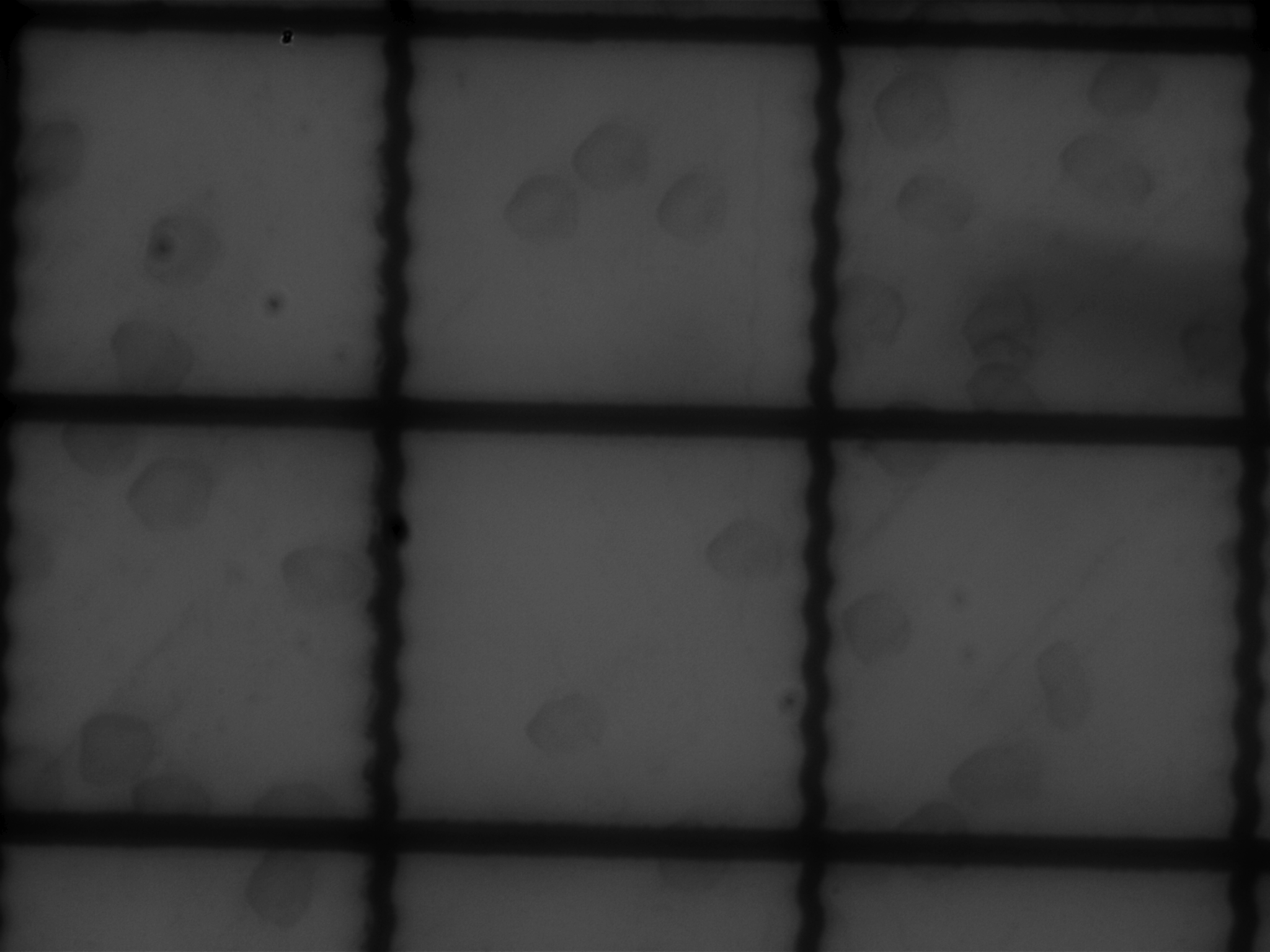

Supplement: S2 Image — (ZIP) [file pone.0226640.s005.zip › S2_imageseq_8_1.0_C1/C1-0024.png]

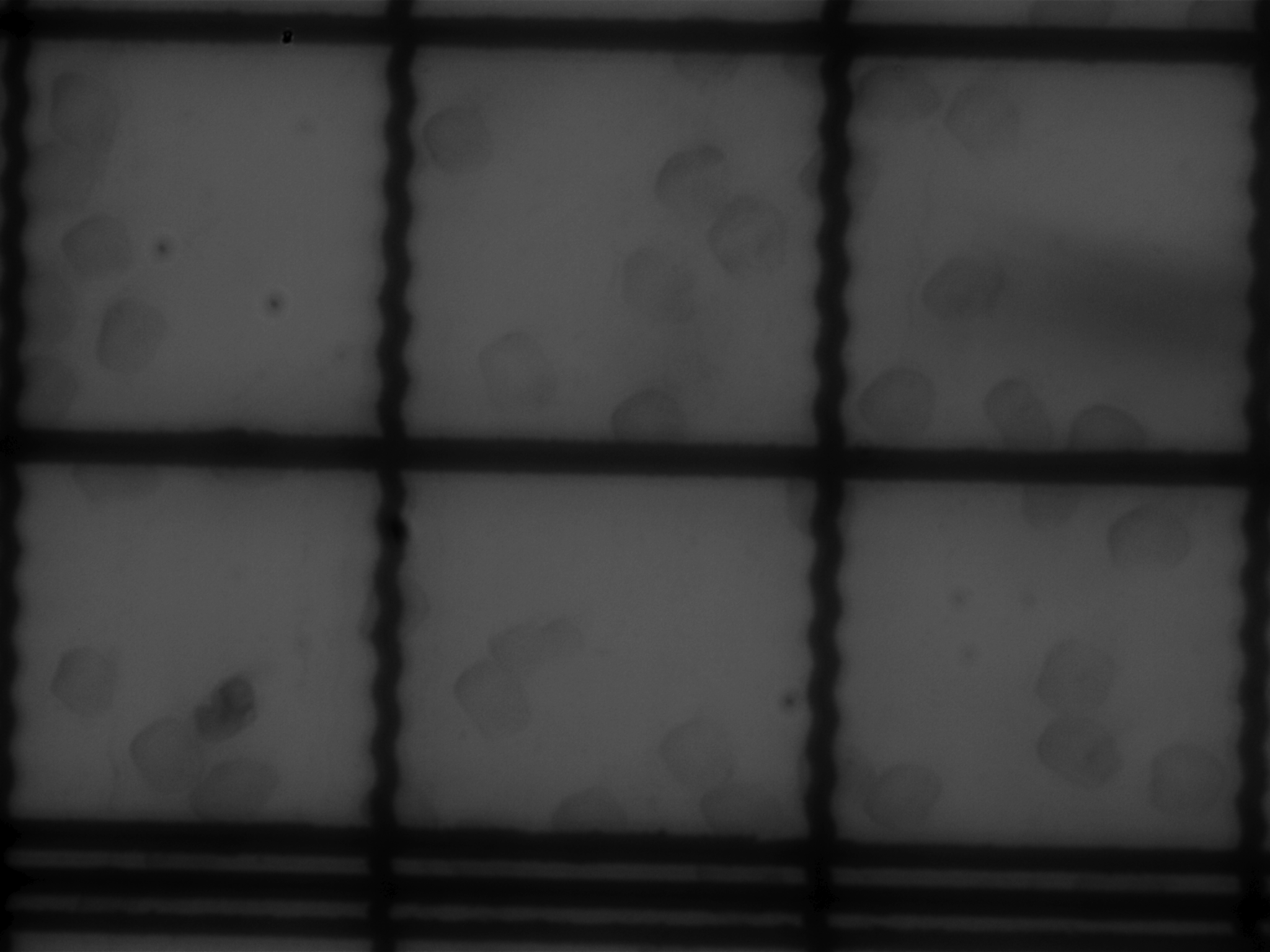

Supplement: S2 Image — (ZIP) [file pone.0226640.s005.zip › S2_imageseq_8_1.0_C1/C1-0025.png]

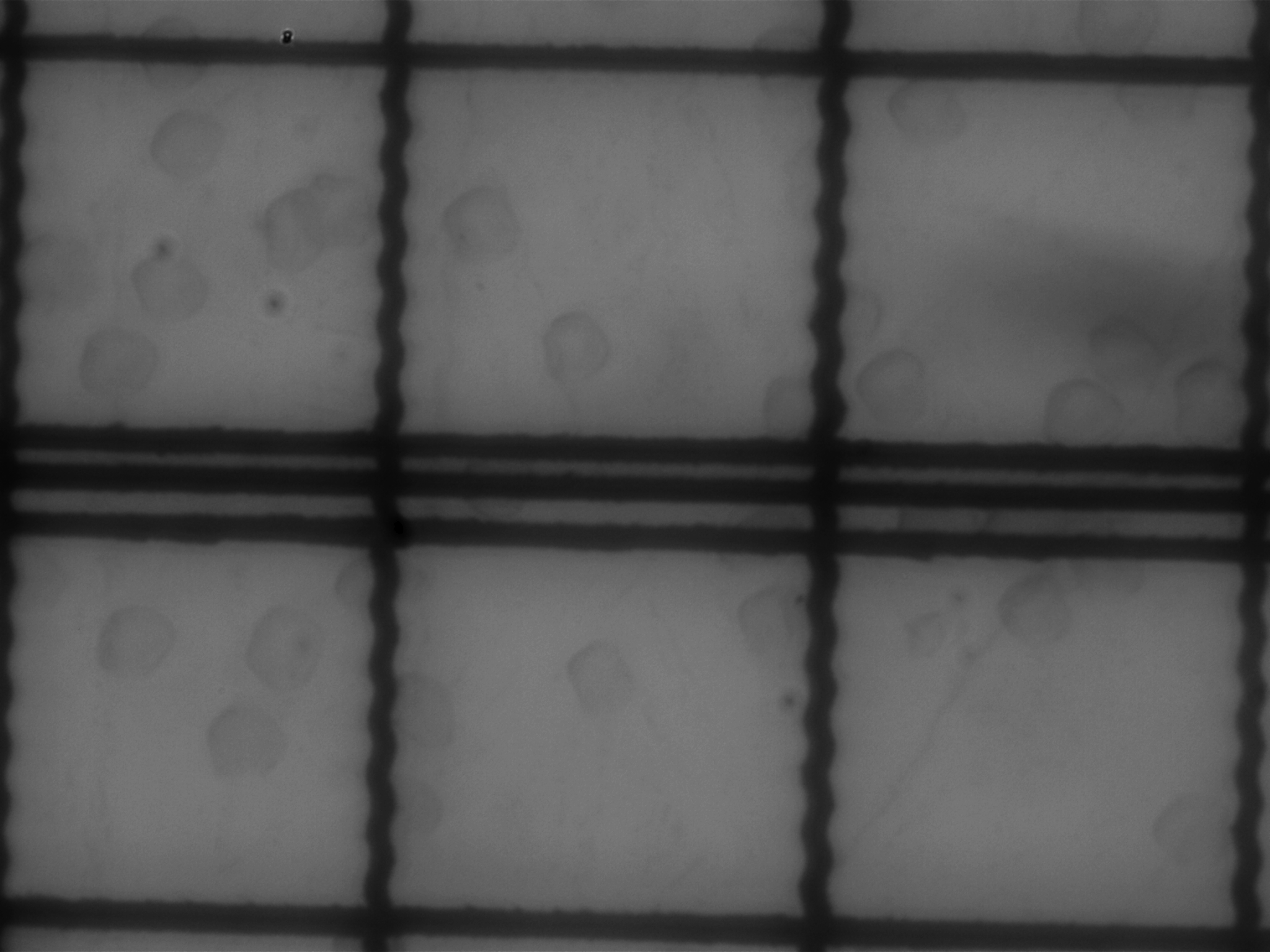

Supplement: S2 Image — (ZIP) [file pone.0226640.s005.zip › S2_imageseq_8_1.0_C1/C1-0026.png]

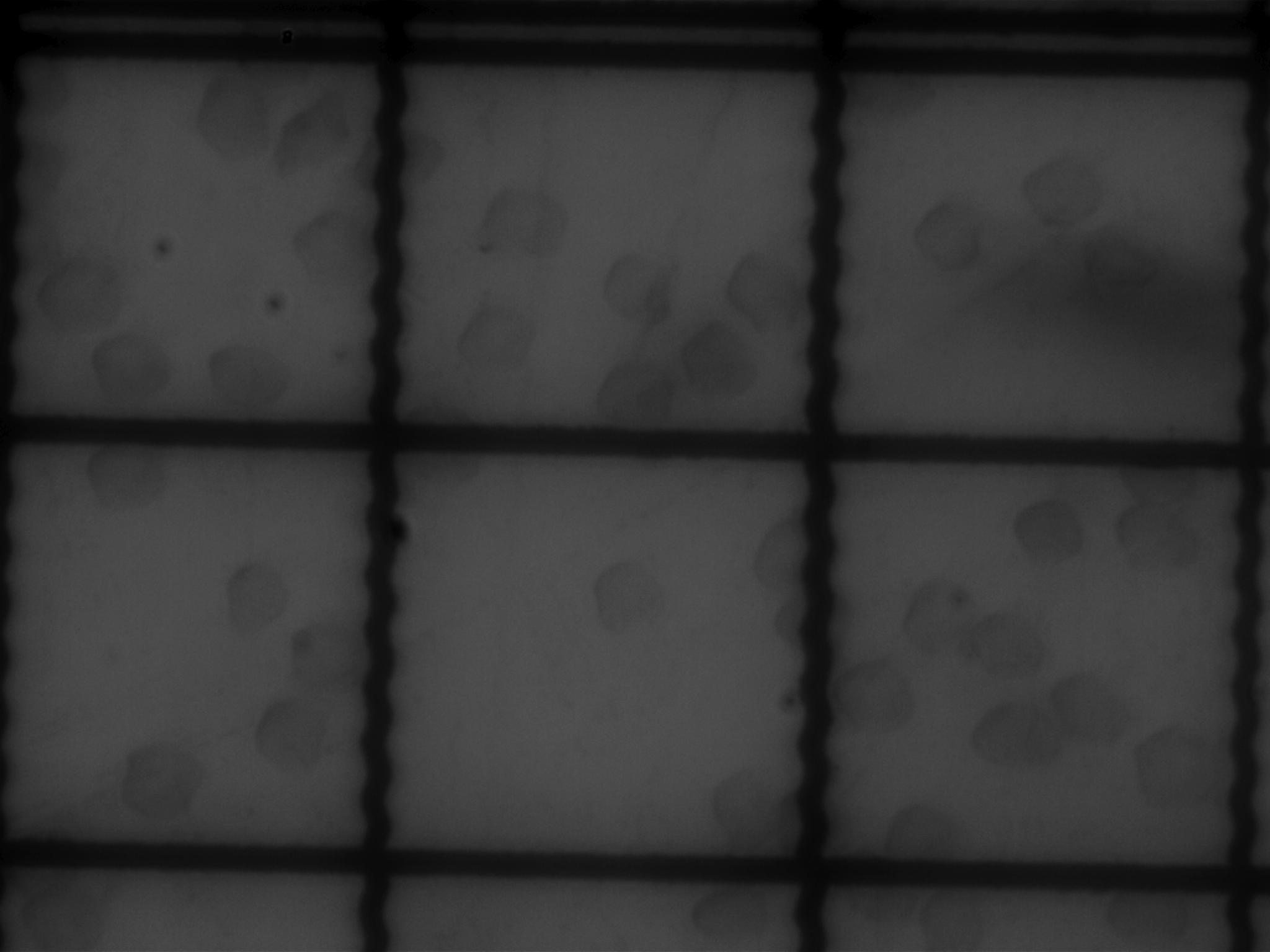

Supplement: S2 Image — (ZIP) [file pone.0226640.s005.zip › S2_imageseq_8_1.0_C1/C1-0027.png]

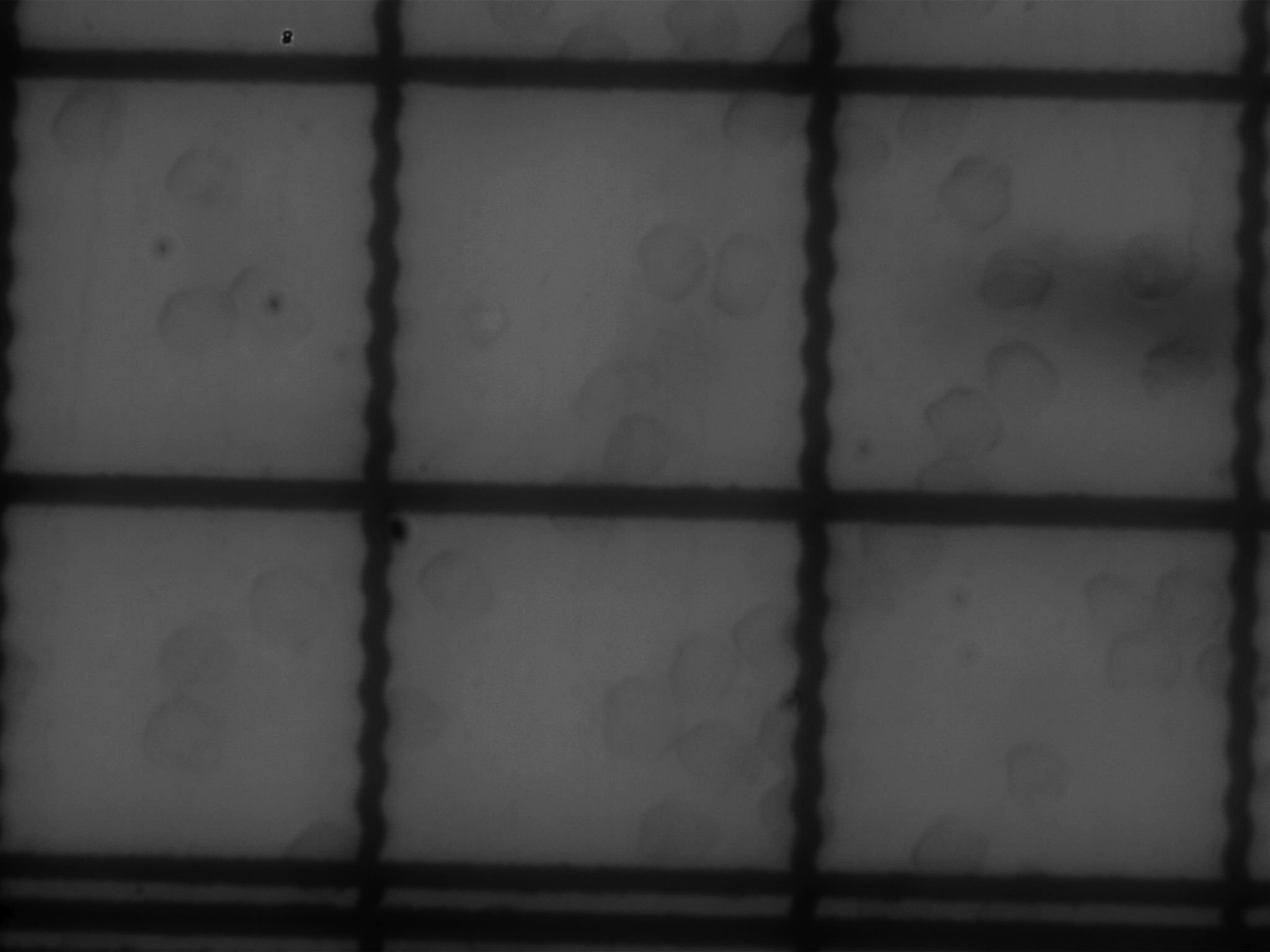

Supplement: S2 Image — (ZIP) [file pone.0226640.s005.zip › S2_imageseq_8_1.0_C1/C1-0028.png]

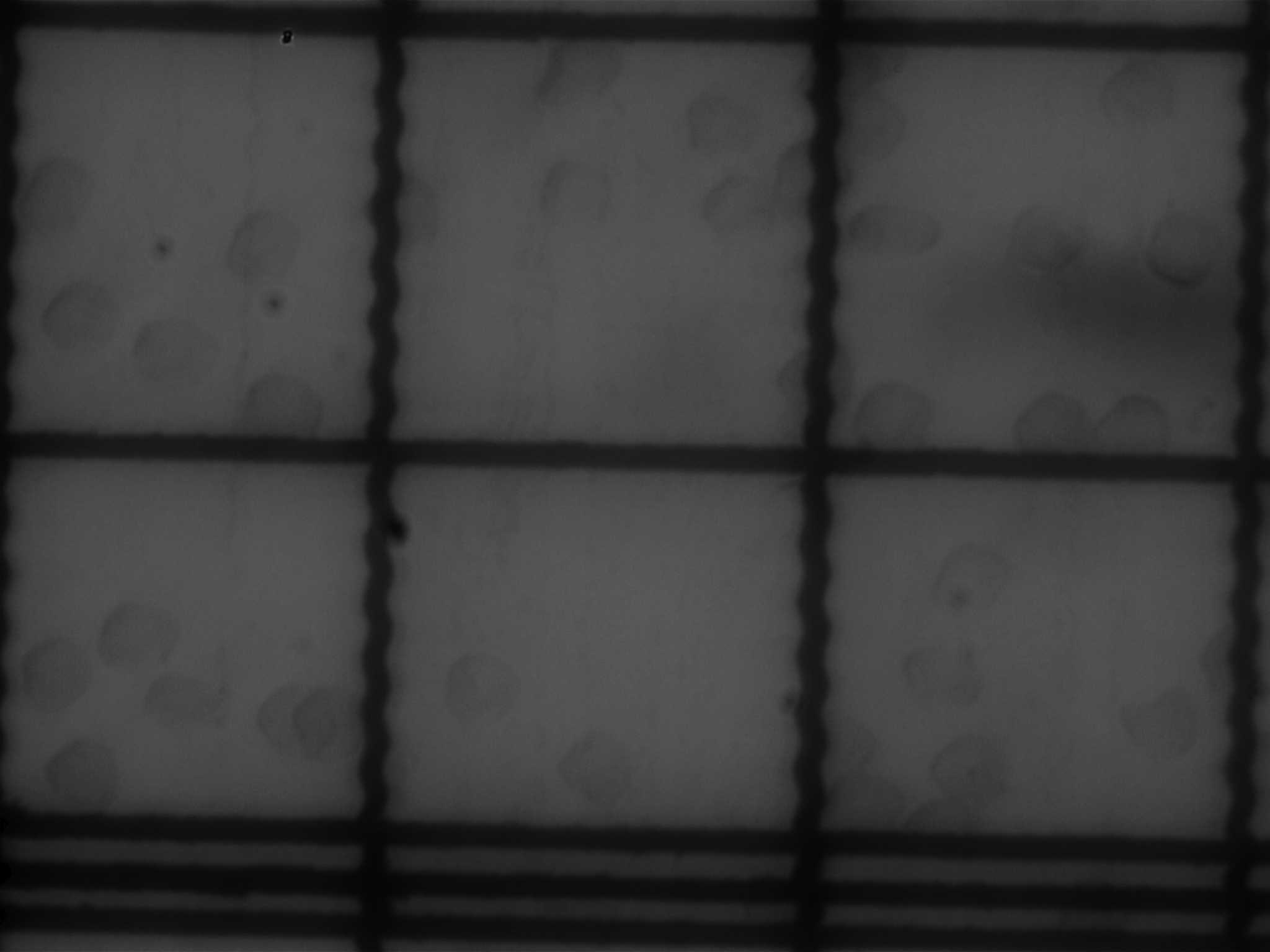

Supplement: S2 Image — (ZIP) [file pone.0226640.s005.zip › S2_imageseq_8_1.0_C1/C1-0029.png]

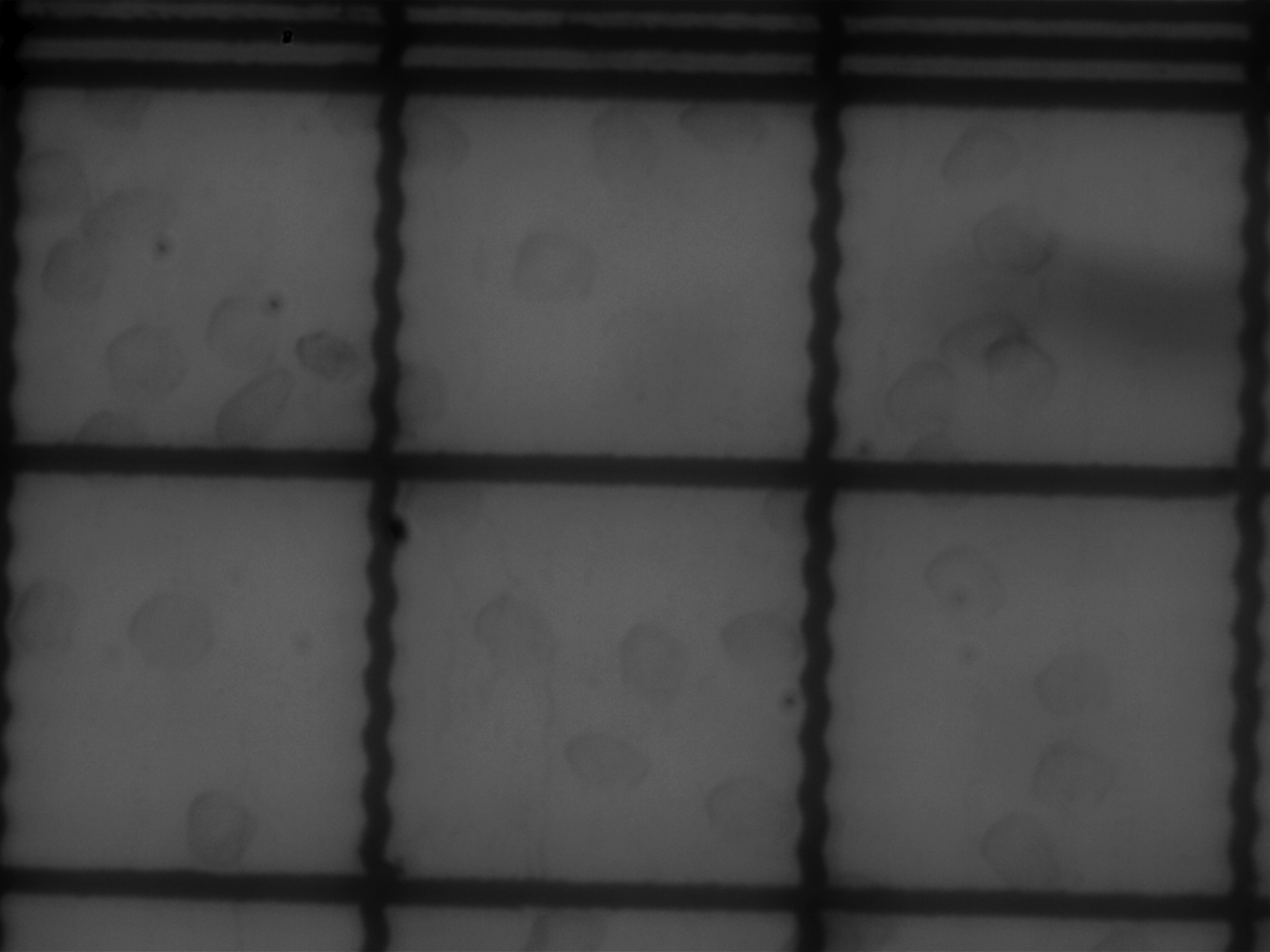

Supplement: S2 Image — (ZIP) [file pone.0226640.s005.zip › S2_imageseq_8_1.0_C1/C1-0030.png]
